# A thorough experimental study of CH/π interactions in water: quantitative structure–stability relationships for carbohydrate/aromatic complexes†
†Electronic supplementary information (ESI) available: Synthesis of disaccharides and aldehydes, NMR competition experiments and theoretical calculations. Fig. S1–S11. Tables S1 and S2. See DOI: 10.1039/c5sc02108a


**DOI:** 10.1039/c5sc02108a

**Published:** 2015-07-30

**Authors:** Ester Jiménez-Moreno, Gonzalo Jiménez-Osés, Ana M. Gómez, Andrés G. Santana, Francisco Corzana, Agatha Bastida, Jesus Jiménez-Barbero, Juan Luis Asensio

**Affiliations:** a Instituto de Química Orgánica (IQOG-CSIC) , Juan de la Cierva 3 , 28006 Madrid , Spain . Email: juanluis.asensio@csic.es ; Fax: +34 915644853 ; Tel: +34 915622900; b Dept. Química and Centro de Investigación en Síntesis Química , Universidad de La Rioja , Logroño , Spain; c Institute of Biocomputation and Physics of Complex Systems (BIFI) , University of Zaragoza , BIFI-IQFR (CSIC) , Zaragoza , Spain; d Centro de Investigaciones Biológicas (CIB-CSIC) , Madrid , Spain; e Center for Cooperative Research in Biosciences (CIC-bioGUNE) , Derio-Bizkaia , Spain; f Basque Foundation for Science , Ikerbasque , Bilbao , Spain

## Abstract

CH/π interactions play a key role in a large variety of molecular recognition processes of biological relevance. However, their origins and structural determinants in water remain poorly understood. In order to improve our comprehension of these important interaction modes, we have performed a quantitative experimental analysis of a large data set comprising 117 chemically diverse carbohydrate/aromatic stacking complexes, prepared through a dynamic combinatorial approach recently developed by our group. The obtained free energies provide a detailed picture of the structure–stability relationships that govern the association process, opening the door to the rational design of improved carbohydrate-based ligands or carbohydrate receptors. Moreover, this experimental data set, supported by quantum mechanical calculations, has contributed to the understanding of the main driving forces that promote complex formation, underlining the key role played by coulombic and solvophobic forces on the stabilization of these complexes. This represents the most quantitative and extensive experimental study reported so far for CH/π complexes in water.

## Abstract 2

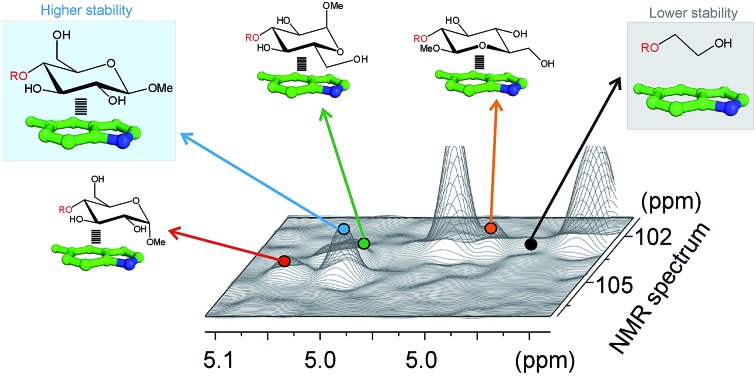
A dynamic combinatorial analysis of carbohydrate/aromatic complexes clarifies the structural determinants and origins of these important interactions in water.

## Introduction

Biomolecular recognition processes are mediated by a wide range of weak intermolecular forces, that cooperatively act to provide binding affinity and specificity. Among them, CH/π bonds have attracted considerable attention in recent years due to their importance and widespread occurrence.^1^–^12^ These interactions can be generally described as the attractive molecular force occurring between polarized CH fragments and aromatic rings. According to theoretical studies, in the gas phase, a major dispersive component, together with a smaller, but still significant, electrostatic contribution (which increases with the polarization of the involved CH groups) determine the stability of the resulting complexes.^2^,^3^ However, the structural determinants and driving forces behind CH/π bonds in water have proved more difficult to dissect and remain poorly understood.

High dielectric solvents are expected to attenuate coulombic interactions between the CH moieties and the aromatic systems. Intriguingly, we have shown in a recent study that, even in water, CH/π contacts are strongly stabilized by CH polarization, suggesting that electrostatic forces remain remarkably relevant.^5^ Since this contribution increases in low dielectric environments, CH/π complexes might be expected to be stronger in organic solvents. However, it is well established that the addition of organic co-solvents usually has a strongly unfavourable influence on the interaction.^2^,4d,6a,7a This phenomenon has traditionally been considered as evidence for the relevance of the hydrophobic effect in the stabilization of the complex. However, it might also be explained by competitive CH/π contacts between the substrates and co-solvent molecules, as pointed out by Nishio and co-workers.^2^

Regarding the van der Waals contribution, while certainly dominant in the gas phase, its significance and relative weight in solution remains unclear. Indeed, recent experimental measurements of alkyl–alkyl interactions in solution seem to be consistent with a large cancellation of dispersion forces, due to competitive interactions with the solvent.^13^ This attenuation is expected to be highly dependent on the molecular properties of the involved species and its magnitude for CH/π bonds is presently unknown.

Finally, from a design perspective, a quantitative description of the most relevant features of these complexes and their influence on the binding free energies would be well received. Such an analysis should explicitly consider a wide range of variations in both the CH/π donor and acceptor molecules (see below).

In summary, after more than twenty years of scientific research on this topic, a detailed and quantitative picture of the origin and structure–stability relationships that govern these important interaction modes in water is still missing. Such a description might have a significant impact in different fields, such as chemical biology, supramolecular chemistry, and medicinal chemistry, facilitating the design of improved ligands, receptors, foldamers, or new materials.

**Fig. 1:**
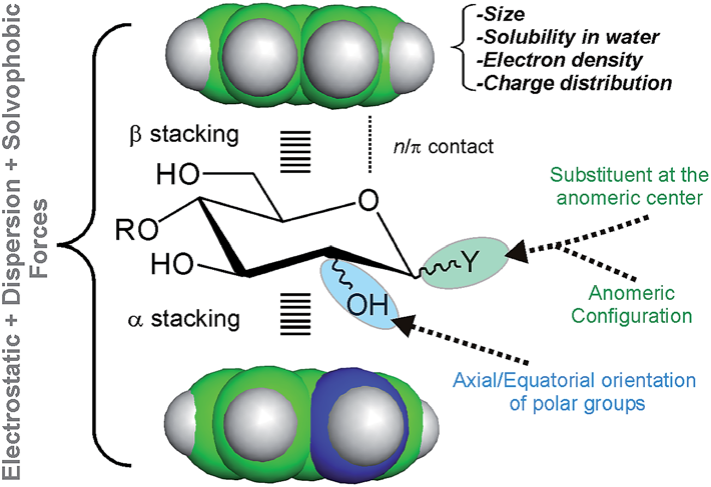
Schematic representation of the main structural features that determine the stacking free energy in carbohydrate/aromatic CH/π complexes.

In order to improve our understanding of CH/π complexes in water, we have carefully studied a paradigmatic example of biological relevance: carbohydrate/aromatic stacking (Fig. 1).^6^–^12^ This particular interaction mode plays a key role in the molecular recognition of glycans by antibodies, enzymes, lectins, and nucleic acids and typically involves the simultaneous formation of 2–3 intermolecular bonds between pyranose CH groups and receptor aromatic units. It should be noted that carbohydrate/aromatic contacts can involve the α and/or β faces of the pyranose ring (herein referred to as α- and β-type complexes, respectively) and that different axial/equatorial orientations are also feasible for the polar OH moieties. Moreover, the aromatic systems can also exhibit a wide range of chemical properties (electron density, charge distribution, solubility), conferring a significant structural diversity to the stacking complexes (Fig. 1).

We have employed a dynamic combinatorial protocol recently developed by our group^5^,8a to analyze a large data set of 117 chemically diverse carbohydrate/aromatic complexes. This represents the most quantitative and extensive experimental study reported so far for CH/π complexes in water. The obtained experimental data, in combination with quantum mechanical calculations, provide a detailed picture of the structure–stability relationships that govern these complexes, and contribute to the clarification of their origin.

## Results and discussion

### A. Brief description of the dynamic combinatorial approach and designed libraries

**Fig. 2:**
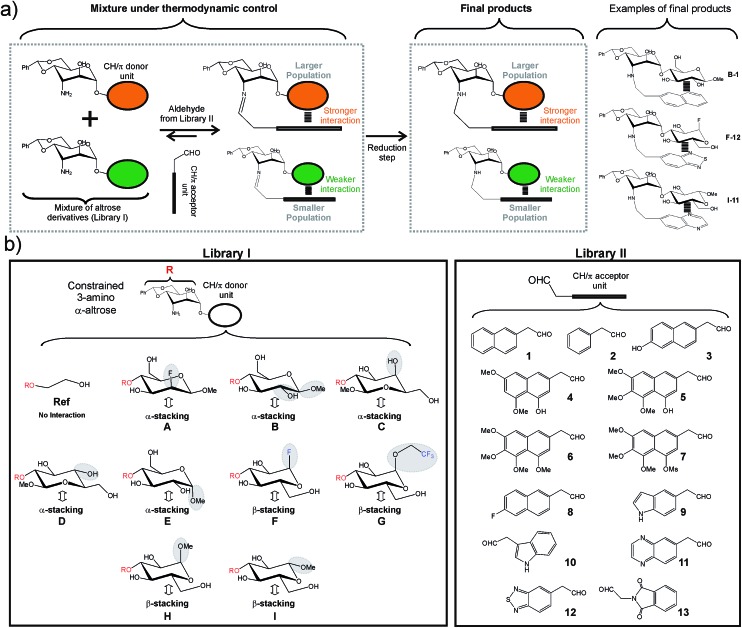
(a) Schematic representation of the employed dynamic combinatorial strategy (see the main text). Examples of the products obtained upon reductive amination of disaccharides **A–I** with aldehydes **1–13** are shown on the right. (b) Libraries of disaccharide (library I, compounds **A–I**) and aldehyde (library II, compounds **1–13**) derivatives synthesized for our analyses. For library I diverse functions and/or stereochemistries were considered for both anomeric and non-anomeric positions. The most relevant variations at these key sites are highlighted with grey ellipses (see the main text). The interacting face (α or β) of the different CH/π donor units is indicated with a double-headed arrow.

The employed methodology has been previously described and is briefly outlined in Fig. 2a.8*a* First, libraries I and II were designed and synthesized (Fig. 2b and S1–S2†). Library I included nine disaccharide derivatives (**A–I**) formed by a common 3-deoxy-3-amino α-altrose scaffold linked to alternative CH/π donor units. Similarly, library II included thirteen acetaldehyde derivatives (**1–13**) equipped with different CH/π acceptor aromatic systems. It should be noted that for any given disaccharide/aldehyde pair, one particular imine/hemiaminal adduct could be formed. Interestingly, according to previous studies,8*a* these species are stabilized by well-defined intra-molecular CH/π contacts, involving the aromatic system and only one (α or β) face of the donor fragment (Fig. 2a and b and S3†). Thus, disaccharides with α(1–2) (**C**, **D**) or α(1–4) (**A**, **B** and **E**) bonds form α-type complexes upon reaction with the aromatic aldehyde, while those with α(1–3) linkages (**F**, **G**, **H** and **I**) establish β-type complexes. Our library also included CH/π donor units with different axial/equatorial orientations for the key OH/F positions (Fig. 2b). All these modifications were selected to evaluate specific structural features of the interaction modes and their influence on stability. These include:

(a) The relative orientation of the pyranose and aromatic units (compound **B***vs.***D**).

(b) The equatorial or axial orientation of those polar groups located on non-interacting CH positions (compounds **A***vs.***B** and **C***vs.***D**).

(c) The presence of axial polar groups on the interacting pyranose face (compounds **B***vs.***E**).

(d) The participation of the α or β pyranose face in the stacking interaction (**C***vs.***H** and **D***vs.***I**).

(e) The configuration of the anomeric centre in β-type complexes (**H***vs.***I**).

(f) The presence of electron-withdrawing substituents at the anomeric centre in β-type complexes (**H***vs.***F** or **G**).

Finally, a reference compound (**Ref**) lacking a CH/π donor pyranoside was also prepared.

Regarding library II, aromatic systems with different sizes, orientations, electronic properties and water solubility were explicitly considered (Fig. 2b).

Equimolecular mixtures of two or more model disaccharides in buffered water solutions were treated with sub-stoichiometric amounts of a given aldehyde to produce a dynamic mixture of the intermediate imines/hemiaminals (Fig. 2a). After equilibration, chemical reduction of the transient species with sodium cyanoborohydride yields a non-equimolecular mixture of secondary amines whose relative populations were evaluated by either 2D or 1D NMR spectroscopy. These populations have been shown to reflect those of the intermediate species^5^ and, therefore, were employed to determine the stability differences (ΔΔ*G*) among alternative stacking modes. Moreover, by including the reference altrose derivative **Ref** (for which no stacking is feasible) in the initial mixture, we could also determine the net interaction energies for the different complexes (Δ*G*).

This experimental approach outperforms more conventional methodologies, commonly based on binding studies with synthetic receptors4a,^6^ or wild-type/mutated proteins.7a,e,^10^ Firstly, it allows the analysis and quantification of CH/π bonds in the absence of additional interfering interactions (such as hydrogen bonds, van der Waals contacts, *etc.*). Secondly, it displays an unprecedented sensitivity. Our experience showed that population differences of down to 10% among the final products can be detected by NMR.8*a* At 277 K (the temperature selected for our experiments), this permits the quantification of ΔΔ*G* values as small as 0.05 kcal mol^–1^. This point is of capital importance when dealing with very weak forces, such as CH/π interactions. Finally, most experimental analysis of carbohydrate/aromatic complexes reported so far has been restricted to a few simple carbohydrate/aromatic pairs, with special attention given to those systems commonly found in nature. In contrast, our method is highly versatile and allows the incorporation of a large variety of CH/π donor and acceptor fragments.

### B. Experimental evaluation of stacking free energies

In order to estimate the CH/π donor properties of the different pyranose units, we first carried out combinatorial experiments with relatively complex mixtures, formed by up to 5–8 selected disaccharides and one single aldehyde. Relative populations of the final products were determined by integrating key representative signals in 2D-HSQC experiments.

**Fig. 3:**
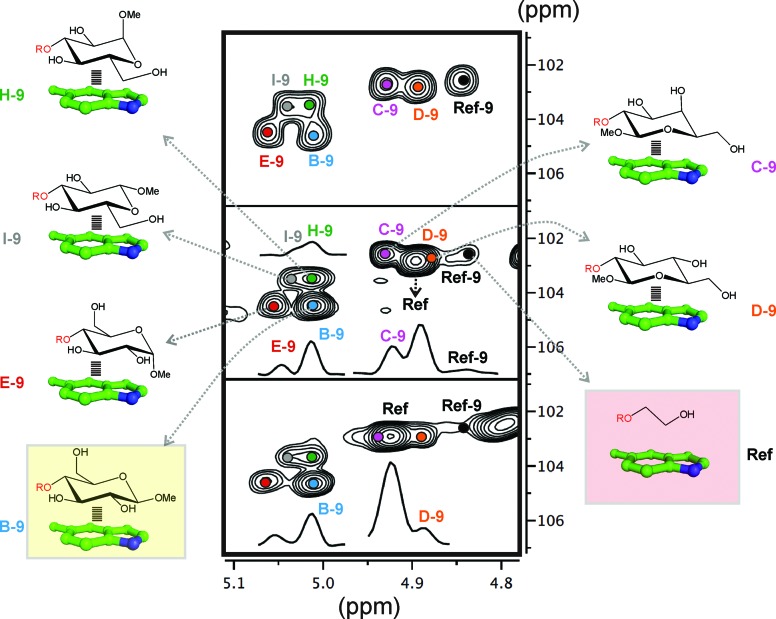
Dynamic combinatorial experiments followed by 2D-HSQC NMR (see the main text). Middle and lower panel show NMR data acquired at slightly different pH values to relieve the signal overlapping.

One example is represented in Fig. 3. In this particular case, an equimolecular mixture of disaccharides **B**, **C**, **D**, **E**, **H** and **I**, together with the reference compound (**Ref**) were treated with aldehyde **9** (see the Experimental section). In the presence of an excess of aldehyde, the reductive amination reaction yields an equimolecular mixture of the final disaccharide products that can be easily identified by 2D-HSQC experiments (Fig. 3 upper panel: the altrose anomeric region).

On the contrary, using a sub-stoichiometric amount of aldehyde **9**, the effective competition among the alternative CH/π donor units is established. Under these circumstances (middle and lower panels), the experiment yields unequal populations of the different disaccharide products, thus providing a qualitative indication of the stability differences among the carbohydrate/aromatic complexes. Thus, the data shows that glucose unit **B** forms the most stable complex with the indole ring (complex **B-9**), while the weakest interaction corresponds to **I**. As expected, the reaction product for the reference compound (**Ref-9**), with no carbohydrate/aromatic stacking, is barely detectable in solution.

Although the HSQC data provided valuable qualitative information, a more precise integration of the NMR signals required 1D ^1^H-NMR data collected with long acquisition delays. Therefore, simpler experiments, involving a single pair of altrose derivatives and one aldehyde at a time were performed. Specifically, we carried out competitions within all possible **Ref**/disaccharide pairs and the different aldehydes to obtain net interaction free energies (Δ*G*) for the alternative stacking modes. Moreover, we also determined the relative stabilities of all the complexes with respect to those formed by **D** (ΔΔ*G*), performing reactions with all possible **D**/disaccharide/aldehyde combinations. Finally, many other crosschecks were carried out with selected aldehydes and disaccharide pairs (Fig. S4†).

The preferred geometry of the CH/π complexes was deduced from the pattern of chemical shift perturbations promoted by the interacting pyranoses (Fig. S3 and S5–S7†). The interaction geometries were also estimated by molecular dynamics simulations in explicit solvent (MD, Fig. S8†).

**Fig. 4:**
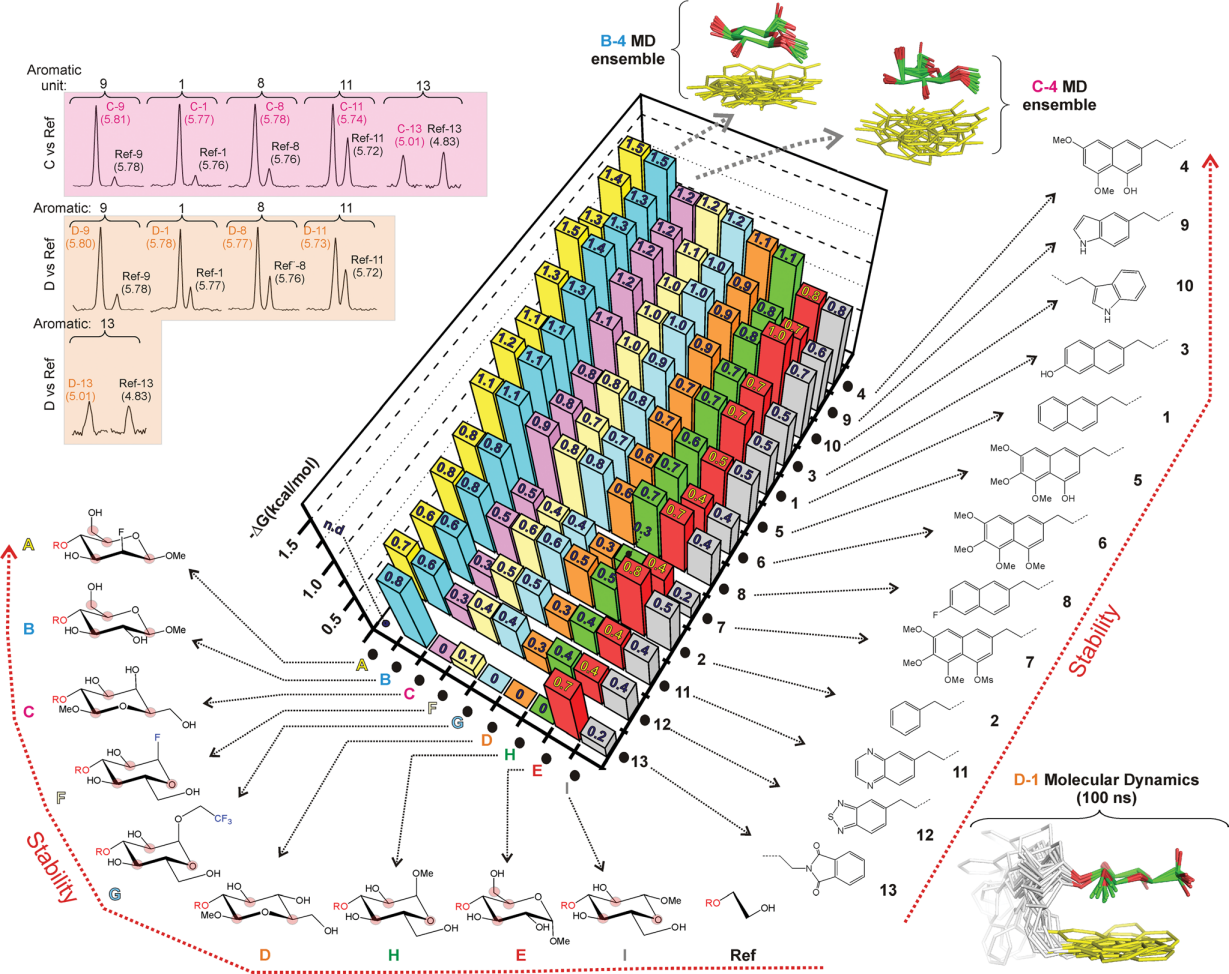
Net stabilities (Δ*G*, kcal mol^–1^) for the CH/π complexes established between donors **A–I** and the aromatic rings present in **1–13** measured from NMR pairwise competition experiments. Examples of experimental data sets are shown in the upper-left corner. The structural features of the different complexes were estimated from 100 ns MD simulations. Conformational ensembles obtained for products **B-4**, **C-4** and **D-1** through the MD simulations are also shown.

The obtained results, expressed as net stacking energies (Δ*G*_exp_ (kcal mol^–1^)), are schematically represented in Fig. 4 and collected in Table S1.† According to these data, the stability of carbohydrate/aromatic complexes in water is in the 0–1.5 kcal mol^–1^ range. These values are in close agreement with those reported in the literature for natural systems,7*a* and imply that every single CH/bond contributes up to 0.5–0.7 kcal mol^–1^ to the interaction free energy.

### C. Structure–stability relationships: influence of pyranose and the aromatic chemical nature

**Fig. 5:**
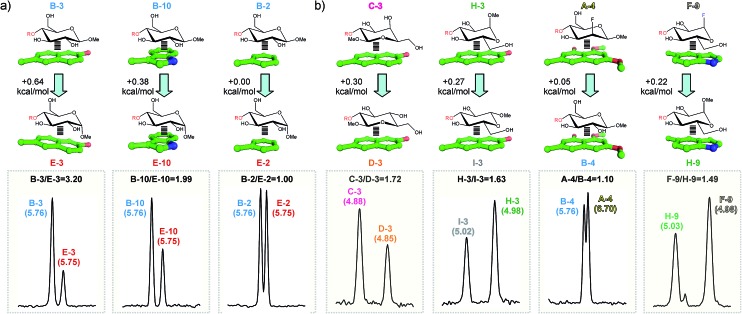
Pairwise competition experiments between selected disaccharide pairs and aldehydes ((a) and (b); see the main text). NMR signals for the altrose anomeric proton in the final products are shown in the bottom panel. Chemical shifts (ppm) are indicated above the signals together with the measured product ratios (black). The estimated free energy differences between the alternative CH/π complexes are shown in the upper panel.

**Fig. 6:**
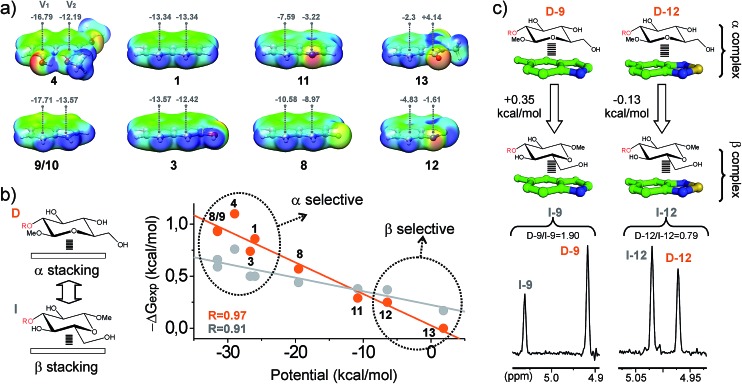
(a) Electrostatic potential surfaces calculated at the M06-2X/TXVP level for selected aromatic systems, together with the electrostatic potential values (kcal mol^–1^) at 2 Å above the centre of the rings (*V*_1_ and *V*_2_). (b) Illustration of Δ*G*_exp_*versus* the aromatic potentials (*V*_1_ + *V*_2_) for altrose derivatives **D** and **I**. Linear fits and correlation coefficients (*R*) are shown. (c) Pairwise competition experiments performed with the disaccharide pair **D**/**I** and both electron rich and poor aldehydes (**9** and **12**). NMR signals for the altrose anomeric proton in the final products are shown in the bottom panel, together with the measured product ratios (black). Free energy differences between the alternative CH/π complexes are given in the upper panel.

The results indicate that both the pyranose and the aromatic unit have a significant influence on the stability of the stacking complexes. Several trends are apparent (see Fig. 4–6):

(a) The larger the number of CH/π contacts, the stronger the complex. This simple rule explains why the aromatic system **2** establishes weaker interactions than the more extended naphthyl unit present in **1**. It also explains why derivative **B** forms more stable complexes than **C** or **D** (in the former case, the glucose hydroxymethyl group also contacts the aromatic ring; see Fig. S6†).

(b) An axially-oriented OMe group on the interacting face of the pyranose can be highly disruptive to complex formation. This effect is exemplified by the different stability exhibited by complexes **B-3** and **E-3** (0.64 kcal mol^–1^ larger for the former; see Table S1† and Fig. 5a). The energy penalty detected in **E-3** results from the steric conflict between the axial polar group and the aromatic system and, consequently, depends on the size and orientation of this ring. In agreement with this view, for a differently oriented indole ring the complex destabilization decreases down to 0.38 kcal mol^–1^ (**B-10***vs.***E-10**. Fig. 5a) while the smaller phenyl ring becomes virtually insensitive to the pyranose configuration (**B-2***vs.***E-2**).

(c) For non-interacting pyranose CH groups, axial polar substituents are generally preferred over equatorial ones. Complexes formed by pairs **C**/**D**, **A**/**B** or **H**/**I** provide examples of this rule. A simple explanation for this behaviour is that equatorial polar moieties might be involved in repulsive interactions with the aromatic unit, and/or partially desolvated upon complex formation. As expected, the magnitude of the energy penalty associated with the axial/equatorial inversion depends on the precise geometrical features of the complex, varying from 0.3 kcal mol^–1^ (see **C-3***vs.***D-3** in Fig. 5b) to <0.1 kcal mol^–1^ (see **A-4***vs.***B-4** in Fig. 5b. The structural differences among these complexes, inferred from Δ*δ* values, are outlined in Fig. S7†).

(d) For glycosides with all-equatorial substituents, the α-face is, in most cases, preferred for interaction over the β-face. This selectivity is exemplified by the extra stability displayed by **D** complexes with respect to those formed by **I** (Fig. 4 and Table S1†). It should be noted that, in the former case, complex formation is only mediated by CH/π bonds. In contrast, for β-type interactions, the endocyclic oxygen also becomes involved in a n/π bond with the aromatic unit. According to our data, this latter contact provides little contribution to the binding strength, being somewhat unfavourable for electron-rich aromatic systems.

(e) Interactions mediated by the pyranose β-face are sensitive to the electron density at the endocyclic oxygen, which, in turn can be modulated by attaching electron-withdrawing substituents to the anomeric centre. This effect is clearly illustrated by the larger stability displayed by **F** or **G** complexes with respect to those formed by **H** (see for example **F-9***vs.***H-9** in Fig. 5b).

(f) The stability of the CH/π bonds established by a simple aromatic ring (for example the naphthyl unit present in **1**) can be enhanced by the incorporation of oxygen substituents (Table S1, Fig. 4 and S9†). In agreement with this view, aromatic binding strengths follow the order **4** > **3** > **1**, as expected for increasingly electron-rich substrates. However, this simple strategy presents limitations. It can be observed that those complexes formed by densely oxygenated aromatic systems such as **5–7** display reduced Δ*G*_exp_ values. Interestingly, quantum mechanical calculations (Fig. S9†) show that, in contrast to **4**, the OR groups present in **5–7** are not co-planar with the naphthyl unit and multiple rotamers are possible. Inspection of the molecular models suggests that, for some of these conformational states, aromatic OR substituents severely restrict the internal mobility of the complex, imposing a significant entropic penalty on the recognition process (this hypothesis is supported by the theoretical evaluation of binding enthalpies for the different aromatic units, see below). Alternatively, they could participate in repulsive steric contacts with the pyranose unit.

(g) The obtained data strongly suggest that electrostatics have a large influence on the aromatic binding ability. To illustrate this point, we calculated the electrostatic potential surfaces of different aromatic systems (densely oxygenated and conformationally flexible systems **5–7** were not included in this analysis). Selected surfaces are represented in Fig. 6a, together with the electrostatic potential values at 2 Å above the centre of the rings (denoted as *V*_1_ and *V*_2_), which is approximately the equilibrium distance at which the CH interacts with the aromatic ring in the complexes (see quantum mechanical geometries below). It can be observed that, for any given disaccharide (as **D** and **I**; see Fig. 6b), Δ*G*_exp_ values correlate linearly with the sum of the aromatic electrostatic potentials *V*_1_ + *V*_2_. Indeed, according to these data, this simple parameter represents a reliable predictor of the ability of the aromatic unit to participate in CH/π interactions. Interestingly, the observed linear correlations exhibit distinct slopes for β-type and α-type complexes, implying that electron-rich aromatic aldehydes (as **9** or **4**) exhibit some selectivity for the α-face of the pyranose unit, while electron-poor units (as **12** or **13**) are slightly β-selective (Fig. 6b). This result was further corroborated with pairwise competition experiments using a selected disaccharide pair (**D** and **I**; see Fig. 6c) and both electron-rich and electron-poor aldehydes (**9** and **12**). A simple explanation for this behaviour is that, for β-type complexes, electron-poor aromatic rings promote more stabilizing donor-acceptor n/π bonds with the pyranose endocyclic oxygen. Alternatively, for α-complexes, acceptor–donor CH/π bonds are dominant.

### D. Dissecting the driving forces for the formation of CH/π complexes in water: electrostatic *vs.* dispersion components

According to current knowledge, CH/π interactions in the gas phase are dominated by dispersive forces. Intriguingly, we have shown that, in water, the aromatic binding strengths are strongly correlated with the electrostatic potentials (Fig. 6a and b). To get insights into the different contributions that stabilize CH/π complexes in this polar environment, a theoretical analysis was performed.

**Fig. 7:**
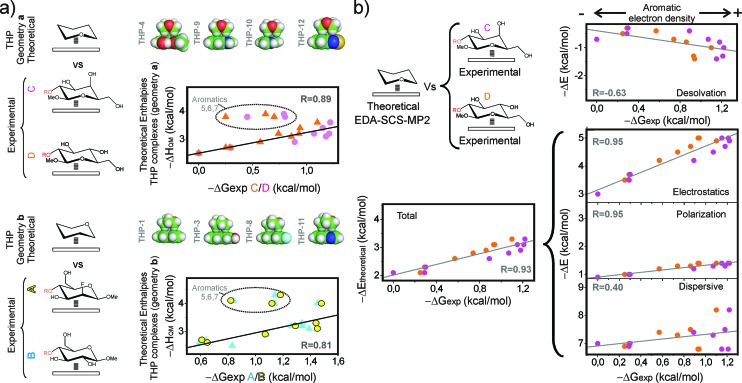
(a) Interaction enthalpies in water were calculated by quantum mechanics (M06-2X/TXVP level) for simplified inter-molecular complexes formed by tetrahydropyran and the aromatic units present in **1–13** assuming two alternative geometries (herein referred as (a) and (b); see the main text). These data are presented against the experimental free energy values (Δ*G*_exp_) measured for derivatives **C** and **D** (geometry (a), upper panel) and **A** and **B** (geometry (b), lower panel). Linear fits and correlation coefficients (*R*) are indicated. (b) Experimental Δ*G*_exp_ values measured for derivatives **C** (orange) and **D** (magenta) are presented against the gas phase interaction energies and the different contributions calculated at the SCS-MP2/6-311G(2d,p) level for the THP/aromatic complexes (see the main text). Linear fits and correlation coefficients are shown in all cases.

Quantum mechanical methods allowed us to assess the magnitude of the CH/π contacts established by the evaluated aromatic systems. Interaction enthalpies (Δ*H*_QM_) were calculated at the M06-2X/TZVP level^14^ in water (IEF-PCM method^15^) for simplified inter-molecular complexes formed by tetrahydropyran (THP, as a pyranose model) and the aromatic units present in **1–13** (Fig. 7a, Table S2, see ESI† for details). For each interacting pair, two orientations of the THP unit were considered (herein referred to as (a) and (b)) to simulate the alternative geometries of the CH/π contacts established by derivatives **C**/**D** and **A**/**B**/**E**, respectively, upon reductive amination with the aldehydes. A representation of the calculated Δ*H*_QM_ values *versus* the free energies (Δ*G*_exp_) measured for **C** and **D** (geometry (a), upper panel) and **A** and **B** (geometry (b), lower panel), together with the optimized geometries obtained for some of the complexes are shown in Fig. 7a. It can be observed that the most significant deviations between both data sets correspond to the densely oxygenated aromatic systems **5–7**. As previously mentioned, complex formation by these units could display a larger entropic cost, which explains the lack of correspondence between Δ*G*_exp_ and Δ*H*_QM_ values. For the rest of the aromatic systems, Δ*G*_exp_ correlates linearly with Δ*H*_QM_ with good correlation coefficients (see Fig. 7a).

The optimized geometries for the THP/aromatic complexes were employed to dissect the different contributions to the stability (Fig. 7b and Table S2†). To this aim, single-point interaction energies calculated at the SCS-MP2 (ref. 16)/6-311G(2d,p) level in the gas phase were partitioned into electrostatic, polarization, exchange, repulsion and dispersion contributions, through Localized Molecular Orbital Energy Decomposition Analysis (LMO-EDA).^17^ In addition, the penalties associated with the THP and aromatic desolvation were evaluated at the same theory level (it should be noted that these theoretical values do not account for solvophobic contributions; see the Experimental section for details).

These various contributions are shown *versus* the experimental Δ*G*_exp_ values measured in solution. The purpose of this comparison was to identify the main components of the interaction energies responsible for the experimentally observed trends. As an example, an illustration of the experimental Δ*G*_exp_ values measured for **C** and **D***versus* the interaction energies (both the total values and their contributions) calculated with SCS-MP2/6-311G(2d,p) level is shown in Fig. 7b. Linear fits and correlation coefficients are also shown in all cases (see also Fig. S10†). As observed in these plots, the predicted interaction energies show a good correspondence with the experimental Δ*G*_exp_ values. Regarding the energy contributions, different trends are apparent:

(a) Firstly, a weak and negative correlation is deduced for the energy penalty associated to the desolvation processes that accompany binding. This observation can be rationalized by considering that the stronger complexes usually involve more polar aromatic units, which in turn are more strongly solvated by water.

(b) Secondly, dispersion forces prevail among favourable contributions in the gas phase. However, this term shows a poorer correlation with the solution experimental free energies (Δ*G*_exp_) than the electrostatic and polarization terms. Of note, some of the most stable complexes (such as **9** and **10**, involving an indole unit) display weaker dispersive forces. In addition, this contribution exhibits little variation for most of the complexes.

(c) Finally, electrostatic and polarization contributions display a fairly good positive correlation with the experimental stabilities and show larger variations between strong and weak complexes.

In summary, although dominant in the gas phase, dispersion forces do not seem to account for the observed Δ*G*_exp_ variations in solution. On the contrary, these can be adequately justified in terms of electrostatic and polar interactions. All these observations strongly suggest that the experimental trends are, to a significant extent, dictated by electrostatics. Therefore, this term should be considered as an important determinant for the formation of CH/π bonds in water. In summary, our results indicate that, even in high dielectric environments, coulombic forces represent a key modulating influence for these complexes and, consequently, can be employed to predict and fine-tune the relative binding strengths.

### E. Dissecting the driving forces for the formation of CH/π complexes in water: evidence for the relevance of solvophobic contributions

**Fig. 8:**
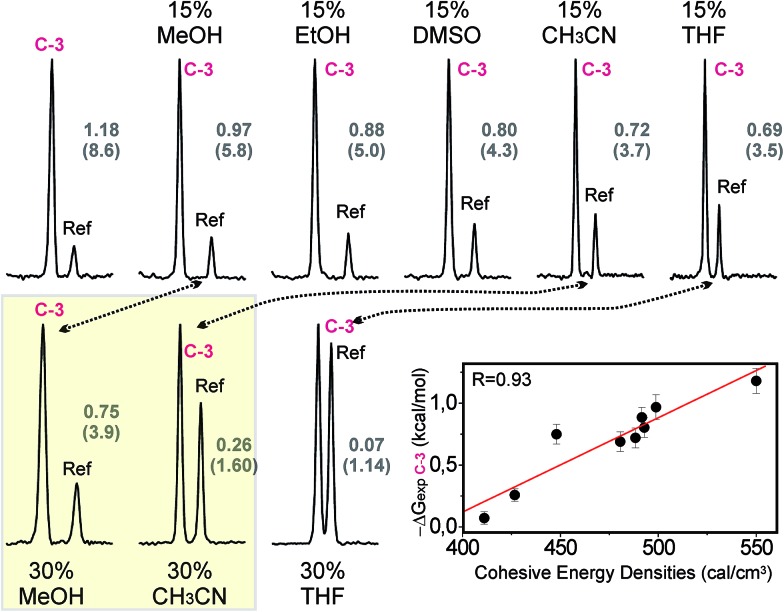
Pairwise competition experiments performed with pair **C**/**Ref** and aldehyde **3** in the presence of organic co-solvents (indicated in black). NMR signals for the altrose anomeric proton in the final products are shown. Product ratios (in brackets) together with the **C-3** complex stabilities under the different solvent conditions are indicated in grey. Differences in the destabilization induced by methanol and acetonitrile are highlighted with a yellow square. An illustration of the measured stabilities *versus* the cohesive energy densities of the different mixtures is shown in the bottom-right corner. Linear fit and correlation coefficient (*R*) are shown.

The role of solvophobic forces on the stabilization of CH/π bonds in water was also evaluated. To this aim, we measured the effect of adding organic co-solvents on the strength of a selected complex (**C-3**). Fig. 8 shows some of the pairwise competition experiments carried out with pair **C**/**Ref** and aldehyde **3** in the presence of increasing fractions (in the 0–30% range) of various organic co-solvents (indicated in black).

The data show that the addition of organic co-solvents is, in all cases, highly disruptive to the formation of the CH/π complex, with notable differences in the magnitude of the destabilization. Thus, 30% methanol decreases the free energy of association (Δ*G*_exp_) from 1.18 down to 0.75 kcal mol^–1^. In contrast, the identical fraction of THF renders the interaction almost undetectable (Δ*G*_exp_ < 0.1 kcal mol^–1^). Overall, the disruptive power of the solvents follows the order MeOH < EtOH < DMSO < acetonitrile < THF. This observation is fully consistent with the solvent effects in carbohydrate binding by synthetic receptors, recently reported by Davis and co-workers.6*a*

It should be noted that this experimental trend does not correlate with the dipolar moment (*μ*_DMSO_ = 3.96 > *μ*_acetonitrile_ = 3.92 > *μ*_H_2_O_ = 1.85 > *μ*_THF_ = 1.75 > *μ*_MeOH_ = 1.70 > *μ*_EtOH_ = 1.69) or dielectric constant (*ε*_H_2_O_ = 80.0 > *ε*_DMSO_ = 46.7 > *ε*_acetonitrile_ = 37.5 > *ε*_MeOH_ = 33.0 > *ε*_EtOH_ = 24.5 > *ε*_THF_ = 7.5) of the different solvent molecules. Moreover, it can not be easily rationalized by only invoking competitive solute/solvent interactions (in fact, the relative stabilities of the alternative solvent/naphthalene complexes, estimated through quantum mechanics, do not provide support for this hypothesis; see Fig. S11†).

In order to demonstrate the relevance of the hydrophobic effect as a driving force for complex formation, the relationship between the carbohydrate/aromatic interaction energies (Δ*G*_exp_) and the cohesive energy densities of the solvent media was evaluated (Fig. 8).^13^ It can be observed that the stronger interactions correspond to higher cohesive energy densities, while a decrease in this parameter usually implies a destabilization of the complex. In fact, both Δ*G*_exp_ values and cohesive energy densities exhibit an excellent linear correlation (correlation coefficient *R* = 0.93).

According to these data, the destabilizing influences of the organic co-solvents cannot be explained just in terms of solute/solvent competitive contacts. On the contrary, they seem to rely on a delicate balance between solute/solvent and solvent/solvent interactions. More specifically, the co-solvent’s ability to reduce the cohesive energy density of the mixture is a key feature to this process. Indeed the key influence of this parameter on the solvation of non-polar solutes has been recently demonstrated.^18^ Our results confirm that solvophobic forces play an important role in the stabilization of CH/π bonds in water.

## Conclusions

An extensive analysis of CH/π interactions in water has been performed. The employed dynamic combinatorial methodology has allowed the accurate determination of the binding free energies for 117 isolated carbohydrate/aromatic complexes, presenting a large diversity of chemical features at both the CH/π donor and acceptor units. These data have revealed the most relevant structure–stability relationships that govern complex formation, opening the door to the rational design of improved carbohydrate-based ligands or carbohydrate receptors.

From a more fundamental perspective, our analysis has provided insights into the origin of these contacts. In particular, our results highlight the key role of electrostatics on the stabilization of CH/π complexes in water. Indeed, most of the free energy variations herein described can be rationalized in terms of simple coulombic interactions between the pyranose and the aromatic units. Thus, in most cases, attractive or repulsive polar interactions can be invoked to explain how the stability of the complexes is modulated by the orientation of the pyranose electron-withdrawing substituents. In addition, it also clarifies the observed facial selectivity and how this parameter is affected by the electron-rich or electron-poor character of the aromatic unit. Finally, the relative binding strengths of the different aromatic systems (in the 0–1.5 kcal mol^–1^ range) can be anticipated from their electronic properties.

Regarding dispersion forces, they have been traditionally considered to be dominant in the gas phase. However, according to recent studies this consideration might not hold in a physiological environment.^13^ While their relative weight in solution cannot be inferred from this work, our results suggest that this term exhibits smaller variations for systems of similar sizes and, therefore can not be employed to effectively tune the binding free energies.

Finally, we have demonstrated that the strength of the CH/π bonds does not depend only on the donor/acceptor contacts. Indeed, solute/solvent and, most importantly, solvent/solvent interactions have to be taken into account. Our results indicate that solvophobic interactions provide a significant contribution to the complex stability.

Overall, the data presented herein provide key clues, at the general and specific levels, to understand and modulate biomolecular interaction processes in which aromatics are involved, with particular interest for the molecular recognition of glycans.

## Supplementary Material

Supplementary information
