# Supplementary material for: A thorough experimental study of CH/π interactions in water: quantitative structure–stability relationships for carbohydrate/aromatic complexes
Source: Chem Sci. 2015 Jul 30;6(11):6076–85. doi: 10.1039/c5sc02108a (PMC5504637; doi:10.1039/c5sc02108a)

Electronic Supplementary Material (ESI) for Chemical Science.
This journal is © The Royal Society of Chemistry 2015

Supporting Information

For

A thorough experimental study of CH/π interactions in water: Quantitative structure-stability relationships for carbohydrate/aromatic complexes

Ester Jiménez-Moreno, Gonzalo Jiménez-Osés, Ana M. Gómez, Andrés G. Santana, Francisco Corzana Agatha Bastida, Jesus Jiménez-Barbero, Juan Luis Asensio*.

CONTENTS:

Experimental Section
Tables S1 and S2
Figures S1 to S11

1. General Information

Chemicals were purchased from commercial sources and were used without further purification. All solvents were purified by distillation over drying agents or by elution through a PURE SOLV purification system. Unless stated otherwise, reactions were carried out under a dry argon atmosphere in vacuum-flame dried glassware. Residual water was removed from starting compounds by repeated coevaporation with toluene. Analytical thin layer chromatography was carried out using pre-coated, aluminium backed plates (Merck Kieselgel 60 F254). Detection was by examination under UV light (254 nm) and then by charring with 10% sulfuric acid in ethanol or cerium-ammonium-molybdate. Flash column chromatography was performed using silica gel [Merck, 230−400 mesh (4−63 μm)]. Extracts were concentrated in *vacuo* using both a Buchi rotary evaporator (bath temperatures up to 40 °C) at a pressure of 15 mmHg (diaphragm pump) and a high vacuum line at room temperature. ^{1}H NMR and ^{13}C NMR spectra were measured in the solvent stated at 500, 400 or 300 and 126, 101 or 75 MHz, respectively. Chemical shifts are quoted in parts per million from residual solvent peak and coupling constants (J) given in Hertz. Multiplicities are abbreviated as: b (broad), s (singlet), d (doublet), t (triplet), q (quartet), m (multiplet) or combinations thereof. An Agilent 6520 accurate-mass quadrupole time-of-flight (Q-TOF) mass analyzer was used for the HR-MS. Glycosyl donor was prepared from allyl 3-azido-3-deoxy-4,6-*O*-benzylidene α-D-altropyranose[1] by acetylation, allyl cleavage at the anomeric position, and trichloroacetimidate formation according to standard procedures. Starting glycosyl acceptors **A1-I1** were prepared following well-established procedures, and cleavage of protecting hydroxyl groups was carried out according standard procedures.[2]

2. General Procedures

***2.1 General experimental procedure for mesylation**:* A solution of the corresponding alcohol (1 mmol) in anhydrous CH_2Cl_2 (10 mL) cooled to 0 ºC and under inert atmosphere was treated with triethylamine (3 mmol) and mesyl chloride (1.2 mmol). The mixture was stirred until TLC showed no starting alcohol was left and was then poured onto a saturated aqueous sodium bicarbonate solution. The organic phase was separated and the aqueous layer was extracted twice with CH_2Cl_2. The combined organics layers

[1] R. H. Plet, A. K. Sandhu, M. Sehailia, M. J. Porter, *Synlett* 2009, 3258.
[2] P. J. Kocienski, *Protecting Groups*, 3rd Edn., 2005, Georg Thieme Verlag, Stuttgart.

were washed with water, dried over anhydrous Na_2SO_4, filtered and concentrated under *vacuum*. Crude mesylates were used in the following step without further purification.

2.2 General experimental procedure for displacement with cyanide: A solution of the compound with the appropriate leaving group (1 mmol) in anhydrous DMF (10 mL) was treated with KCN (4 mmol) under inert atmosphere. The reaction mixture was allowed to react at the corresponding temperature (rt to 90ºC) until TLC showed completion of the reaction. The mixture was then poured onto a saturated solution of NH_4Cl and extracted twice with CH_2Cl_2. The organic layer was washed with water and brine, dried over anhydrous Na_2SO_4 and evaporated to afford a residue that was purified by flash chromatography.

2.3 General experimental procedure for nitrile reduction with DIBAL-H: To a solution of the corresponding nitrile (1 mmol) in anhydrous CH_2Cl_2 (10 mL) at -78 ºC under Ar was added dropwise a solution of DIBAL-H 1M in toluene (2-4 mmol). The reaction was stirred allowing the temperature to raise until -50 ºC and after 2-3 hours (unless stated otherwise) EtOAc was added to destroy the excess of the reducing reagent. When the reaction has reached room temperature, a 1:1 mixture of acetic acid and water was added and it was stirred for additional 5 minutes. The mixture was then neutralized with a saturated solution of sodium bicarbonate and extracted with CH_2Cl_2. The organic phase was washed again with a saturated solution of sodium bicarbonate and brine, dried over anhydrous Na_2SO_4 and concentrated under vacuum. Finally, the residue was purified by flash chromatography

2.4 General experimental procedure for Dess-Martin oxidation. To a solution of the alcohol (1 mmol) in CH_2Cl_2 (6 mL) or CH_2Cl_2/THF (1:1, 6 mL) under argon atmosphere, Dess Martin Periodinane (1.1 mmol) was added. The reaction was stirred at r.t. until TLC showed no starting alcohol was left. Then, it was poured onto a saturated solution of sodium bicarbonate and extracted twice with CH_2Cl_2. The organic layers were washed with brine, dried over anhydrous Na_2SO_4 and concentrated at reduced pressure. The residue was purified by flash chromatography.

2.5 General experimental procedure for the Wittig reaction. To a suspension of (methoxymethyl)triphenylphosphonium chloride (4 mmol) in anhydrous THF (6.6 mL) under argon atmosphere and cooled to 0ºC, BuLi (1.6 M in hexane, 3.96 mmol) was added dropwise and the mixture was stirred for 30 minutes. A solution of the corresponding aldehyde (1 mmol) in anhydrous

THF (2.2 mL) was then added at 0 ºC and the reaction mixture was allowed to warm to room temperature and stirred until no starting aldehyde was left (monitorized by TLC). Acetone was added to quench the excess of reagent and stirred for 5 min. Addition of Et_2O produced a solid precipitate that was filtered off. The filtrate was subsequently washed with a saturated solution of sodium bicarbonate and brine, dried over anhydrous Na_2SO_4 and concentrated. The residue was purified by flash chromatography.

2.6 General experimental procedure for the hydrolysis of vinyl ethers. A solution of the corresponding methyl vinyl ether (1 mmol) in CH_2Cl_2 (10 mL) containing 10 % TFA was stirred at room temperature until no starting material was left. The solvent was removed at reduced pressure and the residue purified by flash chromatography.

***2.7 General experimental procedure for glycosidation**:* A stirred solution of the glycosyl donor **D** (3.0 mmol) and the appropriate glycosyl acceptor **A** (1.0 mmol) in anhydrous CH_2Cl_2 (50 mL) under argon at -65ºC (unless otherwise noted), was treated with TMSOTf (0.2 mmol) and allowed to stir at this temperature until no glycosyl acceptor was left (monitored by TLC). Et_3N was then added and the resulting mixture was vigorously stirred for 10 min. The reaction mixture was then concentrated, without heating, and the crude product was purified by flash chromatography.

2.8 General experimental procedure for oxidative cleavage of the p-methoxybenzyl groups: *1. Method A***.** To a solution of the *p*-methoxybenzyl ether (1.0 mmol) in a 8:1:1 CH_2Cl_2/MeOH/H_2O mixture (50 mL) was added DDQ (2.0 mmol/ *p*-methoxybenzyl group) and then stirred at rt. for 3-6 h. The crude was chromatographed using CH_2Cl_2/MeOH 0→10%. *2. Method B*[3]. To a solution of the corresponding *p*-methoxybenzyl ether (1.0 mmol) and di-*tert*-butyl pyridine (3.0 mmol/*p*-metoxybenzyl group) in CH_2Cl_2/H_2O (18:1, 55 mL) was added DDQ (1.5 mmol/*p*-methoxybenzyl group). The reaction mixture was vigorously stirred overnight and concentrated *in vacuo*. The residue was then purified by column chromatography.

---

[3] H. M. Kim, I J. Kim and S. J. Danishefsky, *J. Am. Chem. Soc.* 2001, **123**, 35.

***2.9 General experimental procedure for cleavage of the p-methoxybenzylidene group*[4]:** To a solution of the corresponding *p*-methoxybenzylidene containing compound (1 mmol) in CH_3CN containing 10% water (30 mL) was added Selectfluor (2.4 mmol) at rt.. The reaction mixture was allowed to react at appropriate temperature. Finally, the solvent was removed *in vacuo* and the residue purified by flash chromatography.

***2.10 General experimental procedure for selective opening of the p-methoxybenzylidene group*[5]:** A solution of trifluoroacetic acid (15 mmol) in DMF (4 mL) cooled to 0ºC was added dropwise to a stirred mixture of the corresponding *p*-methoxybenzylidene containing compound (1 mmol), sodium cyanoborohydride (10 mmol) and 3Å molecular sieves (2 g) in DMF (10 mL). The reaction was stirred overnight at rt. and then filtered through Celite. The filtrate was poured into ice-cold saturated aqueous sodium hydrogen carbonate solution. The aqueous layer was extracted three times with CH_2Cl_2 and the combined organic layers were washed with brine, dried over anhydrous Na_2SO_4, filtered and concentrated under vacuum. The residue was purified by flash chromatography.

***2.11 General experimental procedure for reduction of azide group through a Staudinger reaction with concomitant acetate hydrolysis*:** A solution of the corresponding azide (1 mmol) in THF (32 mL) was treated with aqueous 0.1 M NaOH (2 mL) and PMe_3 (1.5 mmol, 1 M solution in THF). The mixture was then stirred at rt. until TLC showed no starting azide was left. The solvent was removed in *vacuo* and the residue purified by flash chromatography.

3. Synthetic approach to the 2-aryl-/hetaryl-acetaldehyde library (3-12)

The required 2-aryl/hetarylacetaldehydes were prepared using the appropriate benzyl-type alcohols as starting materials and following either of the routes shown in the scheme below (Scheme 1). In route *a*, elongation was carried out by displacement at the benzylic position of an appropriate leaving group (mesylate, bromide or benzoate) with cyanide anion and subsequent DIBAL-H reduction. Alternatively, in route *b*, elongation was achieved using a sequence of reactions including successive Dess-Martin oxidation, a Wittig-type homologation and vinyl ether hydrolysis.

---

[4] J. Liu and C.-H. Wong, *Tetrahedron Lett.,* 2002, **43**, 4037.

[5] C. Grandjean and G. Lukacs, *J. Carbohydr. Chem.* 1996, **15**, 831.

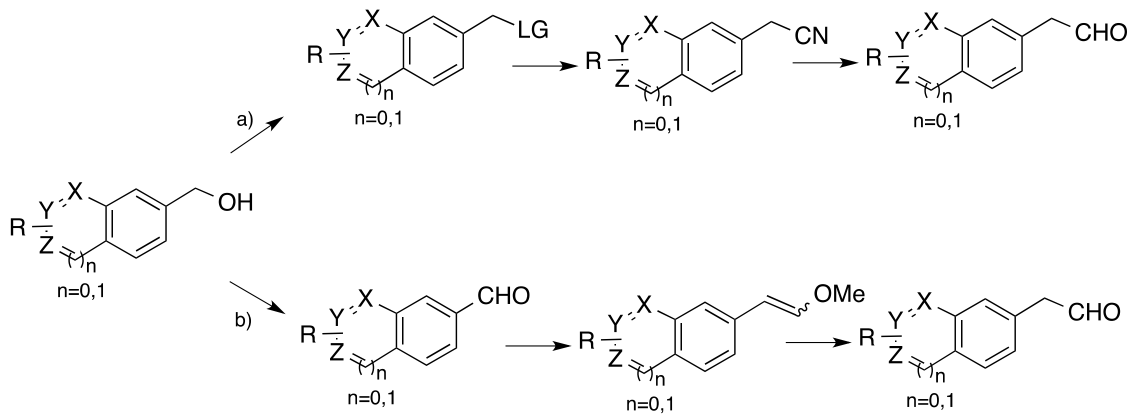

Scheme 1. Alternative routes for the preparation of the 2- aryl-acetaldehydes library

4. Preparation and Characterization of 2- aryl-acetaldehydes

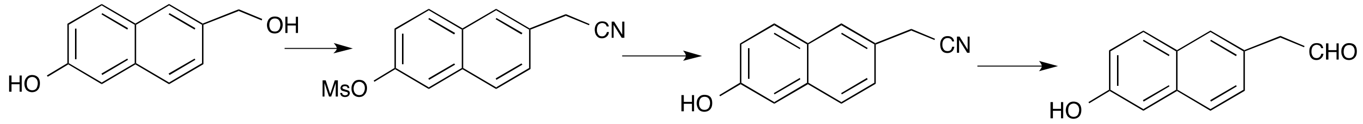

3a **3b** **3c** **3**

Scheme 2. Synthetic route for the preparation of 2-(6-hydroxynaphthalen-2-yl)acetaldehyde **3.**

6-(cyanomethyl)naphthalen-2-yl methanesulfonate 3b. This compound was prepared following the general procedure for mesylation from 6-hidroxymethyl-2-naphtol **3a**[6] (530 mg, 3.05 mmol) by treatment with mesyl chloride (0.56 mL, 7.31 mmol) and triethylamine (2.53 mL, 18.28 mmol). The resulting material was subjected to the general procedure for the formation of nitriles. The reaction was stirred for 5 hours at 60 ºC and after the aqueous work up, the residue was purified by flash chromatography (hexane/ethyl acetate 6:4) to afford compound **3b** (430 mg, 1.65mmol, 54% two steps). ^{1}H NMR (400 MHz, Chloroform-*d*) δ 7.91 – 7.84 (m, 3H), 7.76 (d, *J* = 2.4 Hz, 1H), 7.44 (dt, *J* = 9.0, 1.8 Hz, 2H), 3.93 (s, 2H), 3.21 (s, 3H).^{13}C NMR (101 MHz, Chloroform-*d*) δ 147.5, 133.2, 132.2, 130.4, 129.3, 128.5, 127.1, 127.1, 122.1, 119.7, 117.9, 37.9, 24.1. HRMS (ESI+): calculated for [$C_{13}H_{11}NO_3S+H^+$]: [$M+H^+$] 262.0532, found: 262.0521.

[6] A. Samat, V. Lokshin, K. Chamontin, D. Levi, G. Pepe and R. Guglielmetti, *Tetrahedron* 2001, **57**, 7349.

2-(6-hydroxynaphthalen-2-yl)acetonitrile 3c. To a solution of compound **3b** (410 mg, 1.57 mmol) in THF/MeOH/H_2O 1:1:1 (7.8 mL) a 4 M NaOH solution (3.9 mL) was added and the reaction was stirred at room temperature for 20 h. HCl was then added (2% aq, 30 mL) and the product was extracted with CH_2Cl_2 (3 x 25 mL). The combined organic layer were dried over anhydrous Na_2SO_4, filtered and concentrated. The residue was purified by flash chromatography (hexane/ethyl acetate 7:3) to afford compound **3c** (122 mg, 0.67 mmol, 43%). ^{1}H NMR (400 MHz, Methanol-d_4) δ 7.69 (ddd, J = 6.1, 3.0, 1.1 Hz, 2H), 7.65 (d, J = 8.6 Hz, 1H), 7.32 (dd, J = 8.6, 1.9 Hz, 1H), 7.11 (s, 1H), 7.09 (d, J = 2.5 Hz, 1H), 3.95 (d, J = 1.0 Hz, 2H). ^{13}C NMR (101 MHz, Methanol-d_4) δ 156.9, 135.7, 130.3, 129.7, 128.2, 127.7, 127.0, 126.4, 120.0, 119.8, 109.8, 23.5. HRMS (ESI+): calculated for [$C_{12}H_9NO+H^+$]: [$M+H^+$] 184.0757, found: 184.0748.

2-(6-hydroxynaphthalen-2-yl)acetaldehyde 3. This compound was prepared from compound **3c** (50 mg, 0.27 mmol) in CH_2Cl_2 (2.73 mL) and DIBAL-H (1.08 mL, 1M in toluene) according to the general procedure for reduction of nitriles. Purification by flash chromatography (hexane/ethyl acetate 7:3) afforded **3** (16 mg, 0.09 mmol, 33%). ^{1}H NMR (400 MHz, $CDCl_3$) δ 9.81 (t, J = 2.4 Hz, 1H), 7.72 (d, J = 8.8 Hz, 1H), 7.69 (d, J = 8.4 Hz, 1H), 7.63 – 7.60 (m, 1H), 7.31 – 7.23 (m, 1H), 7.15 (d, J = 2.5 Hz, 1H), 7.12 (dd, J = 8.7, 2.6 Hz, 1H), 3.81 (d, J = 2.4 Hz, 2H). ^{13}C NMR (101 MHz, $CDCl_3$) δ 200.0, 154.0, 134.1, 129.8, 129.4, 128.8, 128.4, 127.5, 127.1, 118.7, 109.7, 50.9. HRMS (ESI+): calculated for [$C_{12}H_{10}O_2+H^+$]: [$M+H^+$] 187.0754, found: 187.0752.

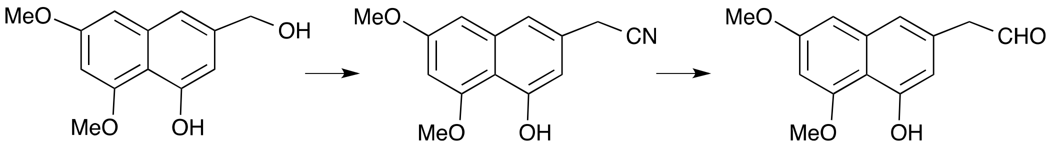

Scheme 3. Synthetic route for the preparation of 2-(6-hydroxynaphthalen-2-yl)acetaldehyde **4.**

2-(4-hydroxy-5,7-dimethoxynaphthalen-2-yl)acetonitrile 4b.

A solution of the readily available naphtoate **4a**[7] (400 mg, 1.71 mmol) in anhydrous THF (17 mL), cooled to 0 ºC and under inert atmosphere was treated with triethylamine (0.35 mL, 2.56 mmol) and triisopropylbenzenesulfonyl chloride (621 mg, 2.05 mmol). The mixture was stirred for 16 hours at

[7] a) F. M. Hauser, D. Sengupta and S. A. Corlett, *J. Org. Chem.* 1994, *59*, 1967; b) D. W. Cameron, G. I. Feutrill and L. J. H. Pannan, *Aut. J. Chem.*1980, **33**, 2531.

room temperature and then 24 hours at 50 ºC. It was poured onto water and extracted twice with EtOAc (2 x 30 mL). The combined organic layers were dried over anhydrous Na_2SO_4, filtered and concentrated. The residue was purified by flash chromatography (hexane/ethyl acetate 8:2) to afford a material containing an inseparable mixture of the expected triisopropylbenzenesulfonate derivative along with the corresponding chloride substitution product. The mixture was subjected to the general procedure for the formation of nitriles. The reaction was stirred for 24 hours at 50 ºC and after the aqueous work up, the residue was purified by flash chromatography (hexane/ethyl acetate 7:3) to afford compound **4b** (36 mg, 0.15 mmol, 9% two steps). ^{1}H NMR (400 MHz, $CDCl_3$) δ 9.18 (s, 1H), 7.18 (s, 1H), 6.70 (d, J = 2.3 Hz, 1H), 6.61 (d, J = 1.7 Hz, 1H), 6.47 (d, J = 2.2 Hz, 1H), 4.03 (s, 3H), 3.89 (s, 3H), 3.78 (s, 2H); ^{13}C NMR (126 MHz, $CDCl_3$) δ 158.6, 157.3, 155.5, 137.7, 130.1, 117.9, 116.9, 110.3, 108.2, 99.5, 98.3, 56.4, 55.6, 23.8. HRMS (ESI+): calculated for [$C_{14}H_{14}NO_3$+H^+]: [M+H^+] 244.09682, found: 244.09717.

2-(4-hydroxy-5,7-dimethoxynaphthalen-2-yl)acetaldehyde 4. Compound **4b** (30 mg, 0.12 mmol) was treated with DIBAL-H (0.62 mL, 1M in toluene) in CH_2Cl_2 (1.23 mL) according to the general procedure for reduction of nitriles. Compound **4** (11 mg, 0.04 mmol, 37%) was obtained after purification by flash chromatography (hexane/ethyl acetate 8:2). ^{1}H NMR (500 MHz, CD_3OD) δ 7.05 (s, 1H), 6.74 (d, J = 2.4 Hz, 1H), 6.57 (s, 1H), 6.50 (d, J = 2.3 Hz, 1H), 4.73 (t, J = 5.5 Hz, 1H), 4.02 (s, 3H), 3.86 (s, 3H), 2.91 (dd, J = 13.7, 5.4 Hz, 1H), 2.83 (dd, J = 13.7, 5.7 Hz, 1H); ^{13}C NMR (126 MHz, CD_3OD) δ 159.4, 158.6, 155.4, 139.0, 138.8, 119.6, 111.1, 110.6, 100.3, 100.2, 98.1, 56.7, 55.7, 44.6. HRMS (ESI+): calculated for [$C_{14}H_{15}O_4$+H^+]: [M+H^+] 247.09649, found 247.09655

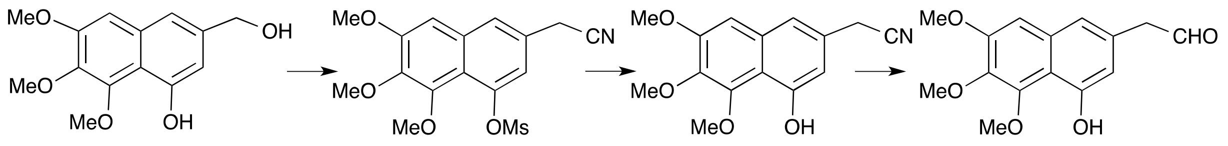

5a **5b** **5c** **5**

Scheme 4. Synthetic route for the preparation of 2-(4-hydroxy-5,6,7-trimethoxynaphthalen-2-yl)acetaldehyde **5.**

2-(4-methanosulphonyloxy-5,6,7-trimethoxynaphthalen-2-yl)acetonitrile 5b. 3-(Hydroxymethyl)-6,7,8-trimethoxynaphthalen-1-ol **5a**[8] (237 mg, 0.84 mmol) was treated with mesyl chloride (143 μL, 1.84 mmol) and triethylamine (412 μL, 2.94 mmol) according to the general procedure of mesylation. After usual work-up, the residue containing unpurified dimesylate was dissolved in dry DMF (5 mL) and subjected to the general procedure for the formation of nitriles by treatment with KCN (330 mg, 5.04 mmol). The reaction was stirred overnight at room temperature and after aqueous work up, and purification by flash chromatography (hexane/ethyl acetate 7:3) afforded compound **5b** (180 mg, 61% two steps). ^{1}H NMR (500 MHz, $CDCl_3$) δ 7.72 – 7.69 (m, 1H), 7.24 (d, *J* = 1.7, 1H), 7.02 (s, 1H), 4.00 (s, 3H), 3.99 (s, 3H), 3.97 (s, 3H), 3.89 (s, 2H), 3.17 (s, 3H); ^{13}C NMR (126 MHz, $CDCl_3$) δ 154.3, 147.5, 144.4, 144.2, 133.3, 127.0, 125.3, 118.7, 117.3, 117.0, 103.6, 62.3, 61.6, 56.1, 37.4, 23.5. HRMS (ESI+): calculated for [$C_{16}H_{18}NO_6S+H^+$]: [$M+H^+$] 352.08493, found: 352.08381.

2-(4-hydroxy-5,6,7-trimethoxynaphthalen-2-yl)acetonitrile 5c. A solution of the mesylate **5b** (120 mg, 0.34 mmol) in THF/MeOH/H_2O (1:1:1) (5 mL) was treated with aqueous NaOH (0.85 mL, 4M). The reaction was stirred at r.t. overnight. Then, it was neutralized with HCl (1M) and extracted with EtOAc (3 x 10 mL). The organic layers were washed with brine, dried over anhydrous Na_2SO_4 and concentrated. The residue was purified by flash chromatography (hexane/ethyl acetate 7:3) to afford nitrile **5c** (37 mg, 40%). ^{1}H NMR (400 MHz, $CDCl_3$) δ 9.53 (s, 1H), 7.17 (s, 1H), 6.90 (s, 1H), 6.62 (d, *J* = 1.7 Hz, 1H), 4.14 (s, 3H), 3.96 (s, 3H), 3.94 (s, 3H), 3.78 (s, 2H); ^{13}C NMR (101 MHz, $CDCl_3$) δ 154.6, 153.5, 148.3, 140.0, 132.6, 128.7, 117.9, 116.6, 112.2, 108.2, 103.4, 62.5, 61.3, 56.0, 23.8. HRMS (ESI+): calculated for [$C_{15}H_{16}NO_4+H^+$]: [$M+H^+$] 274.10738, found: 274.10774.

2-(4-hydroxy-5,6,7-trimethoxynaphthalen-2-yl)acetaldehyde 5. This compound was prepared from nitrile **5c** (23 mg, 0.08 mmol) following the general procedure for nitrile reduction with DIBAL-H (0.4 mL, 1M in toluene). Purification by flash chromatography (hexane/ethyl acetate 7:3) afforded **5** (9 mg, 45%). ^{1}H NMR (500 MHz, $CDCl_3$) δ 9.80 – 9.74 (t, *J* = 2.4 Hz, 1H), 9.49 (s, 1H), 7.03 (s, 1H), 6.88 (s, 1H), 6.62 (s, 1H), 4.15 (s, 3H), 3.96 (s, 3H), 3.94 (s, 3H), 3.70 (d, *J* = 2.4 Hz, 2H). ^{13}C NMR (101 MHz, $CDCl_3$) δ 199.7, 154.7, 153.6, 148.6, 140.1, 133.1, 131.1, 118.7, 112.3, 110.3, 103.5, 62.7, 61.6, 56.2, 50.9. HRMS (ESI+): calculated for [$C_{15}H_{17}O_5+H^+$]: [$M+H^+$] 277.10705, found: 277.10720.

---

[8] A. Samat, V. Lokshin, K. Chamontin, D. Levi, G. Pepe and R. Guglielmetti, *Tetrahedron* 2001, **57**, 7349.

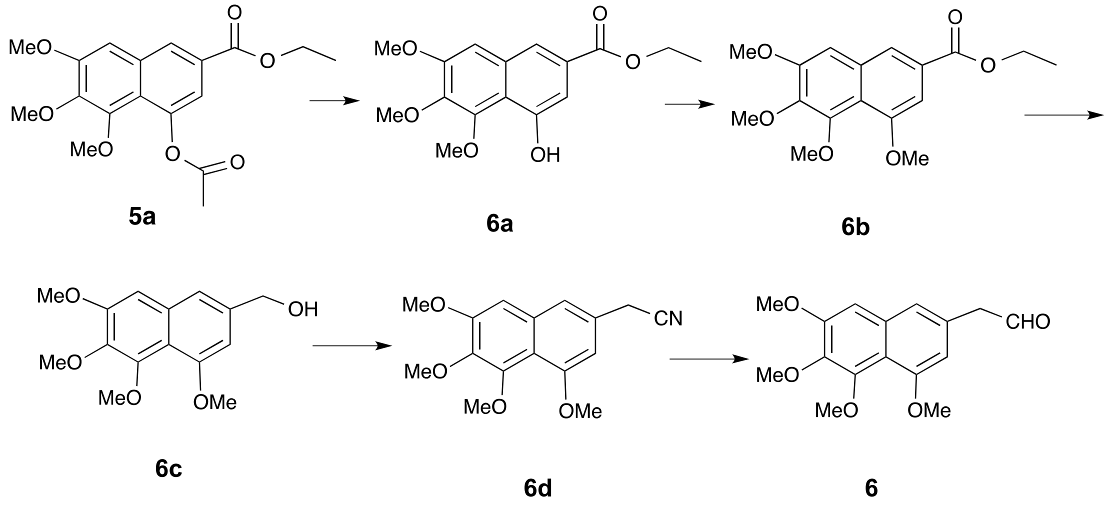

Scheme 5. Synthetic route for the preparation of 2-(4,5,6,7-tetramethoxynaphthalen-2-yl)acetaldehyde **6.**

Ethyl 4-hydroxy-5,6,7-trimethoxy-2-naphthoate 6a. Ethyl 4-acetoxy-5,6,7-trimethoxy-2-naphthoate[9] **5a** (1.5 g, 4.3 mmol) was dissolved in a mixture of EtOH/Et_3N (4:1, 10 mL) and heated at 80ºC for 10 h. After concentration, the residue was purified by flash chromatography (hexane/ethyl acetate 8:2) to yield compound **6a** (1.06 gr, 80%). ^{1}H NMR (500 MHz, $CDCl_3$) δ 9.47 (s, 1H), 7.95 (d, *J* = 1.6 Hz, 1H), 7.33 (d, *J* = 1.6 Hz, 1H), 7.03 (s, 1H), 4.40 (d, *J* = 7.1Hz, 2H), 4.15 (s, 3H), 3.98 (s, 3H), 3.97 (s, 3H), 1.42 (t, *J* = 7.1Hz, 3H); ^{13}C NMR (126 MHz, $CDCl_3$) δ 166.6, 153.8, 153.2, 148.0, 141.4, 131.6, 128.8, 120.5, 115.1, 107.9, 104.7, 62.4, 61.2, 61.0, 55.9, 14.3. HRMS (ESI+): calculated for [$C_{16}H_{19}O_6$+H^+]: [M+H^+] 307.11761; found: 307.11609.

Ethyl 4,5,6,7-tetramethoxy-2-naphthoate 6b. A solution of **6a** (977 mg, 3.5 mmol) in dry DMF (10 mL) was cooled to 0 °C and treated under argon atmosphere with NaH (60% dispersion in mineral oil, 431 mg, 10.5 mmol). After stirring at 0 °C for 30 min, methyl iodide (2.2 mL, 35 mmol) was added. The reaction mixture was then allowed to reach room temperature and stirred overnight. The reaction was diluted with water (10 mL) and extracted with diethyl ether (3 x 25 mL). The organic layers were dried over Na_2SO_4, filtered, concentrated *in vacuo*, and then purified by flash column chromatography (hexane/ethyl acetate 8:2) to yield compound **6b** (870 mg, 78%). ^{1}H NMR (500 MHz, $CDCl_3$) δ 8.05 (dd, *J* = 1.5, 0.6 Hz, 1H), 7.30 (d, *J* = 1.4 Hz, 1H), 7.05 (s, 1H), 4.42 (q, *J* = 7.1 Hz, 2H), 4.05 (s, 3H),

[9] H. Pati, R. LeBlanc and M. Lee, *Heterocycl. Commun.* 2003, **9**, 587.

3.99 (s, 3H), 3.97 (s, 3H), 3.92 (s, 3H), 1.44 (t, J = 7.1 Hz, 3H); ^{13}C NMR (126 MHz, $CDCl_3$) δ 166.9, 156.2, 153.4, 149.6, 144.5, 132.3, 127.6, 122.83, 118.6, 104.8, 103.1, 77.4, 62.3, 61.5, 61.2, 56.3, 56.0, 14.6. HRMS (ESI+): calculated for [$C_{17}H_{21}O_6$+H^+]: [M+H^+] 321.13326; found: 321.13335.

(4,5,6,7-tetramethoxynaphthalen-2-yl)metanol 6c. Lithium aluminium hydride (106 mg, 2.8 mmol) was added in one portion to a stirred solution of naphthoate **6b** (260 mg, 0.93 mmol) in THF (5 mL) at 0 °C under argon. The resulting suspension was stirred for 1 h. After cooling to 0 °C, Et_2O was added followed by the carefully portionwise addition of solid hydrated sodium sulfate until effervescence ceased. The solids were removed by filtration through Celite and the filter cake was washed with 9:1 CH_2Cl_2-MeOH (150 mL). The filtrate was dried (Na_2SO_4) and then purified by flash column chromatography (hexane/ethyl acetate 7:3) to yield derivative **6c** (212 mg, 82%). ^{1}H NMR (400 MHz, $CDCl_3$) δ 7.22 (d, J = 1.3 Hz, 1H), 6.90 (s, 1H), 6.74 (d, J = 1.3 Hz, 1H), 4.76 (s, 2H), 4.00 (s, 3H), 3.96 (s, 3H), 3.94 (s, 3H), 3.91 (s, 3H); ^{13}C NMR (101 MHz, $CDCl_3$) δ 156.6, 153.3, 149.7, 142.6, 138.6, 133.3, 117.5, 115.7, 103.5, 103.1, 65.8, 62.3, 61.6, 56.2, 56.0. HRMS (ESI+): calculated for [$C_{15}H_{19}O_5$+H^+]: [M+H^+] 279.1227; found: 279.1223.

2-(4,5,6,7-tetramethoxynaphthalen-2-yl)acetaldehyde 6. This compound was prepared following the general procedure for mesylation from compound **6c** (170 mg, 0.60 mmol) by treatment with mesyl chloride (70 μL, 0.9 mmol) and triethylamine (252 μL, 1.8 mmmol). After usual work-up, the crude residue was dissolved in dry DMF (5 mL) and subjected to the general procedure for the formation of nitriles by treatment with KCN (117 mg, 1.8 mmol). The reaction was stirred overnight at room temperature and after aqueous work up, and purification by flash chromatography (hexane/ethyl acetate 7:3) afforded compound **6d**, which was treated with DIBAL-H (0.12 mL, 1M in toluene) according to the general procedure for reduction of nitriles. Purification by flash chromatography (hexane/ethyl acetate 7:3) afforded **6** (35 mg, 20%). ^{1}H NMR (500 MHz, CD_3OD) δ 7.18 (s, 1H), 7.02 (s, 1H), 6.75 (s, 1H), 4.76 (t, J = 5.5 Hz, 1H), 3.95 (s, 3H), 3.94 (s, 3H), 3.89 (s, 3H), 3.85 (s, 3H), 2.97 (dd, J = 13.8, 5.3 Hz, 1H), 2.89 (dd, J = 13.8, 5.7 Hz, 1H); ^{13}C NMR (126 MHz, CD_3OD) δ 156.9, 154.1, 150.2, 143.0, 136.4, 134.8, 121.3, 115.7, 107.7, 104.6, 100.4, 62.6, 61.7, 56.4, 56.2, 44.7. HRMS (ESI+): calculated for [$C_{16}H_{19}O_5$+H^+]: [M+H^+] 291.1227; found: 291.12304.

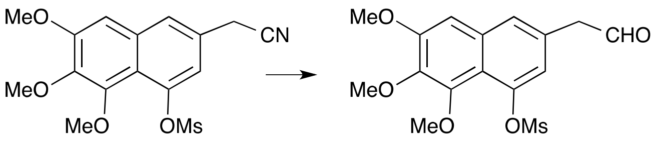

5b **7**

Scheme 6. Synthetic route for the preparation of 2-(4,5,6,7-tetramethoxynaphthalen-2-yl)acetaldehyde **7.**

2-(4,5,6,7-tetramethoxynaphthalen-2-yl)acetaldehyde 7. This compound was prepared from compound **5b** (22 mg, 0.06 mmol) in CH_2Cl_2 (1.2 mL) and DIBAL-H (0.12 mL, 1M in toluene) according to the general procedure for reduction of nitriles. Purification by flash chromatography (hexane/ethyl acetate 6:4) afforded **7** (4 mg, 15%). ^{1}H NMR (500 MHz, CD_3OD) δ 7.61 (s, 1H), 7.29 (s, 1H), 7.16 (s, 1H), 4.75 (t, *J* = 5.4 Hz, 1H), 3.97 (s, 3H), 3.93 (s, 3H), 3.92 (s, 3H), 3.20 (s, 3H), 3.01 (dd, *J* = 13.9, 5.4 Hz, 1H), 2.95 (dd, *J* = 13.9, 5.6 Hz, 1H); ^{13}C NMR (126 MHz, CD_3OD) δ 154.9, 148.5, 144.9, 144.4, 136.1, 134.7, 128.1, 121.8, 117.4, 104.6, 99.9, 62.5, 61.7, 56.4, 44.0, 37.4. HRMS (ESI+): calculated for [$C_{16}H_{18}O_7S+H^+$]: [$M+H^+$] 355.0846, found: 355.08465.

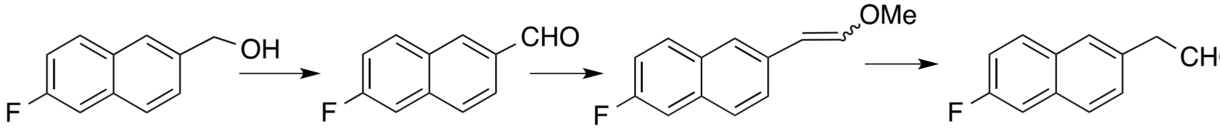

8a **8b** **8c** **8**

Scheme 7. Synthetic route for the preparation of 2-(6-fluoronaphthalen-2-yl)acetaldehyde **8.**

6-fluoro-2-naphthaldehyde 8b. This compound was prepared following the general experimental procedure for Dess-Martin oxidation from (6-fluoronaphthalen-2-yl)methanol[10] (400 mg, 2.27 mmol) in CH_2Cl_2/THF 1:1 (13.6 mL). Purification by flash chromatography (hexane/ethyl acetate 9:1) afforded compound **8b** (360 mg, 76%). ^{1}H NMR (400 MHz, $CDCl_3$) δ 10.14 (s, 1H), 8.33 (dd, *J* = 1.5, 0.8 Hz, 1H, H1), 8.04 – 7.95 (m, 2H, H3, H8), 7.88 (d, *J* = 8.5 Hz, 1H, H4), 7.52 (dd, *J* = 9.4, 2.7 Hz, 1H, H5), 7.37 (ddd, *J* = 9.0, 8.3, 2.6 Hz, 1H, H7). ^{13}C NMR (101 MHz, $CDCl_3$) δ 192.2, 162.8 (d, *J* = 251.4 Hz), 137.9 (d, *J* = 9.8 Hz), 134.6 , 133.9 (d, *J* = 2.6 Hz), 132.5 (d, *J* = 9.5 Hz), 129.9 , 128.8 (d, *J* = 5.4 Hz), 124.3 , 117.9 (d, *J* = 25.4 Hz), 111.9 (d, *J* = 20.8 Hz). HRMS (ESI+): calculated for [$C_{11}H_7FO+H^+$]: [$M+H^+$] 175.0554, found: 175.0559

[10] J.-H. Chun and V. W. Pikea, *Org. Biomol. Chem.* 2013, **11**, 6300.

2-fluoro-6-(2-methoxyvinyl)naphthalene 8c. Compound **8b** (360 mg, 2.07 mmol), was treated with (methoxymethyl)triphenylphosphonium chloride (2837 mg, 8.28 mmol) and BuLi (1.6 M in hexane, 5.1 mL, 8.19 mmol) according to the general procedure. The residue was purified by flash chromatography (hexane/ethyl acetate 9:1) to afford compound **8c** (372 mg, 1.84 mmol, 89%) as a 1:1 mixture of Z/E isomers ^{1}H NMR (400 MHz, $CDCl_3$) δ 7.93 (s, 1H), 7.73 – 7.55 (m, 5H), 7.49 (s, 1H), 7.39 – 7.35 (m, 1H), 7.32 – 7.28 (m, 2H, H5 de Z y E), 7.18 – 7.07 (m, 2H, H7 both isomers), 7.09 (d, J = 12.9 Hz, 1H, E), 6.14 (d, J = 7.0 Hz, 1H, Z), 5.88 (d, J = 12.9 Hz, 1H, E), 5.28 (d, J = 7.0 Hz, 1H, Z), 3.75 (s, 3H), 3.65 (s, 3H). ^{13}C NMR (101 MHz, $CDCl_3$, selected peaks) δ 160.4 (d, J = 244.9 Hz), 160.2 (d, J = 244.6 Hz), 149.4, 148.5, 116.5 (d, J = 25.2 Hz), 116.2 (d, J = 25.4 Hz), 110.9 (d, J = 18.7 Hz), 110.7 (d, J = 18.8 Hz), 105.5, 105.1, 60.9, 56.7. HRMS (ESI+): calculated for [$C_{13}H_{11}FO+H^+$]: [$M+H^+$] 203.0867, found: 203.0868.

2-(6-fluoronaphthalen-2-yl)acetaldehyde 8. Compound **8c** (100 mg, 0.50 mmol) was subjected to the general procedure for the hydrolysis of the vinyl ethers by treatment with TFA (0.2 mL) in CH_2Cl_2 (5 mL) at rt for 20 minutes. The crude material was purified by flash chromatography (hexane/ethyl acetate 95:5) to give compound **3** (30 mg, 0.16 mmol, 32%). ^{1}H NMR (400 MHz, $CDCl_3$) δ 9.83 (t, J = 2.3 Hz, 1H), 7.80 (m,, 2H, H4 y H8), 7.71 – 7.66 (m, 1H, H1), 7.45 (dd, J = 9.7, 2.3 Hz, 1H, H5), 7.35 (dd, J = 8.9, 1.5 Hz, 1H, H4), 7.31 – 7.25 (m, 1H, H7), 3.85 (d, J = 2.3 Hz, 2H). ^{13}C NMR (101 MHz, $CDCl_3$) δ 199.3 (CHO), 160.8 (d, J = 246.2 Hz, C6), 133.4 (d, J = 9.3 Hz), 130.7 , 130.1 (d, J = 9.2 Hz), 128.7 (d, J = 2.8 Hz), 128.6 , 128.2 (d, J = 5.7 Hz), 117.0 (d, J = 25.2 Hz), 111.0 (d, J = 20.5 Hz), 50.7 .

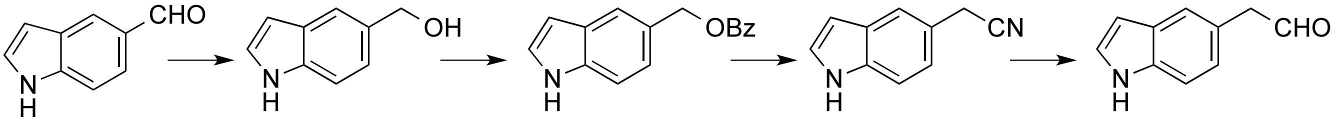

9a **9b** **9c** **9d** **9**

Scheme 8. Synthetic route for the preparation of 2-(1*H*-indol-5-yl)acetaldehyde **9.**

(1*H*-indol-5-yl)methanol 9b. Lithium aluminium hydride (1.04 g, 27.6 mmol) was added in one portion to a stirred solution of indole-5-carboxaldehyde **9a** (1.0 g, 6.90 mmol) in THF (5 mL) at 0 °C under argon. The resulting suspension was stirred for 1 h. After cooling to 0 °C, Et_2O was added followed by the carefully portionwise addition of solid hydrated sodium sulfate until effervescence ceased. The solids were removed by filtration through Celite and the filter cake was washed with 9:1 CH_2Cl_2-MeOH

(150 mL). The filtrate was dried (Na_2SO_4) and then purified by flash column chromatography (hexane/ethyl acetate 7:3) to yield derivative **9b** (1.0 g, 6.90 mmol, quantitative). ^{1}H NMR (400 MHz, $CDCl_3$) δ 8.33 (s, 1H, NH), 7.63 (dt, *J* = 1.6, 0.8 Hz, 1H), 7.36 (dt, *J* = 8.3, 0.8 Hz, 1H), 7.24 – 7.19 (m, 2H), 6.55 (ddd, *J* = 3.1, 2.0, 0.9 Hz, 1H), 4.76 (s, 2H); ^{13}C NMR (101 MHz, $CDCl_3$) δ 135.7, 132.6, 128.1, 125.1, 122.2, 119.9, 111.5, 102.8, 66.5. HRMS (ESI+): calculated for [$C_9H_{10}NO+H^+$]: [$M+H^+$] 148.0757, found: 148.0755.

(1*H*-indol-5-yl)methyl benzoate 9c. Benzoyl chloride (0.48mL, 4.14mmol) was added under inert atmosphere and at 0 ºC to a solution of **9b** (500 mg, 3.45 mmol) in anhydrous THF (34 mL) containing triethylamine (1.43 mL, 10.34 mmol). The mixture was stirred at room temperature overnight and then it was poured onto a saturated $NaHCO_3$ solution. The product was extracted twice with CH_2Cl_2. The combined organic layers were washed with water, dried over anhydrous Na_2SO_4, filtered and concentrated to yield compound **9c** (845 mg, quantitative). ^{1}H NMR (400 MHz, $CDCl_3$) δ 8.28 (s, 1H), 8.12 – 8.06 (m, 2H), 7.76 (dt, *J* = 1.5, 0.8 Hz, 1H), 7.57 – 7.52 (m, 1H), 7.45 – 7.39 (m, 3H), 7.31 (dd, *J* = 8.4, 1.7 Hz, 1H), 7.23 (dd, *J* = 3.2, 2.4 Hz, 1H), 6.58 (ddd, *J* = 3.1, 2.0, 0.9 Hz, 1H), 5.48 (s, 2H); ^{13}C NMR (101 MHz, $CDCl_3$) δ 166.9, 135.9, 133.1, 130.7, 129.9, 128.9, 128.5, 127.6, 125.1, 123.2, 121.6, 111.4, 103.0, 68.1. HRMS (ESI+): calculated for [$C_{16}H_{14}NO_2+H^+$]: [$M+H^+$] 252.1019, found: 252.1009.

2-(1*H*-indol-5-yl)acetonitrile 9d. Compound **9c** (845mg, 3.45 mmol) was dissolved in dry DMF (5 mL) and subjected to the general procedure for the formation of nitriles by treatment with KCN (1.19 g mg, 17.2 mmol). The reaction was heated at 105 ºC for 5 h. Aqueous work up and purification by flash chromatography (hexane/ethyl acetate 7:3) afforded compound **9d** (430 mg, 80%). ^{1}H NMR (400 MHz, $CDCl_3$) δ 8.47 (s, 1H), 7.59 (m, 1H), 7.38 (dt, *J* = 8.4, 0.9 Hz, 1H), 7.24 (dd, *J* = 3.2, 2.4 Hz, 1H), 7.10 (dd, *J* = 8.4, 1.9, 1H), 6.53 (ddd, *J* = 3.1, 2.0, 0.9 Hz, 1H), 3.83 (s, 2H); ^{13}C NMR (101 MHz, $CDCl_3$) δ 135.4, 128.4, 125.5, 121.8, 121.1, 120.2, 119.0, 111.8, 102.5, 23.8. HRMS (ESI+): calculated for [$C_{16}H_{14}NO_2+H^+$]: [$M+H^+$] 157.0687, found: 157.0754.

2-(1*H*-indol-5-yl)acetaldehyde 9. Compound **9d** (150 mg, 0.96 mmol) was treated with DIBAL-H (1.92 mL, 1M in toluene) according to the general procedure for reduction of nitriles. The crude material was purified by flash chromatography (hexane/ethyl acetate 8:2) to give compound **9** (77 mg, 50%) ^{1}H NMR (400 MHz, $CDCl_3$) δ 9.78 (t, *J* = 2.6 Hz, 1H), 8.23 (s, 1H), 7.50 (dt, *J* = 1.7, 0.8 Hz, 1H), 7.39 (dt, *J* = 8.3, 0.8 Hz, 1H), 7.23 (dd, *J* = 3.3, 2.3 Hz, 1H), 7.03 (dd, *J* = 8.3, 1.7 Hz, 1H), 6.54 (ddd, *J*

= 3.1, 2.0, 0.9 Hz, 1H), 3.77 (d, J = 2.6 Hz, 2H). ^{13}C NMR (101 MHz, $CDCl_3$) δ 200.6, 135.2, 128.6, 125.1, 123.7, 123.0, 121.8, 111.7, 102.6, 50.9. HRMS (ESI+): calculated for [$C_{10}H_{10}NO+H^+$]: [$M+H^+$] 160.0757, found: 160.0759.

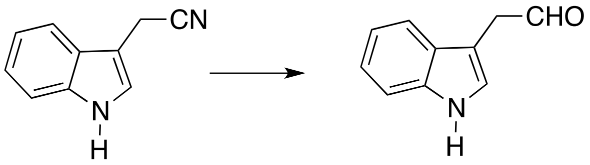

10a **10**

Scheme 9. Synthetic route for the preparation of 2-(1H-indol-3-yl)acetaldehyde **10**.

2-(1H-indol-3-yl)acetaldehyde 10. This compound was prepared according to the general procedure for reduction of nitriles from 3-indoleacetonitrile **10a** (150 mg, 0.96 mmol) in CH_2Cl_2 (9.6 mL) using DIBAL-H (2.4 mL, 1M in toluene). Purification by flash chromatography (hexane/ethyl acetate 8.2) afforded **10** (50 mg, 0.31 mmol, 33% yield). ^{1}H NMR (400 MHz, $CDCl_3$) δ 9.77 (t, J = 2.5 Hz, 1H), 8.20 (s, 1H) 7.56 (dt, J = 7.9, 1.0 Hz, 1H), 7.40 (dt, J = 8.2, 0.9 Hz, 1H), 7.24 (ddd, J = 8.2, 7.0, 1.1 Hz, 1H), 7.19 – 7.14 (m, 2H), 3.82 (dd, J = 2.5, 0.9 Hz, 2H). ^{13}CNMR (101 MHz, $CDCl_3$) δ 199.8, 123.5, 123.4, 122.7, 120.1, 120.1, 118.6, 111.5, 111.4, 40.5. HRMS (ESI+): calculated for [$C_{10}H_9NO+H^+$]: [$M+H^+$] 160.0757, found: 160.0760.

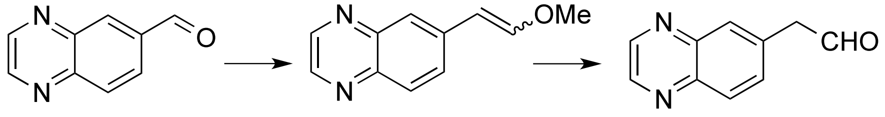

11a **11b** **11**

Scheme 10. Synthetic route for the preparation of 2-(Quinoxalin-6-yl)acetaldehyde **11**.

6-(2-Methoxyvinyl)quinoxaline 11b. This compound was prepared following the general procedure for the Wittig reaction from compound **11a** (50 mg, 0.32 mmol), (methoxymethyl)triphenylphosphonium chloride (434 mg, 1.26 mmol) and BuLi (1.6 M in hexane, 2.1 mL, 1.25 mmol). The residue was purified by flash chromatography (hexane/ethyl acetate 7:3) to afford compound **11b** (35 mg, 59%) as a 1:1 mixture of Z/E isomers ^{1}H NMR (400 MHz, $CDCl_3$) δ 8.78 (d, J = 1.9 Hz, 1H), 8.76 (d, J = 1.9 Hz, 1H), 8.73 (d, J = 1.9 Hz, 1H), 8.71 (d, J = 1.9 Hz, 1H), 8.30 (s, 1H), 7.99 – 7.95 (m, 3H), 7.82 (d, J = 2.0 Hz, 1H), 7.71 (dd, J = 8.8, 2.0 Hz, 1H), 7.29 (d, J = 13.0 Hz, 1H, E), 6.35 (d, J = 6.9 Hz, 1H, Z), 6.00 (d, J = 13.0 Hz, 1H,E), 5.43 (d, J = 7.0 Hz, 1H, Z), 3.88 (s, 3H), 3.77 (s, 4H). ^{13}C NMR (101 MHz,

$CDCl_3$) δ 151.6 (E), 150.9 (Z), 145.5, 145.3, 144.2, 144.0, 143.9, 143.7, 142.2, 141.9, 139.1, 138.4, 131.8, 129.8, 129.1, 128.0, 127.5, 124.3, 104.9 (Z), 104.6 (E), 61.5, 57.2. HRMS (ESI+): calculated for [$C_{11}H_{11}N_2O+H^+$]: [$M+H^+$] 187.0866, found: 187.0872.

2-(Quinoxalin-6-yl)acetaldehyde 11. Compound **11b** (26 mg, 0.14 mmol) was subjected to the general procedure for the hydrolysis of the vinyl ethers by treatment with TFA (0.2 mL) in CH_2Cl_2 (2 mL) at rt for 4 h. The crude material was purified by flash chromatography (hexane/ethyl acetate 7:3) to give compound **11** (15 mg, 64%). ^{1}H NMR (400 MHz, $CDCl_3$) δ 9.88 (t, *J* = 1.9 Hz, 1H), 8.87 – 8.82 (m, 2H), 8.11 (d, *J* = 8.6 Hz, 1H), 7.97 (dd, *J* = 1.9, 0.8 Hz, 1H), 7.62 (dd, *J* = 8.6, 2.0 Hz, 1H), 3.98 (d, *J* = 1.9 Hz, 2H). ^{13}C NMR (101 MHz, $CDCl_3$) δ 198.4, 145.6, 145.3, 143.2, 142.4, 134.6, 132.0, 130.3, 130.2, 50.6. HRMS (ESI+): calculated for [$C_{10}H_9N_2O+H^+$]: [$M+H^+$] 173.0709, found: 173.0712.

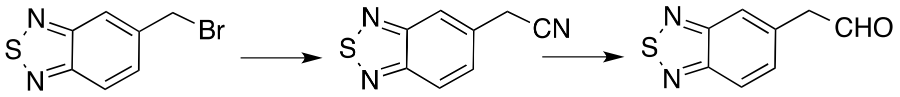

12a **12b** **12**

Scheme 11. Synthetic route for the preparation of 2-(benzo[*c*][1,2,5]thiadiazol-5-yl)acetaldehyde **12.**

2-(benzo[*c*][1,2,5]thiadiazol-5-yl)acetonitrile 12b. To a solution of 5-bromomethyl-benzo[1,2,5]thiadiazole **12a** (250 mg, 1.09 mmol) in EtOH/H_2O 2:1 (3 mL) was added KCN (85 mg, 1.35 mmol). The reaction was stirred at 50 ºC for 3 hours and then cooled to room temperature. The mixture was extracted twice with EtOAc and the organic phases were washed with brine, dried over anhydrous Na_2SO_4 and concentrated. The residue was purified by flash chromatography (hexane/ethyl acetate 85:15) to afford **12b**[11] (110 mg, 58%).

2-(benzo[*c*][1,2,5]thiadiazol-5-yl)acetaldehyde 12. Compound **12b** (50 mg, 0.29 mmol) was subjected to the general procedure for the reduction of nitriles. Purification of the residue by flash chromatography (hexane/ethyl acetate 8:2) afforded pure compound **12** (14 mg, 26%). ^{1}H NMR (500 MHz, Chloroform-*d*) δ 9.88 (t, *J* = 1.9 Hz, 1H), 8.01 (dd, *J* = 9.0, 0.8 Hz, 1H), 7.88 (dq, *J* = 1.7, 0.8 Hz, 1H), 7.44 (dd, *J* = 9.0, 1.7 Hz, 1H), 3.93 (dd, *J* = 1.9, 0.8 Hz, 2H). ^{13}C NMR (126 MHz, Chloroform-*d*) δ 198.1, 155.1, 154.2, 134.0, 131.6, 122.0, 122.0, 50.6. HRMS (ESI+): calculated for [$C_8H_6N_2OS+H^+$]: [$M+H^+$] 179.0274, found: 179.0266.

[11] V. G. Pesin *et al.*, *J. Gen. Chem. USSR* (Engl. Transl.), 1964, **34**, 1272.

6. Preparation and Characterization of disaccharides

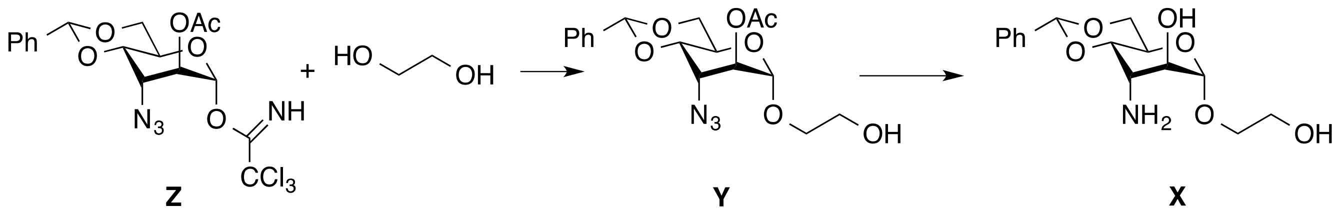

Z **Y** **X**

(2-Hydroxyethyl) 3-amino-4,6-*O*-benzylidene-3-deoxy α-D-altropyranoside (**X, Ref**): The compound was prepared following the general procedure of glycosylation from donor **Z** (0.266 mmol, 127 mg) and ethylene glycol (0.30 mL, 5.3 mmol) but at -20ºC for 1.5 h. The crude material was purified by column chromatography using hexane/EtOAc (6:4) to give glycosylated compound **Y** (13 mg, 13%) as a colorless oil. ^{1}H NMR (500 MHz, $CDCl_3$) δ 7.52 – 7.46 (m, 2H, Ph), 7.41 – 7.34 (m, 3H, Ph), 5.62 (s, 1H, H7), 5.02 (dd, *J* = 2.5, 1.0 Hz, 1H, H2), 4.75 (d, *J* = 1.0 Hz, 1H, H1), 4.37 – 4.29 (m, 2H), 4.11 – 4.04 (m, 2H), 3.86 – 3.74 (m, 4H), 3.61 (m, 1H); ^{13}C NMR (126 MHz, $CDCl_3$) δ 169.6 (CO), 137.2, 129.6, 128.7, 126.5, 102.7 (C7), 98.0 (C1), 76.2, 70.6 (C2), 70.0 (CH_2), 69.3 (C6), 62.0 (CH_2), 59.3, 58.0, 21.3 (CH_3); HRMS (ESI) m/z calcd for $C_{17}H_{25}N_4O_7$ [$M+NH_4^+$]: 397.1718, found: 397.1722.

Compound **Y** (13 mg, 0.035 mmol) was subjected to the general procedure for reduction of the azide moiety by treatment with PMe_3 (53 μL, 0.053 mmol) and 0.1 M NaOH (71 μL) at rt for 6h. The crude material was purified by column chromatography on silica gel using CH_2Cl_2/MeOH (9:1) to give the desired product **X** (9 mg, 90%): ^{1}H NMR (500 MHz, D_2O) δ 7.57 – 7.52 (m, 2H, Ph), 7.50 – 7.43 (m, 3H, Ph), 5.84 (s, 1H, H7′), 4.87 (s, 1H, H1), 4.33 (dd, *J* = 10.2, 4.9 Hz, 1H, H6a), 4.22 (dd, *J* = 9.9, 4.9 Hz, 1H, H5), 4.18 (dd, *J* = 10.1, 4.0 Hz, 1H, H4), 4.04 (dd, *J* = 2.8, 1.2 Hz, 1H, H2), 3.93 (t, *J* = 10.0 Hz, 1H, H6b), 3.88 (ddd, *J* = 11.0, 4.9, 4.0 Hz, 1H, OCH_2CH_2OH), 3.81–3.76 (m, 2H, -OCH_2CH_2OH), 3.63– 3.57 (m, 1H, -OCH_2CH_2OH), 3.43 – 3.39 (m, 1H, H3); ^{13}C NMR (126 MHz, D_2O) δ 137.5, 131.2, 130.0, 127.6, 103.5 (C7), 101.5 (C1), 76.2 (C4), 70.6 (C2), 70.5 (-OCH_2CH_2OH), 69.6 (C6), 61.7 (-OCH_2CH_2OH), 59.4 (C5), 52.0 (C3); HRMS (ESI) m/z calcd for $C_{15}H_{22}NO_6$ [$M+H^+$]: 312.1442, found: 312.1444.

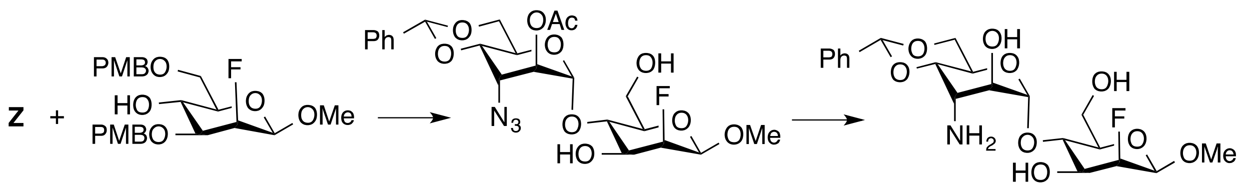

A1 **A2** **A**

Methyl 4-*O*-(3-amino-4,6-*O*-benzylidene-3-deoxy-α-D-altropyranosyl)-2-deoxy-2-fluoro-β-D-mannopyranoside (**A**). The compound was prepared from donor **Z** (0.266 mmol, 262 mg) and methyl 2-deoxy-2-fluoro-3,6-di-O-*p*-methoxybenzyl-β-D-mannopyranoside **A1**[12] (0.30 mL, 5.3 mmol) following the general procedure of glycosylation but in the presence of 0.7 equiv. of TMSOTf and at -50ºC for 1.5 h. The crude material was purified by column chromatography using hexane/EtOAc (6:4) to give glycosylated compound **A2** in which cleavage of the PMB-ethers was also observed (18 mg, 34%). ^{1}H NMR (500 MHz, $CDCl_3$) δ 7.53 – 7.35 (m, 5H, Ph), 5.62 (s, 1H, H-7'), 5.26 (s, 1H, H1'), 5.04 (dd, *J* = 2.7, 0.8 Hz, 1H, H2'), 4.73 (dd, *J* = 51.1, 2.6 Hz, 1H, H2), 4.47 (d, *J* = 19.0 Hz, 1H, H1), 4.40 – 4.27 (m, 2H), 4.15 – 4.09 (m, 2H), 4.06 (dd, *J* = 9.4, 3.6 Hz, 1H), 4.02 – 3.95 (m, 2H), 3.90 (dd, *J* = 12.1, 4.1 Hz, 2H), 3.86 – 3.80 (m, 1H), 3.78 (t, *J* = 10.1 Hz, 1H, H4), 3.59 (s, 3H, -OCH_3), 3.41 (dt, *J* = 9.5, 3.4 Hz, 1H), 2.15 (s, 3H, -OAc).; ^{13}C NMR (126 MHz, $CDCl_3$) δ 137.5, 129.7, 127.0, 103.2 (C-1'), 102.9, 99.9 (C-1), 91.5 (d, *J*=186.0, C-2), 76.4 (C-4'), 76.3 (C-4), 75.2 (C-5), 72.6 (C-3), 71.0 (C-2′), 69.0 (C-6'), 61.5 (C-6), 59.5 (C-5'), 57.9 (-OCH_3), 51.4 (C-3')

Compound **A2** (11 mg, 0.023 mmol) was treated with PMe_3 (40 µL, 0.04 mmol) and 0.1 M NaOH (60 µL) according to the general procedure of reduction of the azido moiety. The material was purified by column chromatography on silica gel using CH_2Cl_2/MeOH (93:7) to give the desired product **A** (7 mg, 52% overall yield): ^{1}H NMR (500 MHz, D_2O) δ 7.57 (dd, *J* = 6.8, 2.9 Hz, 2H, Ph), 7.49 (dd, *J* = 5.2, 1.8 Hz, 3H, Ph), 5.86 (s, 1H, H-7'), 5.14 (s, 1H, H1'), 4.83 (dd, *J*=50, 2.5 Hz, 1H, 2H) 4.73 (d, *J* = 19.5 Hz, 1H, H1), 4.35 (dd, *J* = 10.3, 5.0 Hz, 1H, H6'), 4.30 – 4.22 (m, 1H, H5'), 4.19 – 4.13 (m, 1H, H4'), 4.12 – 4.09 (m, 1H, H3'), 4.02 – 9.92 (m, 3H, H6, H6', H3) 3.85 (dd, *J* = 12.1, 5.4 Hz,1H, H6), 3.79 (t, *J* = 9.6 Hz, 1H, H4), 3.60 (s, 4H, -OCH_3, H5), 3.36 – 3.29 (m, 1H, H2); ^{13}C NMR (126 MHz, D_2O) δ δ 137.5, 129.0, 126.3, 102.3, 101.8 (C-1'), 99.2 (C-1), 78.9 (C-4), 73.3 (C-4'), 73.2 (C-3), 71.1 (C-2), 69.9 (C-5), 68.2 (C-6'), 68.1 (C-2'), 60.8 (C-6), 59.2 (C-5'), 55.1 (-OCH_3), 50.2 (C-3')

[12] Prepared from methyl 3-*O*-p-methoxybenzyl-4,6-*p*-methoxybenzylidene β-D-glucopyranoside as described for the benzyl analogue in T. Haradahira, M. Maeda, H. Omae, Y. Yano and M. Kojima, *Chem. Pharm. Bull.* 1984, **32**, 4758.

HRMS (ESI) m/z calcd for $C_{20}H_{29}FNO_9$ [M+H$^+$]: 446.1821, found: 446.1826.

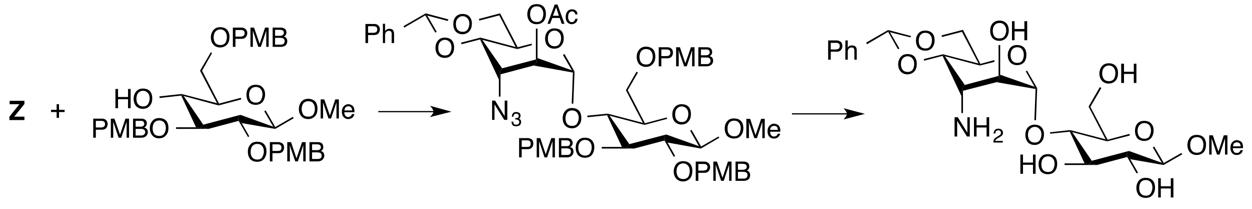

B1 **B2** **B**

Methyl 4-*O*-(3-amino-4,6-*O*-benzylidene-3-deoxy-α-D-altropyranosyl)- β-D-glucopyranoside (B). The compound was prepared following the general procedure of glycosylation from donor **Z** (164 mg, 0.34 mmol) and acceptor **B1**[13] (63 mg, 0.11 mmol mmol) at -65ºC for 25 min. The crude material was purified by column chromatography on silica gel using hexane/EtOAc (gradient 8:2 then 6:4) to give glycosylated compound **B2** (24 mg, 25%): ^{1}H NMR (500 MHz, $CDCl_3$): 7.36-7.52 (m, 5H, Ph), 7.11-7.33 (m, 6H, PMB), 6.80-6.88 (m, 6H, PMB), 5.58 (s, 1H, H7'), 5.26 (s, 1H, H1'), 4.94 (d, J=10.9 Hz, 1H, PMB), 4.93 (d, J=2.9 Hz, 1H, H2'), 4.85 (d, J=10.5 Hz, 1H, PMB), 4.64 (d, J=12.0 Hz, 1H, PMB), 4.61 (d, J=10.5 Hz, 1H, PMB), 4.56 (d, J=12.0 Hz, 1H, PMB), 4.55 (d, J=10.9 Hz, 1H, PMB), 4.32 (d, J=7.8 Hz, 1H, H1), 4.22-4.27 (m, 1H, H5'), 4.17 (dd, J=10.3, 5.5 Hz, 1H, H6a'), 4.03 (dd, J=3.5, 2.9 Hz, 1H, H3'), 3.95 (dd, J=9.4, 3.5 Hz, 1H, H4'), 3.86 (dd, J=9.8, 9.0 Hz, 1H, H4), 3.80 (s, 3H, PMB), 3.82-3.79 (m, 2H, H6a, H6b), 3.79 (s, 3H, PMB), 3.74 (s, 3H, PMB), 3.69 (dd, J=10.3, 3.1 Hz, 1H, H6b'), 3.65 (dd, J=9.0, 7.4 Hz, 1H, H3), 3.60 (s, 3H, $-OCH_3$), 3.51(ddd, J=9.8, 3.3, 3.3 Hz, 1H, H5), 3.43 (dd, J=9.0, 7.8 Hz, 1H, H2), 1.94 (s, 3H, AcO); ^{13}C RMN (126 MHz, $CDCl_3$); 168.9 (CO), 159.2, 159.0, 158.8, 137.0, 130.5, 130.4, 130.3, 129.8, 129.5, 129.2, 128.7, 128.3, 126.1, 113.7, 113.7, 113.6, 104.6 (C1), 102.0 (C7´), 97.7 (C1'), 84.4 (C3), 82.1 (C2), 75.7 (C4'), 74.4 (PMB), 74.1 (PMB), 74.1 (C5), 73.3, 73.0 (C4), 70.3 (C2'), 68.8 (C6'), 68.5 (C6), 59.3 (C5'), 57.3 (CH_3), 57.0 (C3'), 55.2 (CH_3,), 55.1 (CH_3), 20.7 (CH_3); MS (API-ES positive mode): 889 $(M+NH_4)^+$; HRMS (ESI) m/z calcd for $C_{46}H_{57}N_4O_{14}$ $[M+NH_4]^+$: 889.3866, found: 889.3892.

Compound **B2** (24 mg, 0.03 mmol) was subjected to the general procedure for deprotection of the PMB protecting groups (method A) over a course of 6 h. The crude material was purified by column chromatography using CH_2Cl_2/MeOH (95:5) and the resulting residue (12 mg, 0.027 mmol) was treated with PMe_3 (40 μL, 0.04 mmol) and 0.1 M NaOH (60 μL) according to the general procedure of reduction of the azido moiety. The material was purified by column chromatography on silica gel using

[13] Prepared from methyl β-D-glucopyranoside as described for methyl α-D-glucopyranoside in C. Grandjean and G. Lukacs, *J. Carbohydr. Chem.* 1996, **15**, 831.

CH_2Cl_2/MeOH/Dioxane/NH_4OH mixture (7:2:1.5:1) to give the desired product **B** (7 mg, 52% overall yield): ^{1}H NMR (500 MHz, D_2O) δ 7.59 – 7.52 (m, 2H, Ph), 7.52 – 7.44 (m, 3H, Ph), 5.86 (s, 1H, H-7′), 5.17 (s, 1H, H1'), 4.38 (d, *J* = 7.9 Hz, 1H, H1), 4.33 (dd, *J* = 10.4, 4.9 Hz, 1H, H6a'), 4.25 (td, *J* = 10.0, 4.9 Hz, 1H, H5′), 4.16 (dd, *J* = 10.0, 3.9 Hz, 1H, H4'), 4.12 (dd, *J* = 3.0, 1.4 Hz, 1H, H2'), 3.99 – 3.90 (m, 2H, H6b', H6a), 3.81 (dd, *J* = 12.1, 4.6 Hz, 1H, H6b), 3.68 (t, *J* = 8.8 Hz, 1H, H3), 3.65 – 3.54 (m, 5H, H4, H5, -OCH_3), 3.35 (m, 1H, H3'), 3.28 (dd, *J* = 9.5, 7.9 Hz, 1H, H2); ^{13}C RMN (126 MHz, D_2O) δ 137.5 (C_{hipso}, Ph), 130.6 (Ph), 129.3 (Ph), 103.7 (C1), 102.8 (C1′), 102.8 (C7), 78.7 (C4), 77.3 (C3), 76.1 (C4′), 75.0 (C5), 73.7 (C2), 70.7 (C2′), 68.9 (C6′), 61.4 (C6), 59.4 (C5′), 57.8 (-OCH_3), 51.3 (C3′); HRMS (ESI) m/z calcd for $C_{20}H_{29}NO_{10}$ [M+H$^+$]: 444.1864 , found: 444.1865.

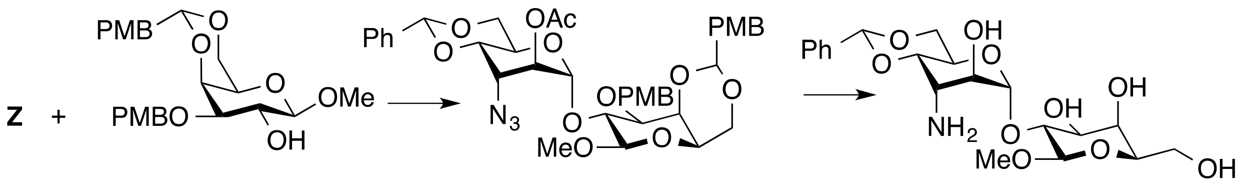

C1 **C2** **C**

Methyl 2-*O*-(3-amino-4,6-*O*-benzylidene-3-deoxy-α-D-altropyranosyl)- β-D-galactopyranoside (**C**). This compound was prepared following the general procedure of glycosylation from donor **Z** (173 mg, 0.362 mmol) and acceptor **C1**[14] (100 mg, 0.231 mmol) at -65ºC for 1 h. The crude material was purified by column chromatography on silica gel using hexane/EtOAc (6:4) to give glycosylated compound **C2** (166 mg, 96 %): ^{1}H NMR (400 MHz, $CDCl_3$): δ 7.51 – 7.43 (m, 4H, Ar), 7.41 – 7.33 (m, 5H, Ar), 6.92 – 6.79 (m, 4H, Ar), 5.57 (s, 1H, H7′), 5.43 (s, 1H, PMBCH), 5.09 (s, 1H, H1a), 5.03 (d, J=2.6 Hz, 1H, H2′), 4.69 (s, 2H, PMBCH_2-), 4.64 (td, J = 10.0, 5.2 Hz, 1H, H5′), 4.32 (d, J = 7.9 Hz, 1H, H1), 4.29 (m, 1H, H6), 4.17 (dd, J = 10.2, 5.3 Hz, 1H, H6′), 4.14 – 3.97 (m, 4H), 3.92 (dd, J = 9.7, 7.9 Hz, 1H, H2), 3.81 (s, 3H), 3.68 (s, 3H), 3.64 (d, J = 10.3 Hz, 1H, H6′), 3.56 (m, 1H, H3) 3.53 (s, 3H), 3.31 (s, 1H, H5), 2.15 (s, 3H, OAc); ^{13}C RMN (101 MHz, $CDCl_3$) δ ^{13}C NMR (101 MHz, $CDCl_3$) δ 169.5, 160.4, 159.6, 137.7, 130.8, 130.2, 130.1, 129.4, 128.6, 128.1, 126.6, 114.1, 113.8, 105.0 (C1), 102.4 (C7′), 101.5, 97.7 (C1′), 77.3 (C3), 76.3 (C4′), 75.1 (C2), 73.4 (C5), 71.3, 70.6 (C2′), 69.4 (C6), 69.3

[14] Prepared from methyl β-D-galactopyranoside: See J. Chen, L. Feng and G. D. Prestwich, *J. Org. Chem.* 1998, **63**, 6511.

(C6′), 66.8 (C5), 59.1 (C5′), 58.0 (C3′), 57.7, 55.6, 55.5, 21.3 (Me); HRMS (ESI) m/z calcd for $C_{38}H_{47}N_4O_{13}$ [M+ NH_4^+]: 767.3134, found: 767.3165.

Compound **C2** (150 mg, 0.200 mmol) was subjected to the general procedure for deprotection of the PMB protecting groups by treatment with di-*tert*-butyl pyridine (0.134 mL, 0.6 mmol) and DDQ (68 mg, 0.3 mmol) over a course of 1.5 h (method B). The crude material was purified by column chromatography using hexane/EtOAc (6:4). The resulting residue (108 mg, 0.17 mmol) was reacted with Selectfluor (91 mg) at rt. according to the general procedure for the cleavage of *p*-methoxybenzylidene groups and then purified by flash chromatography using CH_2Cl_2/MeOH (gradient 98:2, 96:4 then 94:6). This material (42.7 mg, 0.084 mmol) was finally treated with PMe_3 (125 μL, 0.125 mmol) and 0.1 M NaOH (168 μL) according to the general procedure of reduction of the azido moiety. The material was purified by column chromatography on silica gel using CH_2Cl_2/MeOH (90:10) to give the desired product **C** (30 mg, 34% overall yield): ^{1}H RMN (500 MHz, D_2O) δ 7.56–7.51 (m, 2H, Ph), 7.49–7.41 (m, 3H, Ph), 5.81 (s, 1H, H7′), 5.13 (s, 1H, H1′), 4.43 (m, 2H, H1, H5′), 4.33 (dd, *J* = 10.2, 5.1 Hz, 1H, H6a′), 4.13 (dd, *J* = 10.3, 4.0 Hz, 1H, H4′), 4.01 (d, *J* = 3.2 Hz, 1H, H2′), 3.92 (d, *J* = 3.6 Hz, 1H, H4), 3.89 (t, *J* = 10.3 Hz, 1H, H6b′), 3.82 – 3.70 (m, 3H, H6a, H6b, H3), 3.69 - 3.64 (m, 1H, H5), 3.58 (m, 4H, -OCH_3, H2), 3.32 (d, *J* = 3.4 Hz, 1H, H3′); ^{13}C NMR (126 MHz, D_2O) δ 137.51 (C_{hipso}, Ph), 129.41 (Ph), 126.90 (Ph), 104.74 (C1g), 102.81 (C7′), 100.80 (C1′), 76.83 (C2), 76.16 (C4′), 75.63 (C5), 71.93 (C3), 70.57 (C2′), 69.62 (C4), 69.07 (C6′), 61.56 (C6), 58.93 (C5′), 57.90 (-OCH_3), 51.45 (C3′); HRMS (ESI) m/z calcd for $C_{20}H_{29}NO_{10}$ [M+H^+]: 444.1864, found: 444.1880.

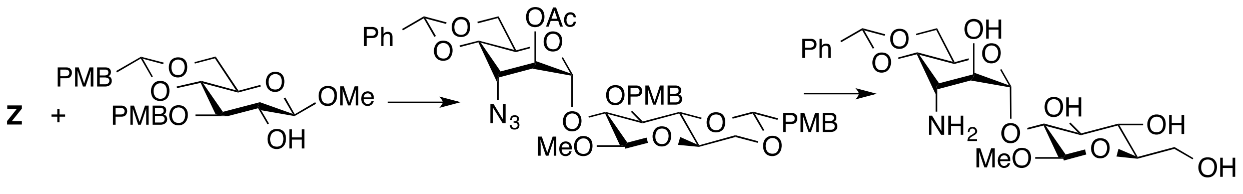

D1 D2 D

Methyl 2-*O*-(3-amino-4,6-*O*-benzylidene-3-deoxy-α-D-altropyranosyl)- β-D-glucopyranoside (D). This compound was prepared following the general procedure of glycosylation from donor **Z** (305 mg, 0.64 mmol) and acceptor **D1**[15] (100 mg, 0.23 mmol) at -65ºC for 1.5 h. The crude material was purified by column chromatography on silica gel using hexane/EtOAc (7:3) to give glycosylated compound **D2** (173 mg, quantitative): ^{1}H NMR (400 MHz, $CDCl_3$): δ 7.55 – 7.34 (m, 9H, Ar), 6.94 – 6.89 (m, 2H, Ar),

[15] J. Chen, L. Feng and G. D. Prestwich, *J. Org. Chem.* 1998, **63**, 6511.

6.71–6.66 (m, 2H, Ar), 5.61 (s, 1H), 5.54 (s, 1H), 5.15 (s, 1H, H1′), 5.04 (dd, J = 2.7, 0.8 Hz, 1H, H2′), 4.88 (d, J = 10.1 Hz, 1H, C<u>H</u>$_2$PMB), 4.82 (d, J = 10.1 Hz, 1H, C<u>H</u>$_2$PMB), 4.65 (td, J = 10.1, 5.3 Hz, 1H), 4.45 (d, J = 7.8 Hz, 1H, H1), 4.35 (dd, J = 10.5, 5.0 Hz, 1H), 4.13 – 4.03 (m, 3H), 3.82 (s, 3H, OCH_3), 3.81 – 3.65 (m, 5H), 3.59 (s, 3H, OCH_3), 3.54 (s, 3H, OCH_3), 3.44 (td, J = 9.9, 5.0 Hz, 1H), 2.15 (s, 3H, OAc); ^{13}C RMN (101 MHz, $CDCl_3$) δ 169.3, 160.2, 159.4, 137.5, 130.9, 129.9, 129.3, 128.5, 127.5, 126.4, 114.0, 113.8, 105.4 (C1), 102.2 (C7′), 101.5, 96.9 (C1′), 82.3, 78.6, 77.0, 76.2, 75.2, 70.4 (C2′), 69.0, 68.9, 66.3, 58.8, 58.0, 57.8, 55.5, 55.2, 21.2 (AcO); HRMS (ESI) m/z calcd for $C_{38}H_{44}N_3O_{13}$ [M+H$^+$]: 750.2869, found: 750.2851 [M+H]$^+$.

Compound **D2** (160 mg, 0.21 mmol) was subjected to the general procedure for deprotection of the PMB protecting groups by treatment with di-*tert*-butyl pyridine (0.14 mL, 0.642 mmol) and DDQ (73 mg, 0.321 mmol) overnight (method B). The crude material was purified by column chromatography using hexane/EtOAc (7:3). The resulting residue (95.6 mg, 0.15 mmol) was reacted with Selectfluor (80 mg) at rt. according to the general procedure for the cleavage of *p*-methoxybenzylidene groups and then purified by flash chromatography using CH_2Cl_2/MeOH (gradient 98:2 to 96:4). This material (56.3 mg, 0.110 mmol) was finally treated with PMe_3 (165 μL, 0.165 mmol) and 0.1 M NaOH (220 μL) according to the general procedure of reduction of the azido moiety. The material was purified by column chromatography on silica gel using CH_2Cl_2/MeOH (gradient 95:5 to 90:10) to give the desired product **D** (25 mg, 27%, overall yield): ^{1}H RMN (500 MHz, D_2O) δ 7.55 (m, 2H, Ph), 7.50–7.44 (m, 3H, Ph), 5.84 (s, 1H, H7′), 5.16 (s, 1H, H1′), 4.52 (d, J = 7.9 Hz, 1H, H1), 4.46 (td, J = 10.2, 5.0 Hz, 1H, H5′), 4.32 (dd, J = 10.4, 5.1 Hz, 1H, H6a′), 4.20 (dd, J = 10.3, 4.0 Hz, 1H, H4′), 4.09 – 4.04 (m, 1H, H2′), 3.97–3.88 (m, 2H, H6b′, H6), 3.72 (dd, J = 12.3, 5.7 Hz, 1H, H6), 3.66 – 3.59 (m, 1H, H3), 3.58 (s, 3H, -OCH_3), 3.49–3.35 (m, 4H, H2, H4, H5, H3′); ^{13}C NMR (126 MHz, D_2O) δ 137.5, 129.6, 126.9, 126.8, 104.3 (C1), 102.8 (C7′), 100.4 (C1′), 78.5 (C2), 76.3, 75.4 (C4′), 74.8 (C3), 70.5, 69.9 (C2′), 69.0 (C6a′), 61.3 (C6), 59.0 (C5′), 57.9 (-OCH_3), 51.2 (C3′); HRMS (ESI) m/z calcd for $C_{20}H_{29}NO_{10}$ [M+H$^+$]: 444.1864 , found: 444.1860.

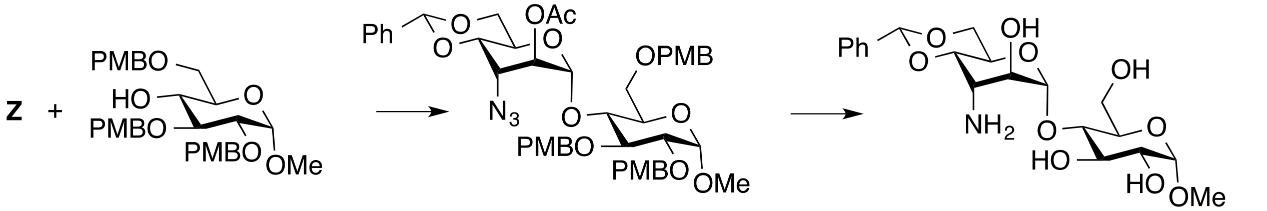

E1 E2 E

Methyl 2-*O*-(3-amino-4,6-*O*-benzylidene-3-deoxy-α-D-altropyranosyl)- α-D-glucopyranoside (E). This compound was prepared following the general procedure of glycosylation from donor **Z** (203 mg,

0.42 mmol) and acceptor **E1**[13] (120 mg, 0.21 mmol) at -65ºC for 1 h. The crude material was purified by column chromatography on silica gel CH_2Cl_2/MeOH (98:2) to give glycosylated compound **E2** (11 mg, 10%) in which cleavage of the PMB-ethers was observed: ^{1}H NMR (400 MHz, $CDCl_3$): δ 7.50 (qd, J = 5.0, 4.2, 2.0 Hz, 3H), 7.44 – 7.34 (m, 2H), 5.63 (s, 1H), 5.30 (d, J = 0.9 Hz, 1H, H1'), 5.06 (dd, J = 2.6, 0.8 Hz, 1H, H2'), 4.78 (d, J = 3.6 Hz, 1H, H1), 4.41 – 4.27 (m, 2H), 4.13 – 4.03 (m, 2H), 3.93 (dt, J = 14.4, 4.6 Hz, 3H), 3.85 – 3.59 (m, 4H), 3.50 (td, J = 12.0, 10.7, 5.2 Hz, 1H), 3.43 (s, 3H, -OCH_3), 3.16 (qd, J = 7.5, 4.9 Hz, 1H), 3.02 (s, 1H), 2.15 (s, 3H, OAc).

Compound **E2** (10 mg, 0.021 mmol) was treated with PMe_3 (40 μL, 0.04 mmol) and 0.1 M NaOH (60 μL) according to the general procedure of reduction of the azido moiety. The material was purified by column chromatography on silica gel using CH_2Cl_2/MeOH/Dioxane/NH_3 (7:2:1.5:0.5) to give the desired product **E** (3 mg, 33%): ^{1}H NMR (500 MHz, D_2O) δ 7.61 – 7.54 (m, 2H), 7.50 (m, 3H), 5.89 (s, 1H), 5.22 (s, 1H, H-1'), 4.84 (d, *J* = 3.8 Hz, 1H, H-1), 4.38 (dd, *J* = 10.3, 4.7 Hz, 1H, H-6'), 4.36 – 4.29 (m, 2H, H-4', H-5'), 4.27 (s, 1H, H-2'), 4.00 (t, *J* = 9.9 Hz, 1H, H-6'), 3.92 (dd, *J* = 12.0, 2.4 Hz, 1H, H-6), 3.90 – 3.80 (m, 3H, H-3, H-5, H-6), 3.70 – 3.64 (m, 2H, H-3', H-4), 3.61 (dd, *J* = 9.9, 3.8 Hz, 1H, H-2), 3.45 (s, 3H, -OCH_3). ^{13}C NMR (126 MHz, D_2O) δ 137.5, 129.0, 126.3, 102.3, 101.8 (C-1'), 99.2 (C-1), 78.9 (C-4), 73.3 (C-4'), 73.2 (C-3), 71.1 (C-2), 69.9 (C-5), 68.2 (C-6'), 68.1 (C-2'), 60.8 (C-6), 59.2 (C-5'), 55.1 (-OCH_3), 50.2 (C-3'). HRMS (ESI) m/z calcd for $C_{20}H_{30}NO_{10}$ [M+H$^+$]: 444.1864 , found: 444.1851.

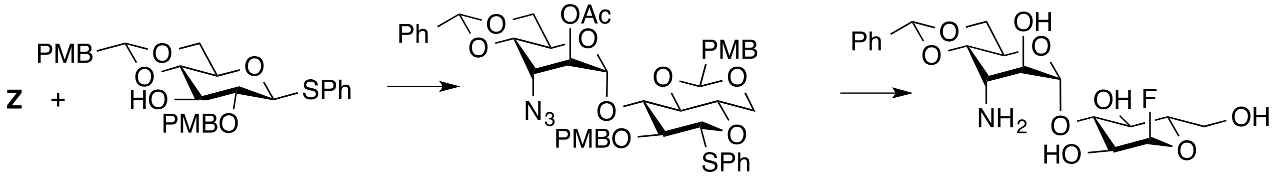

F1 **F2** **F**

3-*O*-(3-amino-4,6-*O*-benzylidene-3-deoxy-α-D-altropyranosyl)- α-D-glucopyranosyl fluoride (**F**). The compound was prepared following the general procedure of glycosylation from donor **Z** (313 mg, 0.65 mmol) and acceptor **F1**[16] (100 mg, 0.196 mmol) at -65ºC for 20 min. The crude material was purified by column chromatography on silica gel using hexane/EtOAc (8:2 then 7:3) to give compound **F2** (124.4 mg, 77 %): ^{1}H NMR (400 MHz, $CDCl_3$): δ 7.60–7.46 (m, 4H, Ph), 7.45–7.28 (m, 10H, Ph), 6.86–6.79 (m, 2H, Ph), 6.68–6.61 (m, 2H, Ph), 5.60 (s, 1H, H7΄), 5.52 (s, 1H, PMPCH), 5.28 (d, *J* = 0.9 Hz, 1H, H1΄), 5.14 (dd, *J* = 2.7, 0.9 Hz, 1H, H2΄), 5.04 (d, *J* = 9.2 Hz, 1H, PMBCH$_2$), 4.84 (d, *J* = 9.2

[16] K. Suzuki, I. Ohtsuka, T. Kanemitsu, T. Ako and O. Kanie, *J. Carbohyd. Chem.* 2005, **24**, 219.

Hz, 1H, PMBCH$_2$), 4.76 (d, *J* = 9.8 Hz, 1H, H1), 4.54 (td, *J* = 10.0, 5.2 Hz, 1H, H5′), 4.35 (dd, *J* = 10.6, 4.9 Hz, 1H, H6a), 4.16 – 3.98 (m, 4H, H3′, H4′, H6a′, H3), 3.82 – 3.74 (m, 5H, -OCH$_3$, H4, H6b), 3.65 (t, *J* = 10.4 Hz, 1H, H6b′), 3.60 (s, 3H, -OCH$_3$), 3.56 – 3.49 (m, 1H, H2), 3.47 – 3.39 (m, 1H, H5), 2.04 (s, 3H, -OAc), ^{13}C NMR (101 MHz, CDCl$_3$) δ 169.28, 160.26, 159.62, 137.58, 133.61, 132.38, 130.76, 129.41, 128.60, 127.70, 126.52, 114.22, 113.68, 102.23 (C7′), 101.29 (PMBCH), 97.69 (C1′), 89.04 (C1), 82.14 (C4), 79.10 (C2), 76.21 (C4′), 75.99, 70.39 (C2′), 70.08 (C5), 69.07 (C6′), 68.89 (C6), 59.05 (C5′), 58.16 (C3′), 55.62 (-OCH$_3$), 55.42 (-OCH$_3$), 21.41 (-OAc). HRMS (ESI) m/z calcd for $C_{43}H_{46}N_3O_{12}S$ [M+H$^+$]: 828.2797, found: 828.2795.

A solution of NIS (40.6 mg, 0.181 mmol) in dry CH_2Cl_2 (3 mL) was cooled to -40°C. HF-pyridine complex (54.5 µL, 3 mmol) was then added and the solution was stirred for 5 min.[17] A solution of **F2** (124.4 mg, 0.150 mmol) in dry CH_2Cl_2 (2 mL) was then added dropwise. After 1.5 h, the reaction mixture was quenched with pyridine and washed with 10% aqueous sodium thiosulfate containing $NaHCO_3$, and then water. The organic layer was dried, concentrated, and the residue purified by flash chromatography (hexane/EtOAc 7:3) to afford an anomeric mixture of highly unstable glycosyl fluorides (63 mg, 57%). ^{1}H NMR (500 MHz, CDCl$_3$): Selected peaks δ: 5.42 (dd, *J* = 52.2, 2.9 Hz, 1H, H1 α-anomer); 5.31 (dd, *J* = 52.5, 6.7 Hz, 1H, H1β-anomer) ; ^{13}C NMR (126 MHz, CDCl$_3$): Selected peaks δ: 105.81 (d, *J* = 230.6 Hz, C1 α-anomer); 109.72 (d, *J* = 216.7 Hz, C1 β-anomer). This material (53.5 mg, 0.073 mmol) was immediately subjected to the general procedure for deprotection of the PMB protecting groups (method B) over a course of 5 h. The crude material was purified by column chromatography using hexane/EtOAc (8:2) and the resulting residue (32 mg, 0.052 mmol) was reacted with Selectfluor (28 mg) at 50 ºC according to the general procedure for the cleavage of *p*-methoxybenzylidene groups and then purified by flash chromatography using CH_2Cl_2/MeOH (gradient 98:2 to 96:4). The residue (12.8 mg, 0.026 mmol) was treated with PMe$_3$ (40 µL, 0.04 mmol) and 0.1 M NaOH (30 µL) according to the general procedure of reduction of the azido moiety. The material was purified by column chromatography on silica gel using CH_2Cl_2/MeOH (90:10) to give the desired product **F** as pure α-anomer (13 mg, 20 % overall yield) ^{1}H NMR (500 MHz, D$_2$O): δ 7.57 (m, 2H, Ph), 7.49 (m, 3H, Ph), 5.87 (s, 1H, H7′), 5.73 (dd, *J* = 53.0, 2.9 Hz, 1H, H1), 5.20 (s, 1H, H1′), 4.51 (td, *J* = 10.3, 5.1 Hz, 1H, H5′), 4.33 (dd, *J* = 10.3, 5.2 Hz, 1H, H6′), 4.17 (dd, *J* = 10.1, 4.0 Hz, 1H, H4′), 4.15 – 4.11 (m, 1H, H2′), 3.94 (t, *J* = 10.3 Hz, 1H, H6a′), 3.91 – 3.75 (m, 5H, H6, H4, H2, H3), 3.75 – 3.69

[17] J. C. López, P. Bernal-Albert, C. Uriel and A. M. Gómez, *Eur. J. Org. Chem.* 2008, 5037.

(m, 1H, H5), 3.36 (m, 1H, H3′); ^{13}C NMR (126 MHz, D_2O): δ 137.51 (C_{hipso}, Ph), 129.63 (Ph), 127.01 (Ph), 108.37 (d, J = 225 Hz, C1), 102.81 (C7′), 102.18 (C1′), 79.93 (C3), 76.34 (C4′), 74.55 (C4), 70.69 (C2′), 70.47 (C2), 69.78 (C5), 69.11 (C6′), 60.55 (C6), 58.89 (C5′), 51.55 (C3′); HRMS (ESI) m/z calcd for $C_{19}H_{27}FNO_9$ [M+H$^+$]: 432.1664, found: 432.1670.

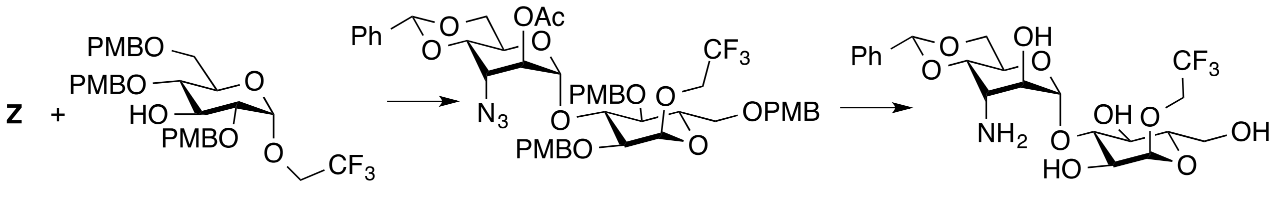

G1 G2 G

Trifluoroethyl 3-*O*-(3-amino-4,6-*O*-benzylidene-3-deoxy α-D-altropyranosyl)- α-D-glucopyranoside (**G**). The compound was prepared following the general procedure of glycosylation from donor **Z** (159 mg, 0.33 mmol) and acceptor **G1**[18] (68 mg, 0.11 mmol) at -65 ºC for 20 min. The crude material was purified by column chromatography on silica gel using hexane/EtOAc (gradient 8:2 to 7:3) to give compound **G2** (60 mg, 56%): ^{1}H NMR (300 MHz, $CDCl_3$): 7.54-7.34 (m, 5H, Ph), 7.28-7.17 (m, 4H, PMB), 7.05-6.70 (m, 8H, PMB), 5.55 (s, 1H, H7'), 5.12 (s, 1H, H1'), 5.05 (d, J=2.9 Hz, 1H, H2'), 4.87 (d, J=12.2 Hz, 1H, PMB), 4.78 (d, J=3.7 Hz, 1H, H1), 4.68 (d, J=11.7 Hz, 1H, PMB), 4.67 (d, J=12.2 Hz, 1H, PMB), 4.63 (ddd, J=9.6, 9.2, 6.2 Hz, 1H, H5'), 4.59 (d, J=12.2 Hz, 1H, PMB), 4.52 (d, J=11.7 Hz, 1H, PMB), 4.44 (d, J=12.2 Hz, 1H, PMB), 4.24-4.13 (m, 2H, H5, H6a'), 4.10 (dd, J=6.2, 3.1 Hz, 1H, H6b'), 4.05 (dd, J=4.8, 2.9 Hz, 1H, H3'), 3.99 (dd, J=9.6, 3.8 Hz, 1H, H4'), 3.86 (dd, J=7.8 Hz; $^3J_{H-F}$=17.3 Hz, 2H, $-OCH_2CF_3$), 3.79 (s, 3H, PMB), 3.77 (s, 3H, PMB), 3.74 (s, 3H, PMB), 3.80-3.67 (m, 3H, H3, H4, H6a), 3.61 (d, J=9.2 Hz, 1H, H2), 3.56 (dd, J=10.0, 3.7 Hz, 1H, H6b), 1.97 (s, 3H, AcO); ^{13}C RMN (75 MHz, $CDCl_3$); 168.9 (CO), 159.6, 159.2, 137.5, 130.3, 130.0 (CH, Ph), 129.9, 129.6, 129.0, 128.8, 128.2, 128.1, 126.3, 114.1, 114.0, 113.8, 102.2 (C7′), 98.2 (CH, C1), 97.7 (CH, C1'), 78.7 (CH, C4), 77.3 (CH, C3), 76.1 (CH, C4'), 74.1 (CH_2, PMB), 73.3 (CH, C5; CH_2, PMB), 72.8 (CH_2, PMB), 71.0 (CH, C2), 70.7 (CH, C2'), 69.0 (CH_2, C6'), 67.9 (CH_2, C6), 58.7 (CH, C5'), 57.8 (CH, C3'), 55.2, 55.0, 20.6 (AcO). MS (API-ES positive mode): 957 $(M+NH_4)^+$; HRMS (ESI) m/z calcd for $C_{47}H_{56}F_3N_4O_{14}$: $[M+NH_4]^+$: 957.3740, found: 957.3777.

[18] This compound was prepared in a similar manner than compound **A6** but using trifluoroethanol instead of methanol and concentrated sulphuric acid as catalyst in the opening of 3-*O*-allyl-1,2:4,6-bis-*O*-isopropylidene-α-D-glucofuranose. See: Y. Yoshimoto and Y. Tsuda, *Chem. PharmBull.* 1983, **31**, 4335.

Compound **G2** (50 mg, 0.05 mmol) was subjected to the general procedure for deprotection of the PMB protecting groups (method A) over a course of 5 h. The crude material was purified by column chromatography using CH_2Cl_2/MeOH (95:5) and the resulting residue (11.5 mg, 0.016 mmol) was treated with PMe_3 (30 μL, 0.03 mmol) and 0.1 M NaOH (40 μL) according to the general procedure of reduction of the azido moiety. The material was purified by column chromatography on silica gel using CH_2Cl_2/MeOH/Dioxane/NH_4OH mixture (7:2:1.5:1) to give the desired product **7** (8 mg, 31% overall yield): ^{1}H NMR (500 MHz, D_2O): δ 7.57 (m, 2H, Ph), 7.49 (m, 3H, Ph), 5.87 (s, 1H, H7′), 5.73 (dd, *J* = 53.0, 2.9 Hz, 1H, H1), 5.20 (s, 1H, H1′), 4.51 (td, *J* = 10.3, 5.1 Hz, 1H, H5′), 4.33 (dd, *J* = 10.3, 5.2 Hz, 1H, H6a′), 4.17 (dd, *J* = 10.1, 4.0 Hz, 1H, H4′), 4.15 – 4.11 (m, 1H, H2′), 3.94 (t, *J* = 10.3 Hz, 1H, H6b′), 3.91 – 3.75 (m, 5H, H6, H4, H2, H3), 3.75 – 3.69 (m, 1H, H5), 3.36 (m, 1H, H3′); ^{13}C RMN (126 MHz, D_2O) δ 137.5, 129.6, 127.0, 109.2 (C1), 107.4 (C1), 102.8 (C7′), 102.2 (C1′), 79.9 (C3), 76.3 (C4′), 74.5 (C4 or C5), 70.7 (C2′), 70.5 (C2), 69.7 (C5 or C4), 60.5 (C6), 58.9 (C5′), 51.6 (C3′); HRMS (ESI) m/z calcd for $C_{21}H_{29}F_3NO_{10}$ [M+H$^+$]: 512.1738, found: 512.1723.

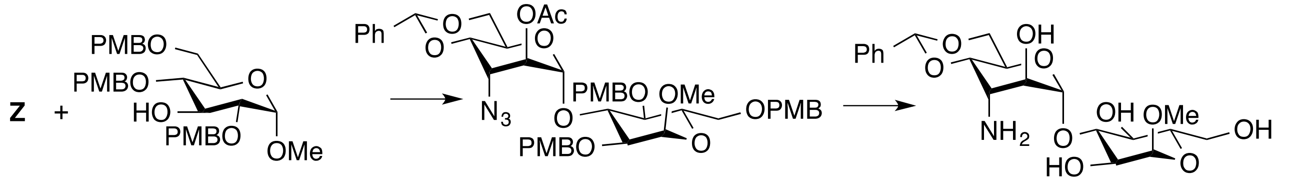

H1 **H2** **H**

Methyl 3-*O*-(3-amino-3-deoxy-4,6-*O*-benzylidene α-D-altropyranosyl)- α-D-glucopyranose (**H**).

The compound was prepared following the general procedure of glycosylation from donor **Z** (194 mg, 0.40 mmol) and acceptor **A6**[10] (75 mg, 0.13 mmol) at -65 ºC for 20 min. The crude material was purified by column chromatography on silica gel using hexane/EtOAc (gradient 8:2 to 7:3) to give compound **H2** (73 mg, 72%): ^{1}H NMR (300 MHz, $CDCl_3$): 7.52-7.33 (m, 5H, Ph), 7.28-6.95 (m, 6H, PMB), 6.88-6.75 (m, 6H, PMB), 5.57 (s, 1H, H7'), 5.11 (s, 1H, H1'), 5.05 (d, J=2.7 Hz, 1H, H2'), 4.80 (d, J=11.7 Hz, 1H, PMB), 4.67 (td, J=9.8, 5.2 Hz, 1H, H5'), 4.60 (d, J=11.7 Hz, 1H, PMB), 4.58 (d, J=11.5 Hz, 1H, PMB), 4.55 (d, J=10.0 Hz, 1H, PMB), 4.53 (s, 1H, H1), 4.40 (d, J=11.5 Hz, 1H, PMB), 4.37 (d, J=10.0 Hz, 1H, PMB), 4.30 (dd, J=10.1, 5.2 Hz, 1H, H6a'), 4.06 (dd, J=3.5, 2.7 Hz, 1H, H3'), 4.10-4.03 (m, 1H, H6b'), 3.99 (dd, J=9.4, 3.5 Hz, 1H, H4'), 3.78 (s, 3H, PMB), 3.77 (s, 3H, PMB), 3.71 (s, 3H, PMB), 3.70-3.62 (m, 4H, H3, H4, H5, H6a), 3.58 (d, J=9.8 Hz, 1H, H2), 3.49 (dd, J=9.8, 3.7 Hz, 1H, H6b), 3.32 (s, 3H, $-OCH_3$), 1.99 (s, 3H, AcO); ^{13}C RMN (75 MHz, $CDCl_3$); 168.9 (CO), 159.6,. 159.5, 159.2, 137.5, 130.5, 130.2, 130.1, 130.08, 129.6, 129.0, 128.6, 128.2, 126.3, 114.1, 113.9, 113.8, 102.2 (C7′), 98.3 (CH, C1), 98.1 (CH, C1'), 79.2 (CH, C4), 77.5 (CH, C3), 76.4 (CH, C4'), 73.9, 73.3,

72.89 (CH_2, PMB), 72.85 (C5), 70.8 (C2), 70.0 (C2'), 69.1 (C6'), 68.3 (C6), 58.8 (C5'), 57.8 (C3'), 55.3 (MeO), 55.23 (CH_3), 55.17 (CH_3), 55.1 (CH_3), 20.6 (CH_3). MS (API-ES positive mode): 889 $(M+NH_4)^+$; HRMS (ESI) m/z calcd for $C_{46}H_{57}N_4O_{14}$: $[M+NH_4]^+$: 889.3866, found: 889.3861.

Compound **H2** (60 mg, 0.07 mmol) was subjected to the general procedure for deprotection of the PMB protecting groups (method A) over a course of 3 h. The crude material was purified by column chromatography using CH_2Cl_2/MeOH (95:5) and the resulting residue (16.5 mg, 0.032 mmol) was treated with PMe_3 (50 μL, 0.05 mmol) and 0.1 M NaOH (80 μL) according to the general procedure of reduction of the azido moiety. The material was purified by column chromatography on silica gel using CH_2Cl_2/MeOH/Dioxane/NH_4OH mixture (7:2:1.5:1) to give the desired product **H** (14 mg, 46% overall yield): ^1H NMR (500 MHz, D_2O) δ 7.54 (m, 2H, Ph), 7.46 (m, 3H, Ph), 5.82 (s, 1H, H7′), 5.17 (s, 1H, H1′), 4.81 (d, J = 3.8 Hz, 1H, H1), 4.47 (dt, J = 10.2, 5.0 Hz, 1H, H5′), 4.30 (dd, J = 10.4, 5.1 Hz, 1H, H6a′), 4.13 (dd, J = 10.2, 3.9 Hz, 1H, H4′), 4.08 (d, J = 2.8 Hz, 1H, H2′), 3.94 – 3.81 (m, 2H, H6b', H6a), 3.81 – 3.71 (m, 2H, H3, H6b), 3.69 – 3.62 (m, 2H, H2, H5), 3.57 (t, J = 9.4 Hz, 1H, H4), 3.42 (s, 3H, -OCH_3), 3.32 (t, J = 3.3 Hz, 1H, H3′); ^{13}C RMN (126 MHz, D_2O) δ 137.51 (C_{hipso}, Ph), 130.06 (2x Ph), 127.55 (2xPh), 103.17 (C7′), 102.30 (C1′), 100.36 (C1), 80.53 (C3), 76.49 (C4′), 71.44 (C2 and C5), 71.35 (C4), 70.87 (C2′), 69.22 (C6′), 61.14 (C6) , 58.97 (C5′), 55.74 (-OCH_3), 52.39 (C3′). HRMS (ESI) m/z calcd for $C_{20}H_{29}NO_{10}$ $[M+H^+]$: 444.1864, found: 444.1867.

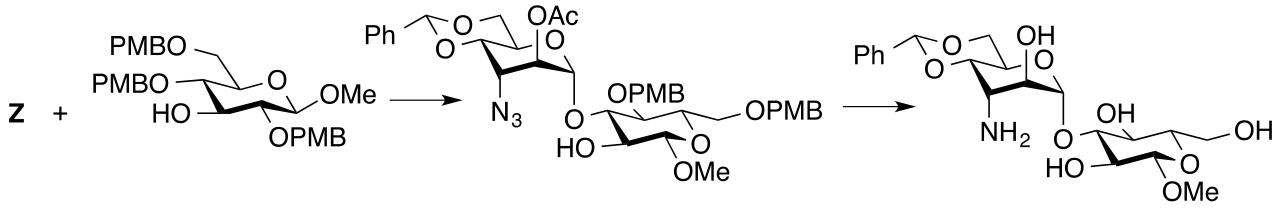

I1 I2 I

Methyl 3-*O*-(3-amino-4,6-*O*-benzylidene-3-deoxy- α-D-altropyranosyl)- β-D-glucopyranoside (I).

The compound was prepared following the general procedure of glycosylation from donor **Z** (188 mg, 0.39 mmol) and acceptor **I1**[19] (71 mg, 0.13 mmol) at -65 ºC for 30 min. The crude material was purified by column chromatography on silica gel using hexane/EtOAc (gradient 8:2 to 6:4) to give a compound which was identified as partially deprotected **I2** (33 mg, 38%): ^1H NMR (400 MHz, $CDCl_3$): 7.54-7.36 (m, 5H, Ph), 7.30-7.07 (m, 4H, PMB), 6.88-6.80 (m, 4H, PMB), 5.61 (s, 1H, H7'), 5.07 (d, J=2.7 Hz,

[19] This compound was prepared from 3-*O*-allyl-1,2:4,6-bis-*O*-isopropylidene-α-D-glucofuranose by methanolysis, as described in Y. Yoshimoto and Y. Tsuda, *Chem. PharmBull.* 1983, **31**, 4335, followed by per *p*-methoxybenzylation and allyl-cleavage. Separation of the anomers was carried out in the final step.

1H, H2’), 4.98 (s, 1H, H1’), 4.69 (d, J=10.5 Hz, 1H, PMB), 4.66 (ddd, J=10.5, 9.1, 5.3 Hz, 1H, H5’), 4.59 (d, J=12.1 Hz, 1H, PMB), 4.49 (d, J=12.1 Hz, 1H, PMB), 4.48 (d, J=10.5 Hz, 1H, PMB), 4.35 (dd, J=10.3, 5.3 Hz, 1H, H6b’), 4.20 (d, J=7.4 Hz, 1H, H1), 4.08 (dd, J=6.6, 3.5 Hz, 1H, H3’), 4.06 (dd, J=9.1, 3.5 Hz, 1H, H4’), 3.803 (s, 3H, PMB), 3.796 (s, 3H, PMB), 3.78-3.75 (m, 1H, H6a’), 3.71-3.62 (m, 4H, H3, H4, H6a, H6b), 3.58 (s, 3H, -OCH_3), 3.54 (dd, J=7.8, 7.4 Hz, 1H, H2), 3.42 (ddd, J=9.0, 3.9, 2.3 Hz, 1H, H5), 3.09 (bd, J=3.5 Hz, 1H, H-OH), 2.10 (s, 3H, AcO); ^{13}C RMN (101 MHz, $CDCl_3$); 169.0 (CO), 159.2, 159.2, 136.9, 130.4, 130.0, 129.6, 129.4, 129.2, 128.3, 126.1, 113.8, 113.7, 103.7 (C1), 102.3 (C7′), 98.8 (C1’), 84.0 (C4), 77.4 (C3), 75.7 (C4’), 75.0 (C5), 74.5, 73.2, 72.7 (CH, C2), 70.5 (C2’), 68.8 (C6’), 68.1 (C6), 59.2 (C5’), 57.5 (C3’), 57.2 (CH_3), 55.2, 20.8 (AcO). MS (API-ES positive mode): 769 $(M+NH_4)^+$; HRMS (ESI) m/z calcd for $C_{38}H_{49}N_4O_{13}$: $[M+NH_4]^+$: 769.3291, found: 769.3321.

Compound **I2** (30 mg, 0.04 mmol) was subjected to the general procedure for deprotection of the PMB protecting groups (method A) over a course of 3 h. The crude material was purified by column chromatography using CH_2Cl_2/MeOH (95:5) and the resulting residue (18 mg, 0.036 mmol) was treated with PMe_3 (50 μL, 0.05 mmol) and 0.1 M NaOH (80 μL) according to the general procedure of reduction of the azido moiety. The material was purified by column chromatography on silica gel using CH_2Cl_2/MeOH/Dioxane/NH_4OH mixture (7:2:1.5:1) to give the desired product **I** (15 mg, 85% overall yield): ^{1}H NMR (500 MHz, D_2O) δ 7.58 – 7.52 (m, 2H, Ph), 7.48 (m, 3H, Ph), 5.87 (s, 1H, H7′), 5.26 (s, 1H, H1′), 4.49 (td, *J* = 10.1, 4.9 Hz, 1H, H5′), 4.44 – 4.38 (m, 2H, H1g, H4′), 4.33 (dd, *J* = 10.4, 4.9 Hz, 1H, H6a′), 4.29 (dd, *J* = 2.8, 1.5 Hz, 1H, H2′), 3.98 (t, *J* = 10.2 Hz, 1H, H6b’), 3.92 (dd, *J* = 12.4, 2.3 Hz, 1H, H6a), 3.84 (t, *J* = 3.5 Hz, 1H, H3′), 3.73 (dd, *J* = 12.4, 5.7 Hz, 1H, H6b), 3.68 (t, *J* = 9.1 Hz, 1H, H3), 3.61 (m, 4H, H4g, -OCH_3), 3.48 (ddd, *J* = 10.0, 5.7, 2.3 Hz, 1H, H5), 3.46 – 3.41 (m, 1H, H2); ^{13}C RMN (126 MHz, D_2O) δ 137.51 (C_{hipso}, Ph), 127.0, 129.5 (Ph), 102.9 (C7′), 100.8 (C1′), 59.4 (C5′), 103.9 (C1), 72.7 (C4′), 68.9 (C6′), 67.6 (C2′), 61.0 (C6), 50.6 (C3′), 83.2 (C3), 70.6 (C4), 58.0 (-OCH_3), 76.2 (C5), 72.3 (C2); HRMS (ESI) m/z calcd for $C_{20}H_{29}NO_{10}$ $[M+H^+]$: 444.1864, found: 444.1876.

Dynamic combinatorial experiments.- As a first step, reductive amination reactions were performed in NMR tubes with single altrosamine derivatives. Thus, a mixture of the saccharide (~1 mM) and aldehyde (~2 mM) were dissolved in D_2O (10 mM phosphate, pH 6.2) and treated with sodium cyanoborohydride at room temperature. After reaction completion, final products were assigned employing a combination of 2D-COSY, TOCSY and HSQC experiments. From these data, we determined the chemical shift perturbations promoted by the aromatic units on the CH/π donor pyranoses. The obtained Δδ (ppm) values were taken as an indicative of the geometry of the different stacking complexes (Figures S3 and S5-S7).

Next, equimolecular mixtures of 6-8 altrosamine derivatives (~1 mM each) in D_2O (10 mM phosphate, pH 6.2) were treated with a sub-stoichiometric amount of a given arylacetaldehyde (compounds **1-13** in Figure 2b). The aldehyde concentration was carefully adjusted to achieve maximum conversions of the altrosamine derivatives not superior to 40% (usually in the 400-800 μM range depending on the number of components present in the mixture). All dynamic combinatorial assays were carried out at low temperature in order to maximize the stability of the CH/π complexes and consequently, the sensitivity of the experiments. Thus, mixtures were left to equilibrate for 2 hours at 277 K, and then we added sodium cyanoborohydride (5 mM). Reactions were kept overnight at 277 K. After reaction completion, we performed a qualitative quantification of the reaction products by focusing our attention on the altrosamine anomeric signals in 2D-HSQC experiments acquired at 318 K.

In order to evaluate more precisely the stability of the different CH/π complexes considered in our study, we performed pairwise competition experiments following the same protocol. The quantification of the relative populations of the final products was accomplished by integrating key NMR signals in 1D experiments acquired at 318 K with long relaxation delays (d1>5). Overlapping signals were deconvoluted before integration employing the line-fitting module implemented in MestreNova software. The obtained values reflect the population of the corresponding imine/hemiaminal

intermediates and therefore, were employed to derive the relative stability (ΔΔG) of the alternative stacking modes according to ΔΔG=R*277*Ln(P_1/P_2) (where P_1/P_2 represents the population ratio and 277 is the reaction and equilibration temperatures in K). This protocol has been recently validated by our group employing structurally related allosamine disaccharides (for which imine/hemiaminal intermediates could be detected and their populations measured).[20]

We performed pairwise competitions with all possible **Ref**/disaccharide pairs so that the net free energy (ΔG) of the stacking complexes could be determined. Selected data points were derived from at least two independent reactions in order to estimate errors. In these cases, product populations were also measured from NMR spectra acquired with distinct relaxation delays. Experimental errors were found to be dependent on the population ratios (P_1/P_2). Errors upper bounds were estimated as ±10% for $1<P_1/P_2<3$, ±15% for $3<P_1/P_2<6$ and ±20% for $6<P_1/P_2$. At 277 K these values translate into energy ranges of ±0.05, ±0.08, ±0.11 kcal/mol, respectively (Table S1).

In addition to the net interaction energies (ΔG), we also determined free energy differences (ΔΔG) of all the complexes with respect to those formed by **D**, by performing the corresponding pairwise competitions. Finally, many other crosschecks were carried out with selected aldehydes and disaccharide pairs (Figure S4). The obtained ΔΔG values were found to be fully consistent with the net interaction energies (ΔG) and errors represented in Table S1.

Quantum Mechanical Calculations.- Full geometry optimizations for the different THF/aromatic complexes were carried out with Gaussian 09[21] using the M06-2X functional[22] with an ultrafine integration grid and the triple-zeta TZVP basis set.[23] Two interaction modes, named as a and b, were assumed in each case to simulate the alternative geometries of complexes formed by derivatives **C**/**D**

---

[20] E. Jimenez-Moreno, A. M. Gomez, A. Bastida, F. Corzana, G. Jiménez-Osés, J. Jiménez-Barbero and J.L. Asensio, *Angew. Chem. Int. Ed.*, 2015, **54**, 4344.
[21] J. A. Pople, *et al.* GAUSSIAN 09, Revision D.01, Gaussian, Inc., Wallingford CT, 2009.
[22] Y. Zhao and D. G. Truhlar, Theor. *Chem. Acc.*, 2008, **120**, 215.
[23] (a) A. Schaefer, H. Horn and R. Ahlrichs, *J. Chem. Phys.*, 1992, **97**, 2571; (b) A. Schaefer, C. Huber, R. Ahlrichs, *J. Chem. Phys.*, 1994, **100**, 5829.

and **A**/**B**/**E**, respectively, upon the reductive amination reaction. Solvent effects were included in the optimization through the integral equation formalism variant of the polarizable continuum model (IEF-PCM),[24] using UFF radii and parameters for water, as implemented in Gaus-sian 09. Frequency analyses were carried out at the same level used in the geometry optimizations to evaluate the zero-point vibrational energy and thermal corrections at 298 K necessary to derive enthalpies and free energies. The nature of the stationary points was determined according to the appropriate number of negative eigenvalues of the Hessian matrix.

Gas-phase single-point energies at the spin-component scaled MP2 (SCS-MP2) level[25] with the triple-zeta 6-311G(2d,p) basis set, and also at the M06-2X/TZVP level (see Figure S10), were calculated on the optimized geometries using Gamess (v.2014).[26] Basis set superposition errors (BSSE) were corrected by the Boys–Bernardi counterpoise method.[27] Solvation free energies (ΔG_{solv}) were calculated at the ab initio level using the IEF-PCM method using the parameters for water as implemented in Gamess. Various contributions to the interaction energies (electrostatic, exchange, repulsion, polarization and dispersion) were estimated through the Localized Molecular Orbital Energy Decomposition Analysis (LMO-EDA) method[28] as implemented in Gamess.

---

[24] G. Scalmani and M. J. Frisch, *J. Chem. Phys.*, 2010, 132.
[25] S. J. Grimme, *Chem. Phys.*, 2003, **118**, 9095.
[26] (a) M. W. Schmidt, K. K. Baldridge, J. A. Boatz, S. T. Elbert, M. S. Gordon, J. H. Jensen, S. Koseki, N. Matsunaga, K. A. Nguyen, S. J. Su, T. L. Windus, M. Dupuis and J. A. Montgomery, *J. Comput. Chem.*, 1993, **14**, 1347; (b) M. S. Gordon and M. W. Schmidt, in Theory and Applications of Computational Chemistry, the first forty years. Chapter 41, pp 1167–1189, C. E. Dykstra, G. Frenking, K. S. Kim and G. E. Scuseria, editors. Elsevier, Amsterdam, 2005.
[27] S. F. Boys and F. Bernardi, *Mol. Phys.*, 1970, **19**, 553.
[28] P. Su and H. Li, *J. Chem. Phys.*, 2009, **131**, 1.

Molecular dynamics calculations.-The conformational and dynamical properties of selected complexes (Figure S8) were estimated through Molecular Dynamics (MD) simulations performed with the AMBER 12 package.[29] 100 ns trajectories were collected in the presence of explicit TIP3P water,[30] periodic boundary conditions and Ewald sums for the treatment of long-range electrostatic interactions, [31] following a protocol identical to that previously described.[32] In all cases, RESP atomic charges were derived by applying the RESP module of AMBER to the HF/6-31G(d) ESP charges calculated with Gaussian 09. The ffSB14 force field[33] was implemented with GLYCAM06[34] and GAFF[35] parameters to accurately simulate the conformational behavior of these molecules. The timestep was 1 fs in all the simulations.

---

[29] D. A. Case, T. A. Darden, T. E. Cheatham, III, C. L. Simmerling, J. Wang, R. E. Duke, R. Luo, R. C. Walker, W. Zhang, K.M. Merz, B. Roberts, S. Hayik, A. Roitberg, G. Seabra, J. Swails, A. W. Götz, I. Kolossváry, K. F. Wong, F. Paesani, J. Vanicek, R. M. Wolf, J. Liu, X. Wu, S. R. Brozell, T. Steinbrecher, H. Gohlke, Q. Cai, X. Ye, J. Wang, M.-J. Hsieh, G. Cui, D. R. Roe, D. H. Mathews, M. G. Seetin, R. Salomon-Ferrer, C. Sagui, V. Babin, T. Luchko, S. Gusarov, A. Kovalenko, and P.A. Kollman (2012), AMBER 12, University of California, San Francisco.

[30] W. L. Jorgensen, J. Chandrasekhar, J. D. Madura, R. W. Impey and M. L Klein, *J. Chem. Phys*., 1983, **79**, 926.

[31] T. Darden, D. York and L. Pedersen, *J. Chem. Phys*., 1993, **98**, 10089.

[32] D. Madariaga, N. Martínez-Sáez, V. J. Somovilla, L. García-García, M. A. Berbis, J. Valero-Gónzalez, S. Martín-Santamaría, R. Hurtado-Guerrero, J. L. Asensio, J. Jiménez-Barbero A. Avenoza, J. H. Busto, F. Corzana and J. M. Peregrina, *Chem. Eur. J.*, 2014, **20**, 12616.

[33] V. Hornak, R. Abel, A. Okur, B. Strockbine, A. Roitberg and C. Simmerling, *Proteins: Struct., Funct., Bioinf.*, 2006, **65**, 712.

[34] K. N. Kirschner, A. B. Yongye, S. M. Tschampel, J. González-Outeiriño, C. R. Daniels, B. L. Foley and R. J. Woods, *J. Comput. Chem*., 2008, **29**, 622.

[35] J. Wang, R. M. Wolf, J. W. Caldwell, P. A. Kollman and D. A. Case, *J. Comput Chem*., 2004, **25**, 1157.

Table S1.- Net interaction free energies (at 277 K, kcal/mol) measured for the CH/π complexes established between donors **A-I** and the aromatic rings present in **1-13**. These values were derived from pair-wise competition experiments in which each disaccharide was confronted with the reference compound (**Ref**). The ratio between the respective final products (measured by NMR) is indicated in brackets.

	**Model Disaccharides (A-I)**								
	α-face stacking					***β-face stacking***			
	A	***B***	***C***	***D***	***E***	***F***	***G***	***H***	***I***
4	-1.48±0.11 *(14.76)*	-1.45±0.11 *(14.05)*	-1.22±0.11 *(9.25)*	-1.10±0.11 *(7.40)*	-0.79±0.08 *(4.23)*	-1.24±0.11 *(9.50)*	-1.19±0.11 *(8.75)*	-1.06±0.11 *(6.95)*	-0.76±0.08 *(3.98)*
9	-1.44±0.11 *(13.64)*	-1.34±0.11 *(11.47)*	-1.15±0.11 *(8.05)*	-0.94±0.08 *(5.50)*	-0.70±0.08 *(3.60)*	-1.05±0.11 *(6.75)*	-1.03±0.11 *(6.45)*	-0.79±0.08 *(4.18)*	-0.59±0.05 *(2.90)*
10	-1.29±0.11 *(10.54)*	-1.34±0.11 *(11.50)*	-1.21±0.11 *(9.07)*	-0.93±0.08 *(5.41)*	-0.96±0.08 *(5.68)*	-0.98±0.08 *(5.92)*	-1.03±0.11 *(6.50)*	-0.82±0.08 *(4.46)*	-0.66±0.08 *(3.33)*
3	-1.45±0.11 *(14.00)*	-1.39±0.11 *(12.66)*	-1.18±0.11 *(8.60)*	-0.86±0.08 *(4.80)*	-0.70±0.08 *(3.60)*	-0.97±0.08 *(5.82)*	-0.98±0.08 *(5.90)*	-0.73±0.08 *(3.75)*	-0.50±0.05 *(2.50)*
1	-1.29±0.11 *(10.48)*	-1.26±0.11 *(9.86)*	-1.07±0.11 *(7.00)*	-0.74±0.08 *(3.84)*	-0.72±0.08 *(3.70)*	-0.95±0.08 *(5.57)*	-0.89±0.08 *(5.04)*	-0.74±0.08 *(3.85)*	-0.50±0.05 *(2.50)*
5	-1.12±0.11 *(7.70)*	-1.14±0.11 *(8.00)*	-0.80±0.08 *(4.30)*	-0.70±0.08 *(3.58)*	-0.53±0.05 *(2.63)*	-0.83±0.08 *(4.52)*	-0.78±0.08 *(4.16)*	-0.60±0.05 *(2.98)*	-0.52±0.05 *(2.58)*
6	-1.18±0.11 *(8.65)*	-1.14±0.11 *(7.92)*	-0.79±0.08 *(4.25)*	-0.62±0.08 *(3.06)*	-0.44±0.05 *(2.23)*	-0.73±0.08 *(3.80)*	-0.67±0.08 *(3.38)*	-0.66±0.08 *(3.30)*	-0.36±0.05 *(1.93)*
8	-1.07±0.11 *(7.05)*	-1.08±0.11 *(7.13)*	-0.89±0.08 *(5.00)*	-0.57±0.05 *(2.81)*	-0.65±0.08 *(3.25)*	-0.77±0.08 *(4.08)*	-0.76±0.08 *(3.98)*	-0.71±0.08 *(3.60)*	-0.44±0.05 *(2.23)*
7	-0.82±0.08 *(4.47)*	-0.84±0.08 *(4.60)*	-0.45±0.05 *(2.26)*	-0.25±0.05 *(1.57)*	-0,35±0.05 *(1.88)*	-0.38±0.05 *(2.00)*	-0.38±0.05 *(1.98)*	-0.28±0.05 *(1.67)*	-0.15±0.05 *(1.32)*
2	-0.76±0.08 *(4.00)*	-0.75±0.08 *(3.90)*	-0.54±0.05 *(2.67)*	-0.53±0.05 *(2.61)*	-0.76±0.08 *(4.00)*	-0.56±0.05 *(2.76)*	-0.58±0.05 *(2.86)*	-0.48±0.05 *(2.39)*	-0.50±0.05 *(2.48)*
11	-0.61±0.08 *(3.03)*	-0.64±0.08 *(3.24)*	-0.30±0,05 *(1.73)*	-0.29±0.05 *(1.72)*	-0.39±0.05 *(2.06)*	-0.45±0.05 *(2.27)*	-0.46±0.05 *(2.30)*	-0.35±0.05 *(1.89)*	-0.38±0.05 *(1.98)*
12	-0.65±0.08 *(3.26)*	-0.59±0.05 *(2.92)*	-0.29±0.05 *(1.70)*	-0.25±0.05 *(1.58)*	-0.37±0.05 *(1.97)*	-0.40±0.05 *(2.08)*	-0.43±0.05 *(2.20)*	-0.37±0.05 *(1.96)*	-0.37±0.05 *(1.96)*
13	- *(-)*	-0.83±0.08 *(4.56)*	0.00±0.05 *(1.00)*	0.00±0.05 *(1.00)*	-0.71±0.08 *(3.65)*	-0.12±0.05 *(1.25)*	0.00±0.05 *(1.00)*	0.00±0.05 *(1.00)*	-0.17±0.05 *(1.35)*

Table S2.- Interaction enthalpies (ΔH, kcal/mol) were calculated at the M06-2X/TZVP level in water (IEF-PCM method) for simplified inter-molecular complexes formed by tetrahydropyrane (THP, as a pyranose model) and the aromatic units present in **1-13** (Figure 7a, see experimental section for details), considering two orientations of the THP unit (referred as geometry **a** and **b**). From these geometries, single-point interaction energies (ΔE_{Total}, kcal/mol) were calculated at the SCS-MP216/6-311G(2d,p) level in the gas phase. In addition, we derived the electrostatic (ΔE_{Elect}, kcal/mol), polarization (ΔE_{Pol}, kcal/mol), exchange+repulsion (ΔE_{Ex-Rp}, kcal/mol) and dispersion (ΔE_{Disp}, kcal/mol) contributions, through the Localized Molecular Orbital Energy Decomposition Analysis (LMO-EDA). Finally, the penalties associated to THP and aromatic desolvation upon formation of the complexes (ΔE_{Solv}, kcal/mol) were evaluated at the same theory level. The corresponding values obtained for the different complexes are represented below.

	Geometry ***a***							*Geometry* ***b***						
	M06-2X/TZVP	SCS-MP216/6-311G(2d,p)						M06-2X/TZVP	SCS-MP216/6-311G(2d,p)					
	ΔH	***ΔE*** *Total*	***ΔE*** *Elect.*	***ΔE*** *Ex+Rp.*	***ΔE*** *Pol.*	***ΔE*** *Disp.*	***ΔE*** *Solv.*	***ΔH***	***ΔE*** *Total*	***ΔE*** *Elect.*	***ΔE*** *Ex+Rp.*	***ΔE*** *Pol.*	***ΔE*** *Disp.*	***ΔE*** *Solv.*
**THP-1**	-3.2	-2.8	-4.5	+10.2	-1.3	-7.3	+0.7	-3.2	-2.9	-4.4	+10.1	-1.2	-7.4	+0.9
**THP-3**	-3.1	-2.9	-4.7	+10.5	-1.3	-7.5	+0.8	-3.1	-2.9	-4.4	+10.4	-1.3	-7.6	+0.6
**THP-4**	-3.6	-3.3	-4.9	+11.3	-1.4	-8.2	+1.0	-4.0	-3.5	-5.0	+11.5	-1.3	-8.7	+1.4
**THP-5**	-3.8	-	-	-	-	-	-	-4.0	-	-	-	-	-	-
**THP-6**	-3.9	-	-	-	-	-	-	-4.3	-	-	-	-	-	-
**THP-7**	-3.8	-	-	-	-	-	-	-4.1	-	-	-	-	-	-
**THP-8**	-2.9	-2.6	-4.2	+10.2	-1.2	-7.4	+0.4	-2.9	-2.7	-4.2	+10.1	-1.2	-7.5	+0.5
**THP-9**	-3.4	-3.1	-5.0	+10.0	-1.4	-6.8	+1.4	-3.3	-3.0	-4.6	+9.7	-1.3	-6.7	+1.2
**THP-10**	-3.4	-3.1	-5.0	+10.1	-1.4	-6.8	+1.3	-3.2	-2.9	-4.5	+9.6	-1.3	-6.7	+0.9
**THP-11**	-2.7	-2.3	-3.7	+9.5	-1.0	-7.0	+0.3	-2.7	-2.5	-3.8	+9.3	-1.0	-7.0	+0.8
**THP-12**	-2.7	-2.1	-3.5	+9.2	-1.0	-6.9	+0.5	-2.6	-2.2	-3.3	+9.0	-1.0	-6.9	+0.3
**THP-13**	-2.5	-2.1	-3.0	+8.8	-0.9	-7.0	+0.7	-2.5	-2.5	-3.3	+8.7	-0.9	-6.9	+1.2

Figure S1.- 1D- (up), 2D-TOCSY (Bottom-Right) and 2D-HSQC (Bottom-Left) spectra acquired for compound **A** at 500 MHz, pH 7.5 and 298K.

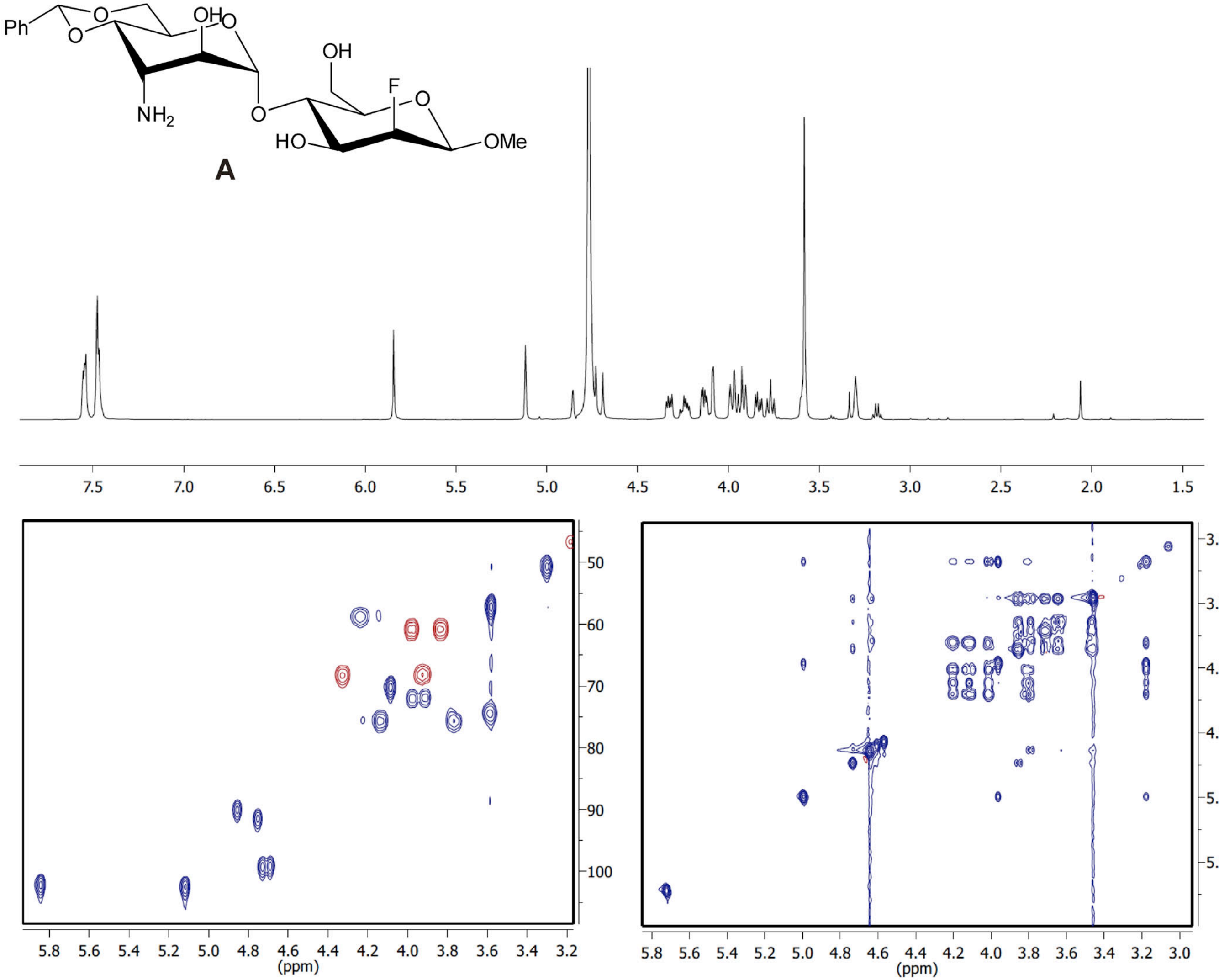

Figure S2.- 1D- (up), 2D-COSY (Bottom-Right) and 2D-HSQC (Bottom-Left) spectra acquired for compound **E** at 500 MHz, pH 7.5 and 298K.

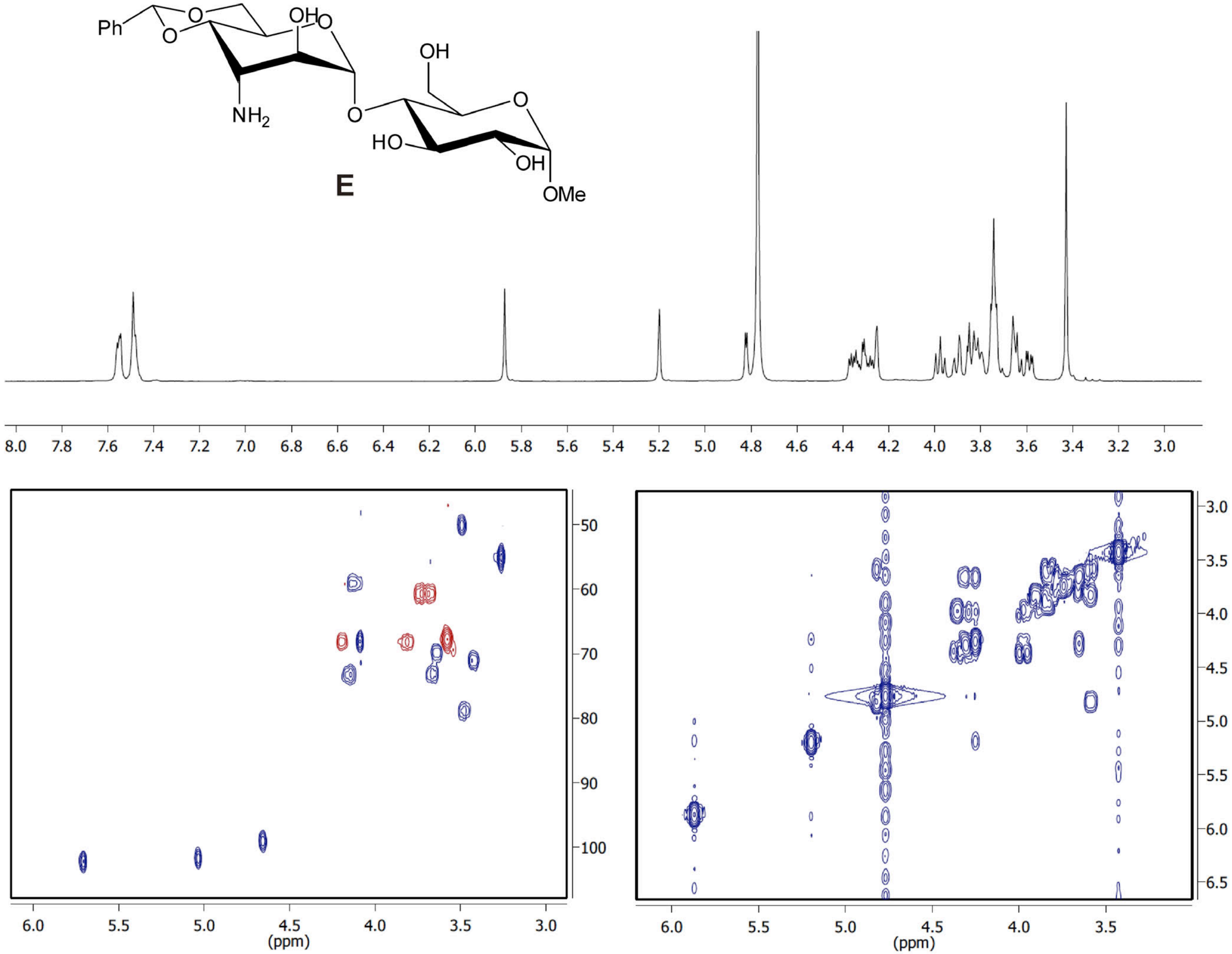

Figure S3.- Key region of 1D-NMR Spectra acquired for compounds **B-1**(Left) and **H-1** (Right). NMR signals for protons involved in CH/π interactions with the aromatic ring are highlighted with coloured circles. Chemical shift perturbations promoted by the aromatic ring at the interacting D-pyranose units are shown above. These data indicate that complexation is mediated by the pyranose α-face in **B-1** and by the β-face in **H-1**.

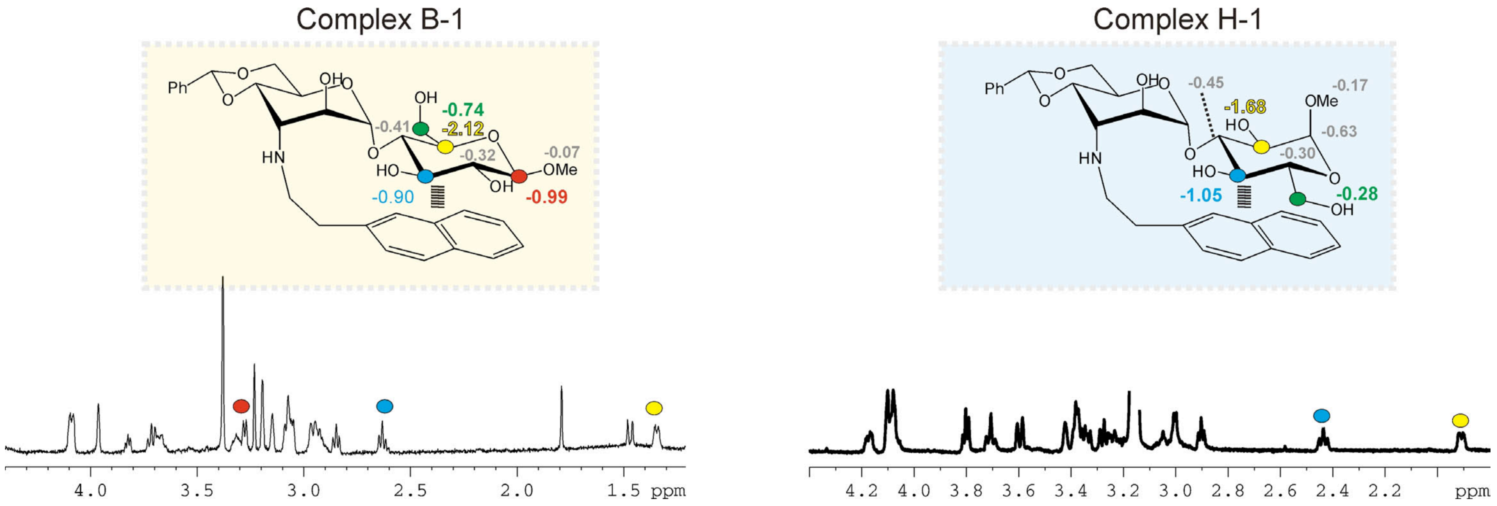

Figure S4.- We performed pairwise competitions with all possible **Ref/disaccharide** pairs so that the net free energy (ΔG) of the stacking complexes could be determined (upper panel). In addition, crosschecks were carried out with selected aldehydes and disaccharide pairs (lower panel). The obtained ΔΔG values were found to be fully consistent with the net interaction energies (ΔG) and errors represented in Table S1. NMR signals for the altrose anomeric proton in the final products, together with the product ratios (grey. In brackets) and the estimated free energy differences between alternative interaction modes (in black) are shown.

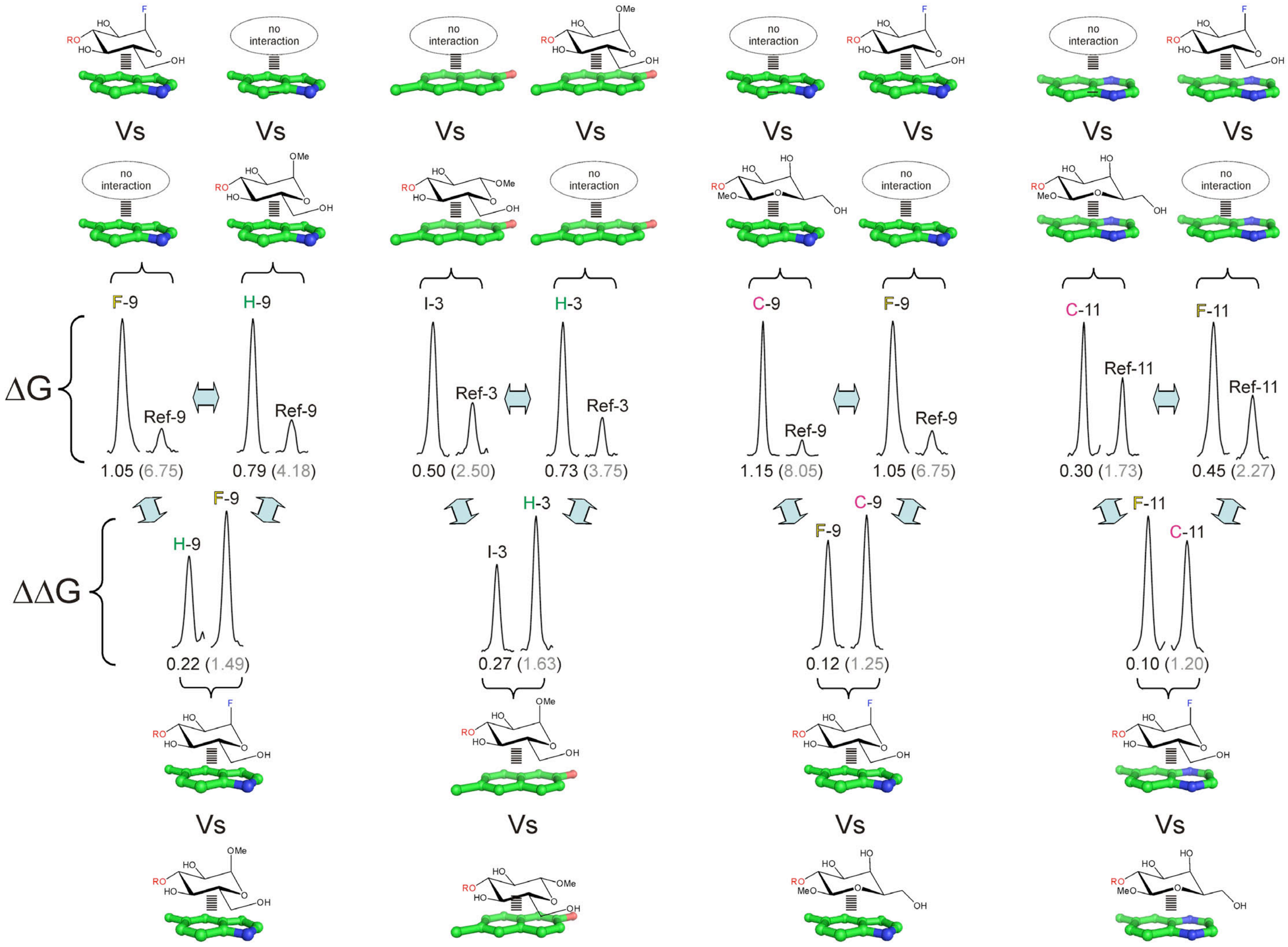

Figure S5.- Chemical shift perturbations promoted by the aromatic ring at the interacting D-pyranose unit in selected complexes. These values were taken as indicative of the interaction geometry.

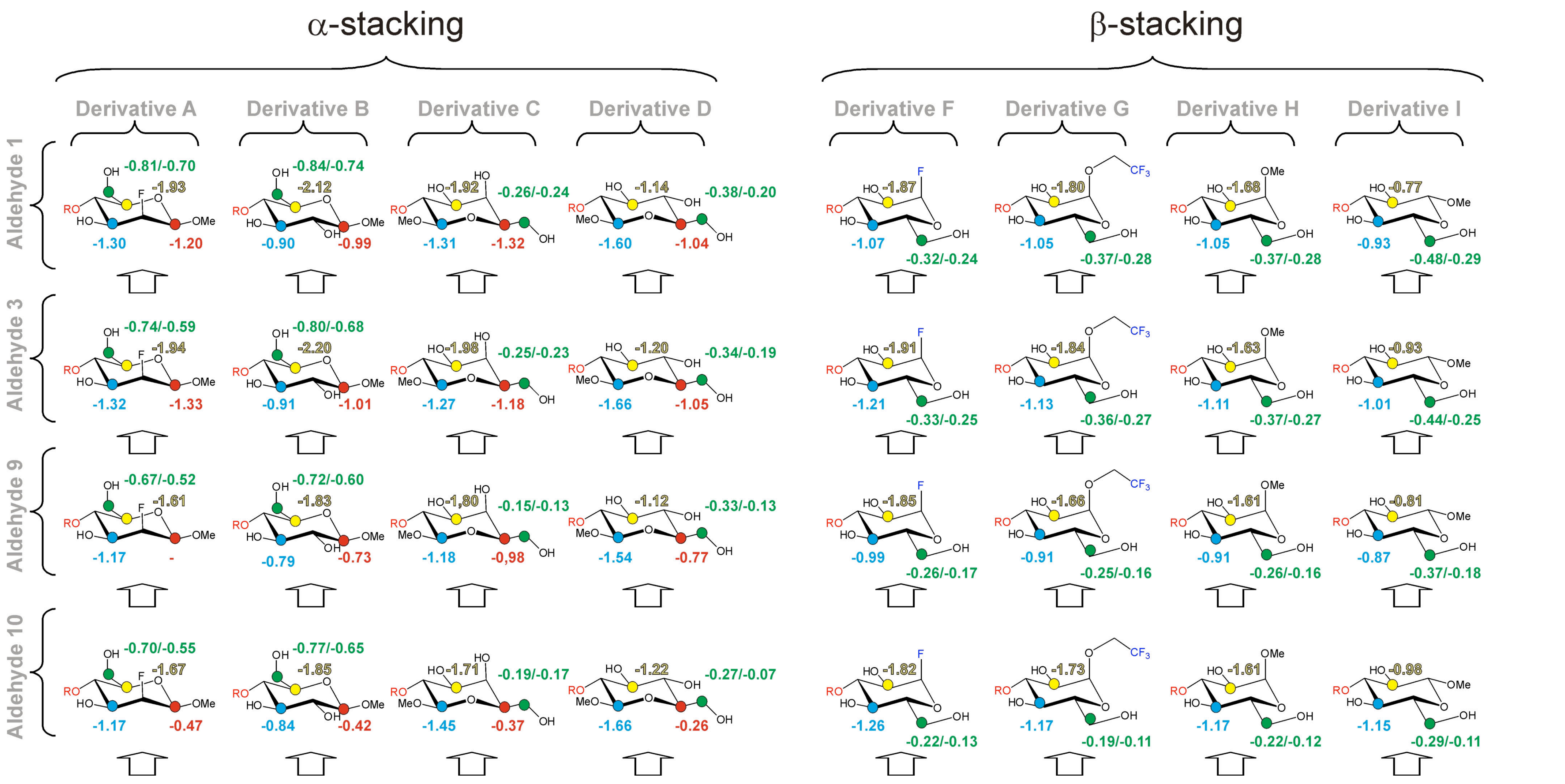

Figure S6.- Chemical shift perturbations promoted by the aromatic ring at the interacting D-pyranose unit in complexes **B-1**/**B-3** (left) and **C-1**/**C-3** (right). According to these data, the hydroxymethyl group (highlighted with a red ellipse) participates in contacts with the aromatic unit only for **B** complexes.

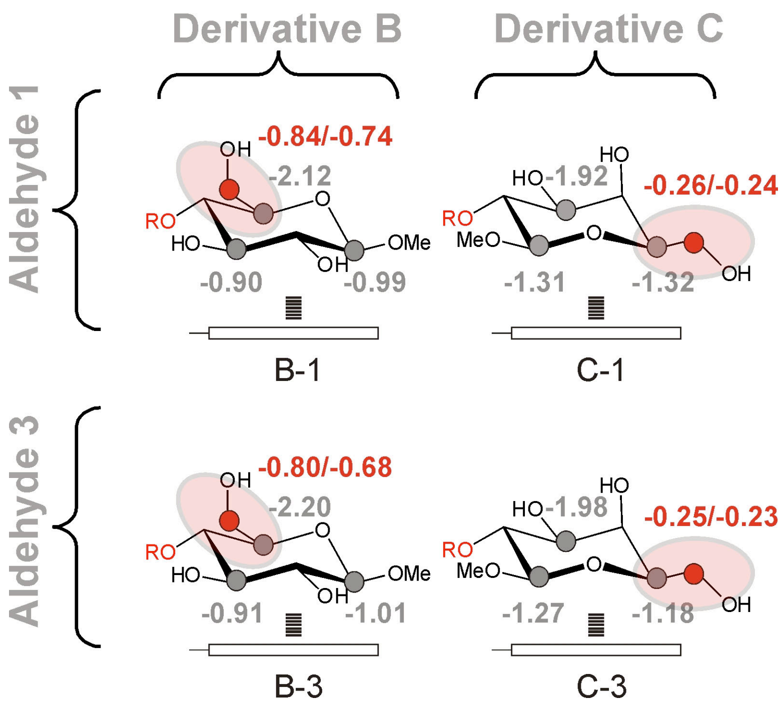

Figure S7.- The magnitude of the energy penalty associated to the axial/equatorial inversion of a pyranose polar group (highlighted with a green ellipse) depends on the precise geometrical features of the complex, varying from 0.3 kcal/mol, (**C-1** vs **D-1** or **C-3** vs **D-3**) to <0.1 kcal/mol (**A-1** vs **B-1** or **A-3** vs **B-3**). Measured Δδ values indicate that for **C** and **D** complexes the aromatic ring is closer to the inverted position.

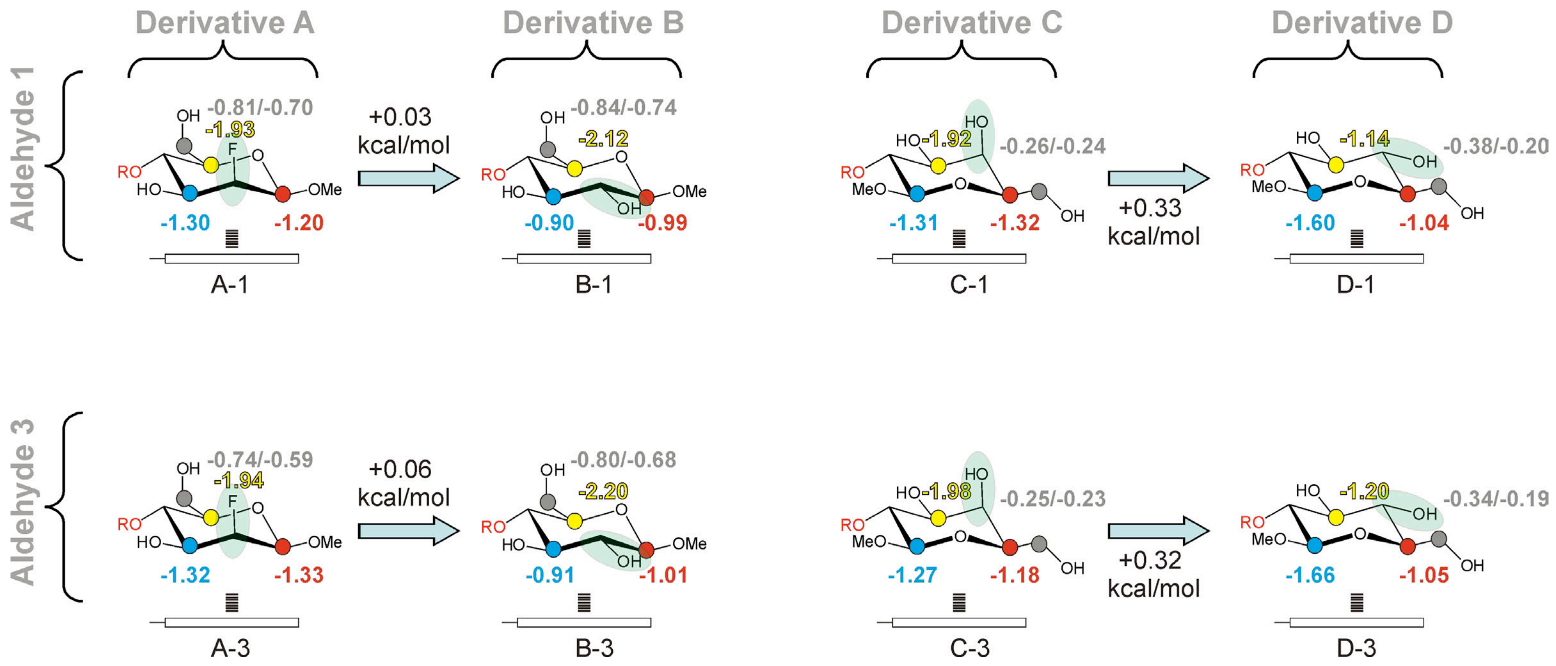

Figure S8.- To estimate the geometrical features of the different pyranose/aromatic complexes we performed 100 ns Molecular Dynamics (MD) simulations in the present of explicit solvent (see the Experimental Section). Selected ensembles are shown below.

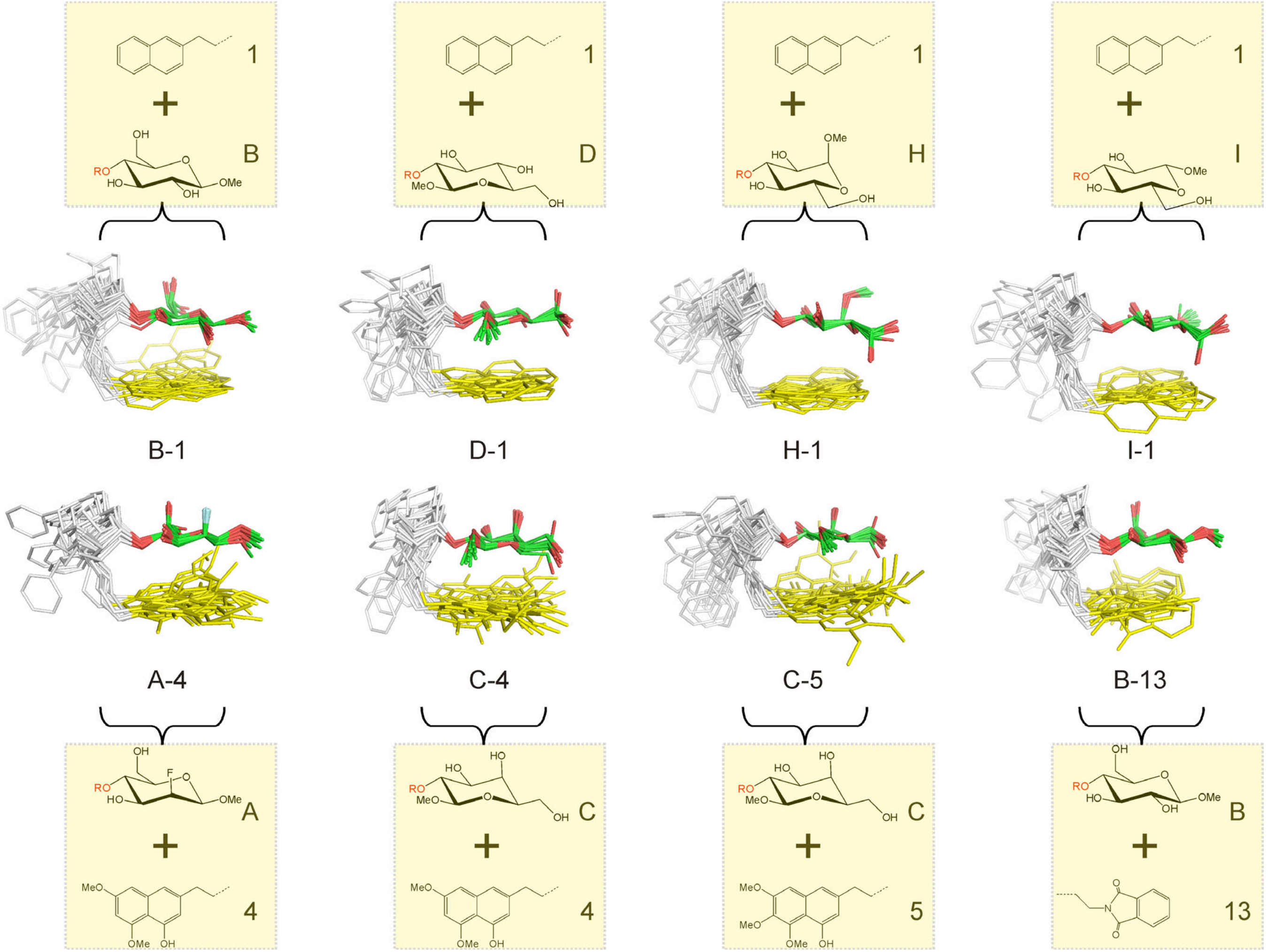

Figure S9.- Experimental free energies (ΔG_{exp}) measured for complexes formed by derivatives **C**, **D** or **F** with substituted nahpfthyl or phenyl rings (Right). Overall, the stability of the CH/π bonds increases with the electron-rich character of the aromatic unit. However, it can be observed that those complexes formed by densely oxygenated aromatic systems as **5**-**7** display reduced ΔG_{exp} values. Interestingly, quantum mechanics calculations show that the OR groups present in **5**-**7** are not co-planar with the naphthyl unit (so that multiple conformations are feasible). Although merely speculative, this peculiarity might oppose binding by, imposing a significant entropic penalty on the recognition process (Left).

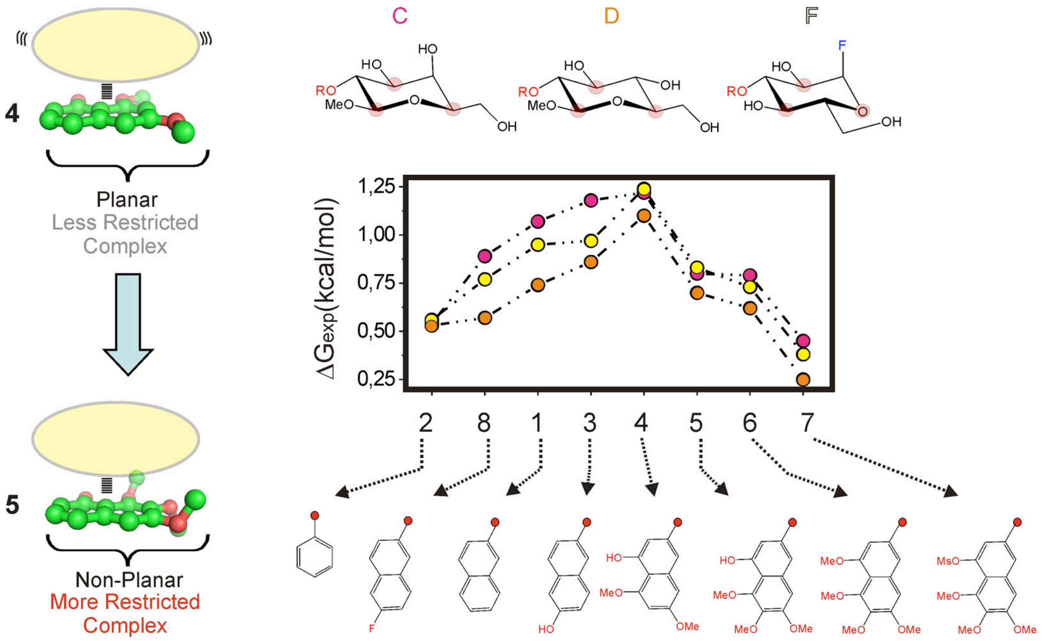

Figure S10.- Experimental ΔG_{exp} values measured for derivatives **C**(orange) and **D** (magenta)are represented against the gas phase interaction energies and the different contributions calculated with SCS-MP2/6-311G(2d,p) (Left) and M06-2X/TXVP (Right) levels for the THP/aromatic complexes (see the main text). Linear fits (red lines) and correlation coefficients (R, in grey) are shown in all cases.

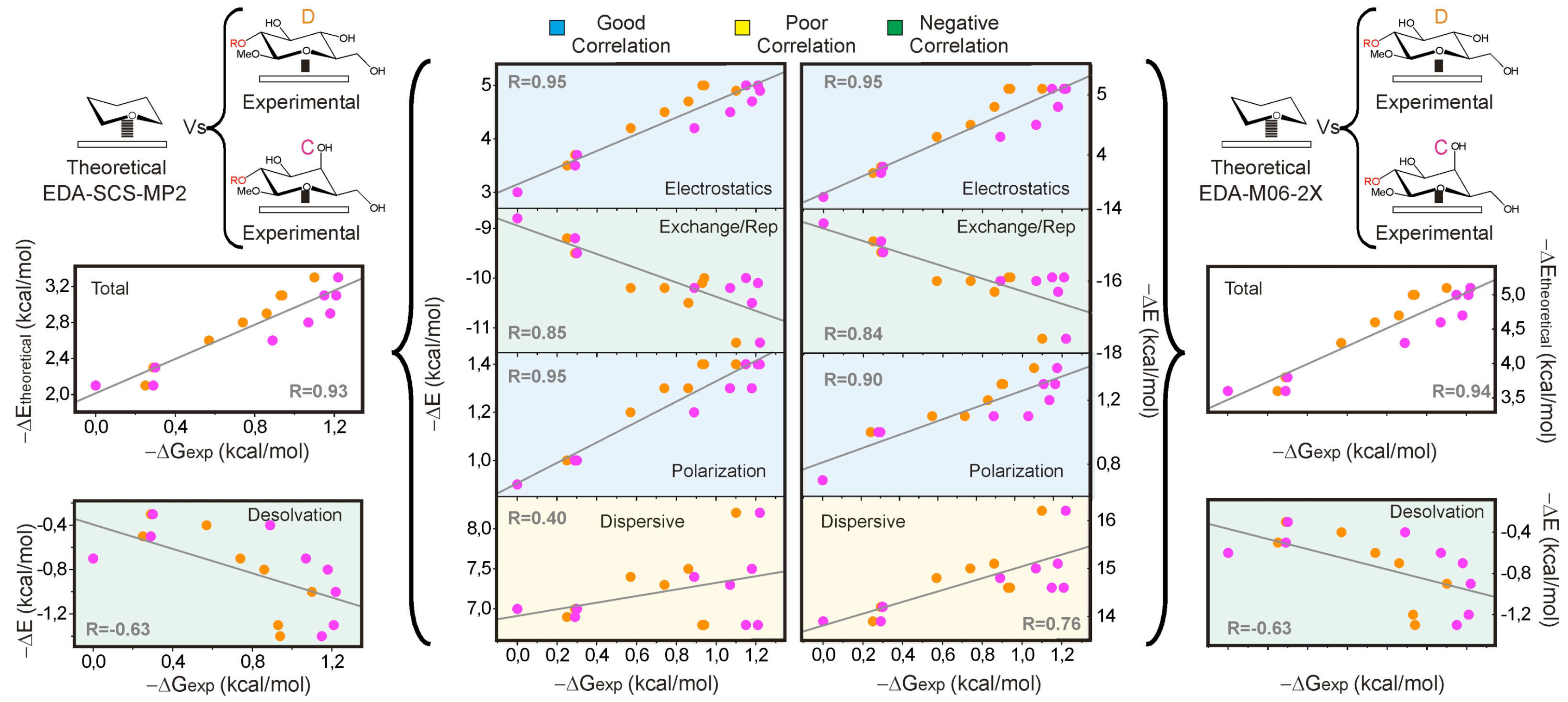

Figure S11.- a) Pairwise competition experiments performed with pair **C**/**Ref** and aldehyde **3** in the presence of organic co-solvents (indicated in black). NMR signals for the altrose anomeric proton in the final products are shown. Product ratios (in brackets) together with the **C-3** complex stabilities under the different solvent conditions are indicated in grey. A representation of the measured stabilities versus the cohesive energy densities of the different mixtures is shown in the bottom-right corner. Linear fit and correlation coefficient (R) are shown. b) Interaction energies for different solvent/nahthalene complexes in the gas phase, estimated at the M06-2X and SCS-MP2 levels.

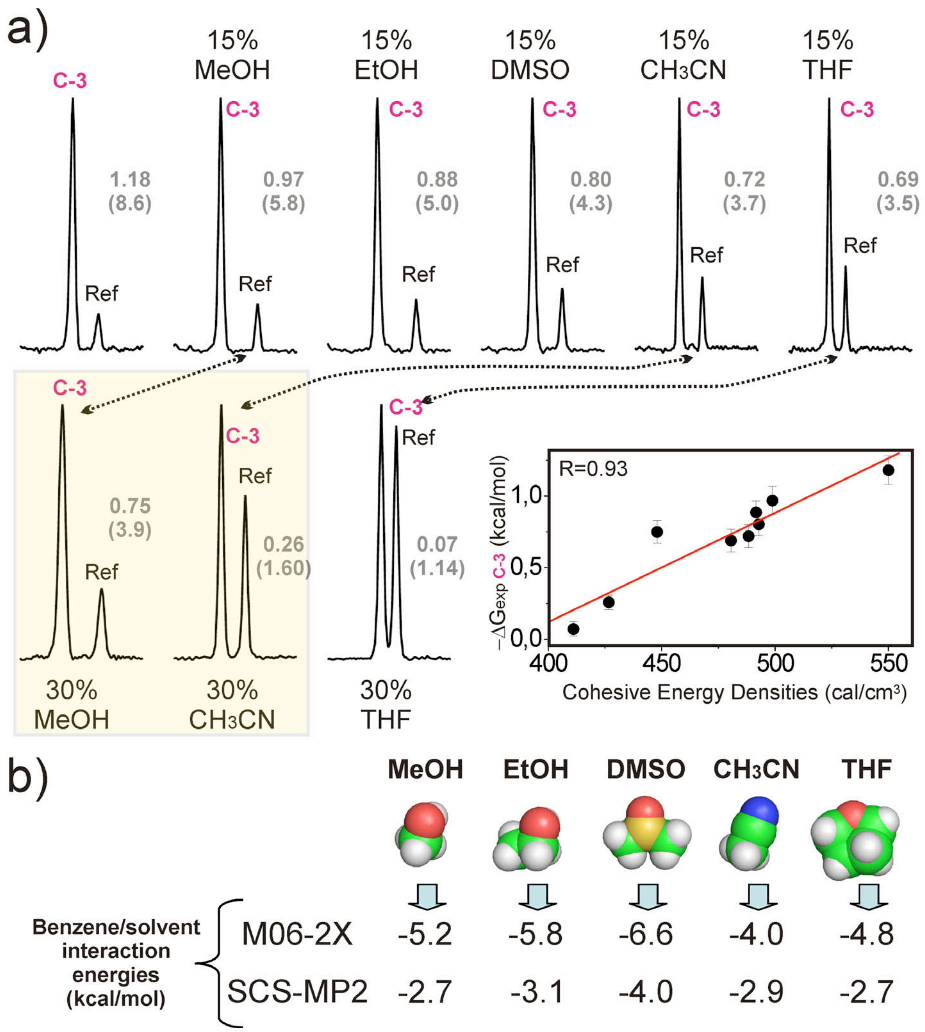

Supplement: Supplementary file 1 [file SC-006-C5SC02108A-s001.pdf]
